# Recent advances in the transformation reactions of the Betti base derivatives

**DOI:** 10.1039/d4ra01256a

**Published:** 2024-04-12

**Authors:** Abolfazl Olyaei, Mahdieh Sadeghpour

**Affiliations:** a Department of Chemistry, Faculty of Science, Imam Khomeini International University Qazvin Iran Olyaei_a@sci.ikiu.ac.ir; b Department of Chemistry, Qazvin Branch, Islamic Azad University Qazvin Iran

## Abstract

Betti bases are the products resulting from the one-pot multicomponent reaction involving 1-naphthol/2-naphthol, aliphatic/aromatic aldehydes, and secondary amines. This chemical process is commonly referred to as the Betti reaction. The significance of Betti bases in medicinal chemistry has grown substantially due to their diverse array of pharmacological applications. Furthermore, their synthetic utility is considerable, given their use as catalysts and ligands in asymmetric synthesis. Moreover, Mannich products, incorporating diverse functional groups such as –OH and –NH, find application in a range of organic reactions. This utilization enables the synthesis of novel C–C bond linkages and diverse heterocycles, including biologically active naphthoxazines, which hold biological applications such as antibacterial, antifungal and anticancer. The focus of this review article is on the application of aminonaphthol derivatives in transformation reactions and the synthesis of organic compounds, with particular emphasis on heterocycles.

## Abstract 2

This review article highlights the use of Betti base derivatives in transformation reactions for synthesizing organic compounds, particularly heterocyclic molecules, and investigation into their pharmacological properties.

## Graphical abstract

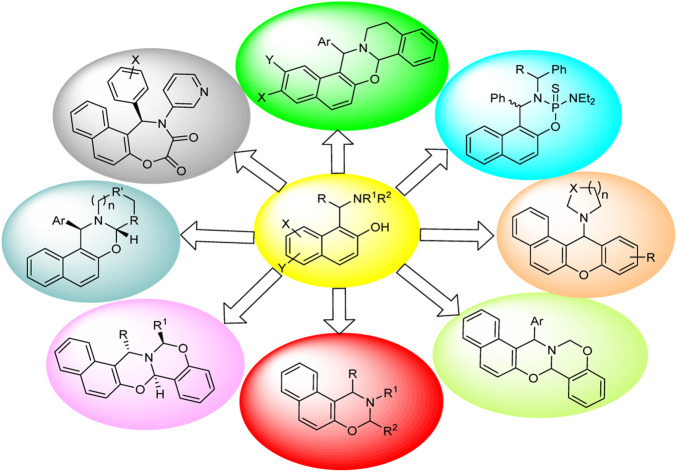

## 1 Introduction

**Fig. 1:**
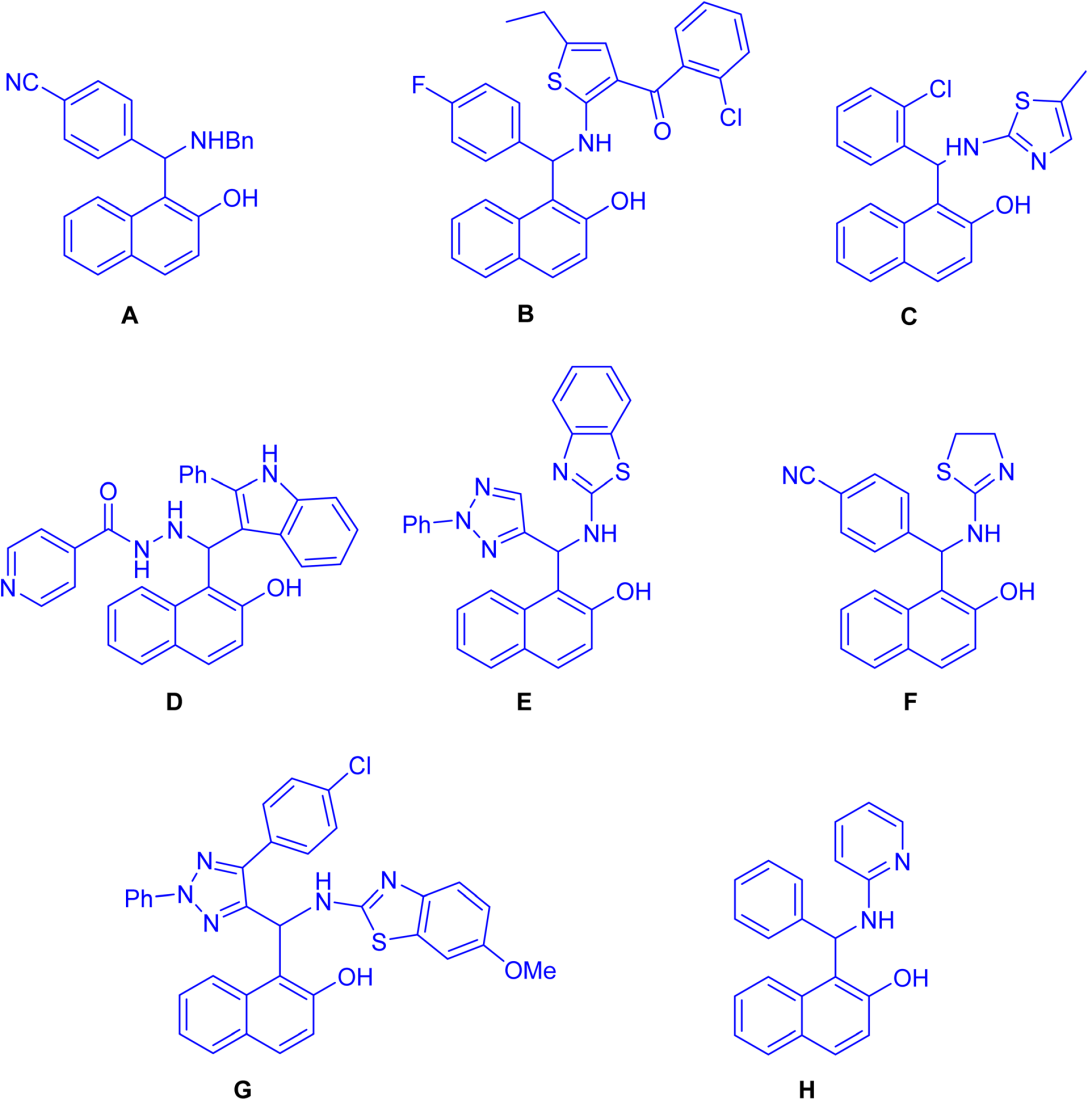
Structures of Betti bases exhibiting biological activities.

Straightforward synthesis of 1-(α-aminobenzyl)-2-naphthol (the Betti base) from 2-naphthol, benzaldehyde, and ammonia was reported for the first time by Betti at the beginning of the 20th century.^1–4^ The Betti procedure can be understood as an expansion of the Mannich condensation. In this process, formaldehyde is substituted with an aromatic aldehyde, the secondary amine is replaced by ammonia, and the C–H acid is substituted with an electron-rich aromatic compound, such as 2-naphthol. This modification in reactants results in a distinct chemical transformation, broadening the scope of the traditional Mannich condensation and yielding unique products through the Betti procedure. As a result of the potential utility of Mannich-type phenolic bases, the aminoalkylation of naphthol derivatives is a subject of current chemical interest. In recent years, numerous methodologies have reported for synthesizing these aminobenzylnaphthols, also known as Betti bases, and their bis-Betti base derivatives. These methods involve the utilization of diverse substrates such as various types of naphthols, aromatic amines, heteroaromatic amines, aliphatic and cyclic amines, instead of ammonia or diamines. Additionally, aliphatic and aromatic aldehydes or dialdehyde compounds have been employed under various conditions in recent research efforts. The typical process for the formation of the Betti bases begins with the generation of an *ortho*-quinone methide (*o*-QM) through the reaction between naphthol and aldehyde. Subsequently, the *o*-QM undergoes a Michael addition with an amine, resulting in the formation of the Betti base. These compounds exhibit a wide range of applications, including: enantioselective addition of diethylzinc to aldehydes,^5^ crystallization-induced diastereoisomer transformation,^6^ asymmetric hydrogenation and allylic substitution,^7^ optoelectronic applications (fluorescent chemosensors,^8^ fluorescence detection of Hg^2+^ and Cr^3+^ ions^9^ and electrochemical activity^10^) and biological properties such as anticancer agents^11^ (A), antioxidant^12^ (B), anti-bacterial^13^ (C), antitubercular agents^14^ (D), pesticidal agents^15^ (E), anti-Alzheimer agents^16^ (F), topoisomerase I inhibitors^17^ (G) and DNA binding and cleavage activity^18^ (H) as depicted in Fig. 1. Active –OH & –NH functionalities in Betti bases are employed in different organic reactions to synthesize new C–C bond linkages and various medicinally important heterocycles such as biologically active 1,3-oxazine and naphthoxazine derivatives. Because of their broad significance in the medicinal and pharmaceutical industries, research on the synthesis of Betti bases has been ongoing since the early twentieth century. Up to now, a limited number of reviews have been published covering the synthesis of Betti base derivatives and their applications. Indeed, there has been limited discussion on the transformation of these compounds into various organic compounds. The exploration of subsequent reactions and the diversification of Betti base derivatives into different organic compounds is an aspect that has received relatively less attention in the existing review papers.^19–24^ In this review, we aim to offer a comprehensive overview of a wide range of methodologies pertaining to the transformation of Betti base derivatives in the synthesis of various organic compounds. Our review examines the diverse strategies employed in these transformations and explores the applications of the resulting organic compounds.

## 2 Synthesis of naphthoxazines

**Scheme 1:**
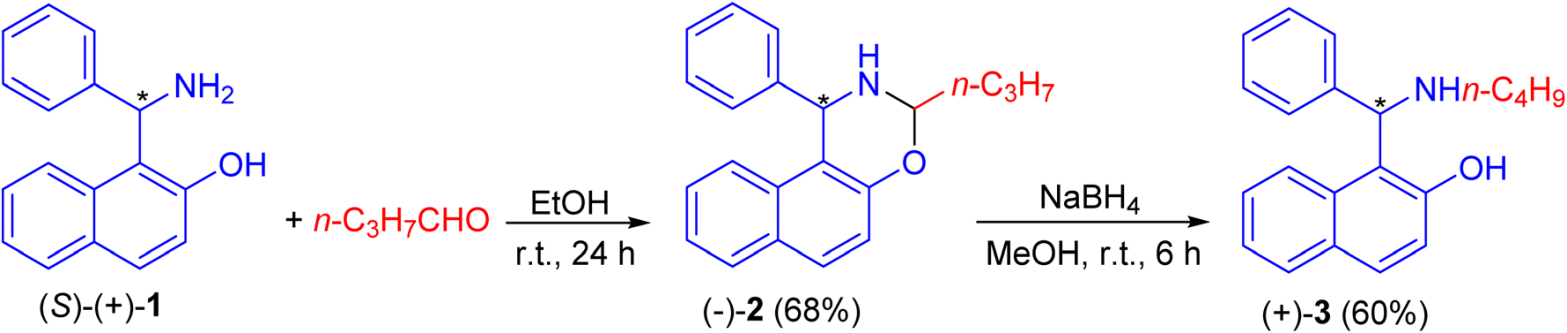
Preparation of l-(α-*N*-butylaminobenzyl)-2-naphthol (*S*)(+)-3.

In 1999, Naso and co-workers reported the reaction of Betti base (*S*)-(+)-1 with *n*-butanal in EtOH at room temperature for 24 h to produce naphthoxazine (−)-2 in 68% yield. It was reduced to l-(α-*N*-butylaminobenzyl)-2-naphthol (*S*)(+)-3 in 60% yield with NaBH_4_ in CH_3_OH at room temperature for 6 h (Scheme 1).^25^

**Scheme 2:**
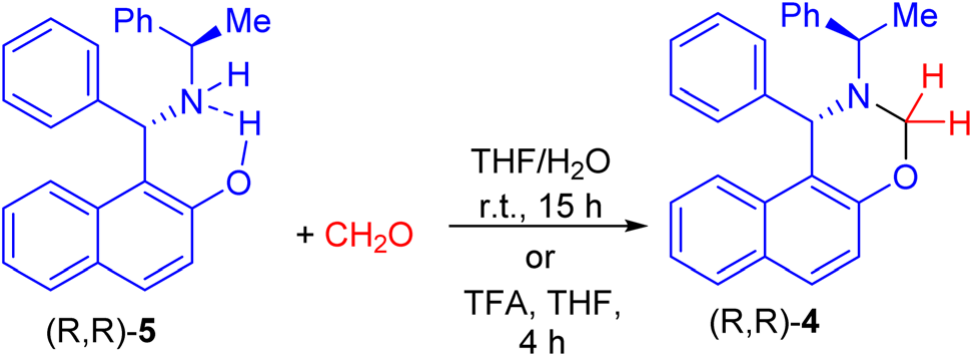
Synthesis of naphthoxazine 4.

After that, Palmieri *et al.* described synthesis of naphthoxazine 4 by the reaction of enantiopure aminonaphthol 5 with formaldehyde in THF/H_2_O at room temperature for 15 h.^26^ Also, methylation of (*R*,*R*)-5 with formaldehyde in the presence of trifluoroacetic acid in THF at room temperature for 4 h afforded naphthoxazine derivative (*R*,*R*)-4 in 90% yield as depicted in Scheme 2.^27^

**Scheme 3:**
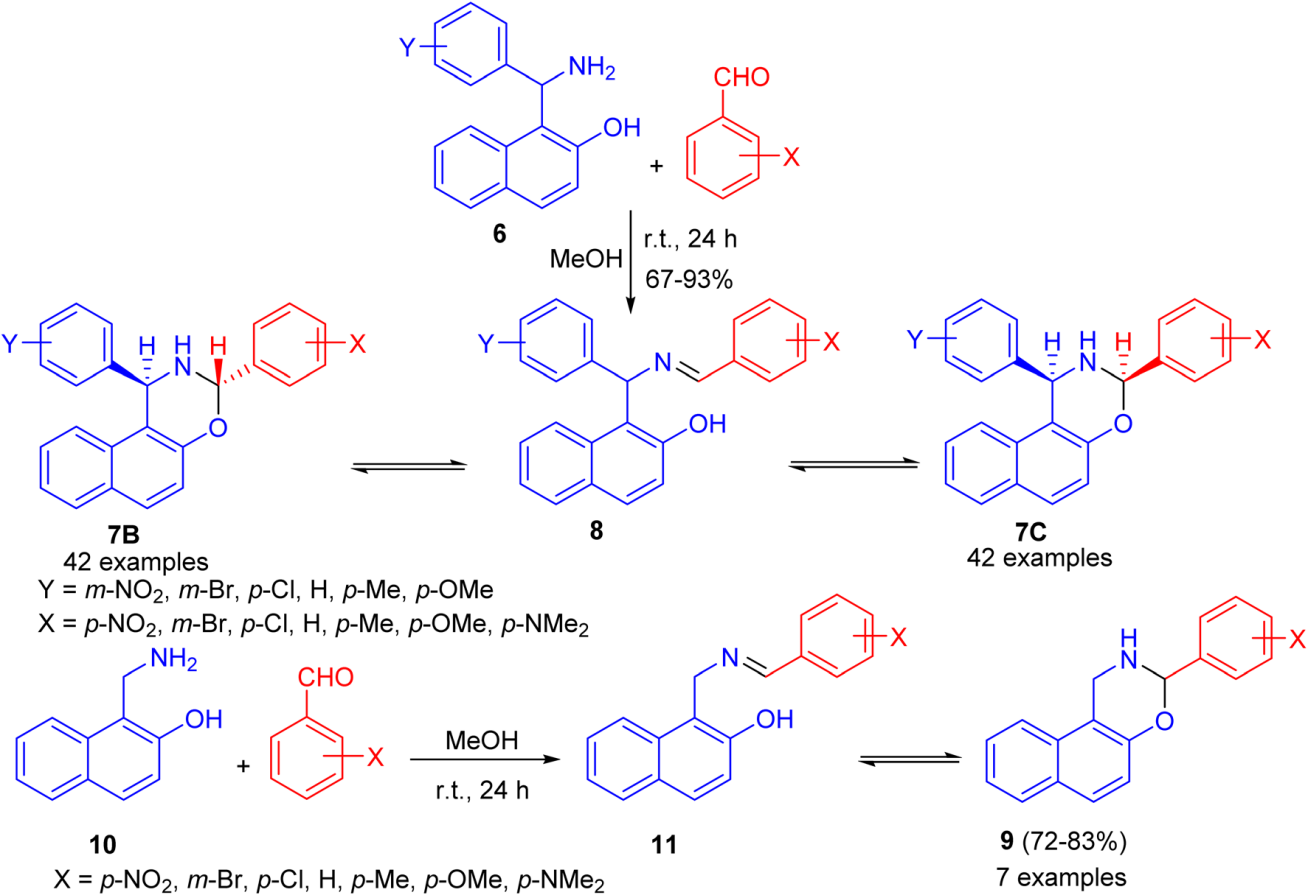
Synthesis of 1,3-diaryl-2,3-dihydro-1*H*-naphth[1,2-*e*][1,3]oxazines 7, 9.

After that, condensation of Betti base analogue amino naphthols 6 with substituted benzaldehydes in absolute MeOH at ambient temperature for 24 h led to the formation of 1,3-diaryl-2,3-dihydro-1*H*-naphth[1,2-*e*][1,3]oxazines 7 in 67–93% yields. The ^1^H-NMR spectra of 7 revealed that, in CDCl_3_ solution at 300 K, the members of each set of compounds 7 participated in three-component ring chain tautomeric equilibria containing C-3 epimeric naphthoxazines (7B and 7C) besides the open tautomer 8. For the 3-(*p*-dimethylaminophenyl)-substituted derivatives, the tautomeric equilibria contained only one ring-closed form (7B). Moreover, 3-aryl-2,3-dihydro-1*H*-naphth[1,2-*e*][1,3]oxazines 9 were also prepared in 72–83% yields from the readily available 1-aminomethyl-2-naphthol (10) and aromatic aldehydes in absolute MeOH at ambient temperature for 24 h. In CDCl_3_ at 300 K, compound 11 proved to participate in ring chain tautomeric equilibria. Moreover, the influence of aryl substituents at position on the ring-chain tautomeric equilibria described by the Hammett equation (Scheme 3).^28^

**Scheme 4:**
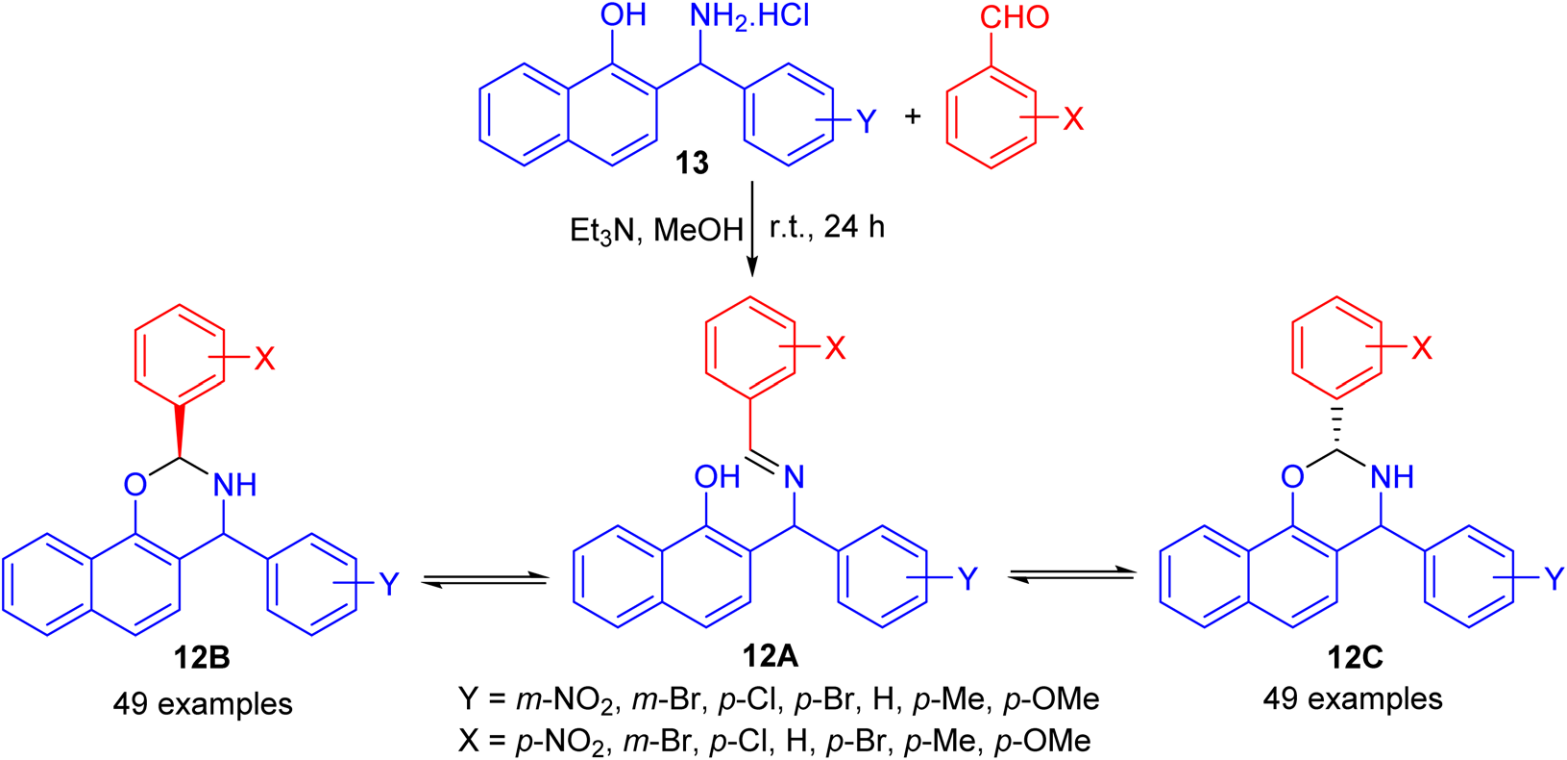
Synthesis of 2,4-diaryl-3,4-dihydro-2*H*-naphth-[2,1-*e*][1,3]oxazines 12.

In 2004, Fulop and co-workers synthesized 2,4-diaryl-3,4-dihydro-2*H*-naphth-[2,1-*e*][1,3]oxazines 12 by condensations of aminonaphthol hydrochlorides 13 with equivalent amounts of aromatic aldehydes in the presence of Et_3_N in absolute MeOH at ambient temperature for 24 h. The ^1^H-NMR spectra of 12 proved that, in CDCl_3_ solution at 300 K, the members of each set of compounds 12 formed three-component tautomeric mixtures, containing C-2 epimeric oxazines 12B and 12C together with the open-chain tautomer 12A as shown in Scheme 4.^29^

**Scheme 5:**
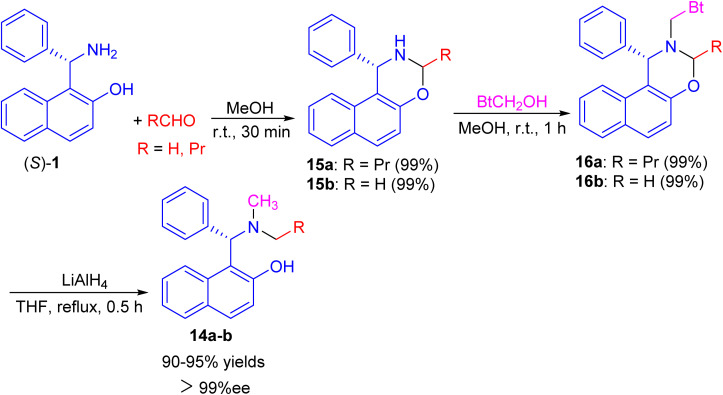
Synthesis of chiral *N*-methyl-*N*-alkyl Betti bases 14.

Next, a one-pot preparation of chiral *N*-methyl-*N*-alkyl Betti base 14 developed by highly regioselective *N*-alkylation of (*S*)-(+)-Betti base (*S*)-1. 2-(*R*)-4-Phenyl-naphtho[1,2-*e*][1,3]oxazine 15, obtained by condensation of (*S*)-1 and aldehyde in MeOH at room temperature for 30 min, which *N*-methylated with BtCH_2_OH under essentially neutral conditions to yield 2-(*R*)-*N*-benzotriazolylmethyl-4-phenyl-naphtho[1,2-*e*][1,3]oxazine 16 after 1 h. Chiral *N*-methyl-*N*-alkyl Betti base 14 was then obtained by simultaneously cleaving the C–Bt bond and C–O bond in the structure of 16*via* LiAlH_4_ in THF under reflux conditions for 0.5 h (Scheme 5).^30^

**Scheme 6:**
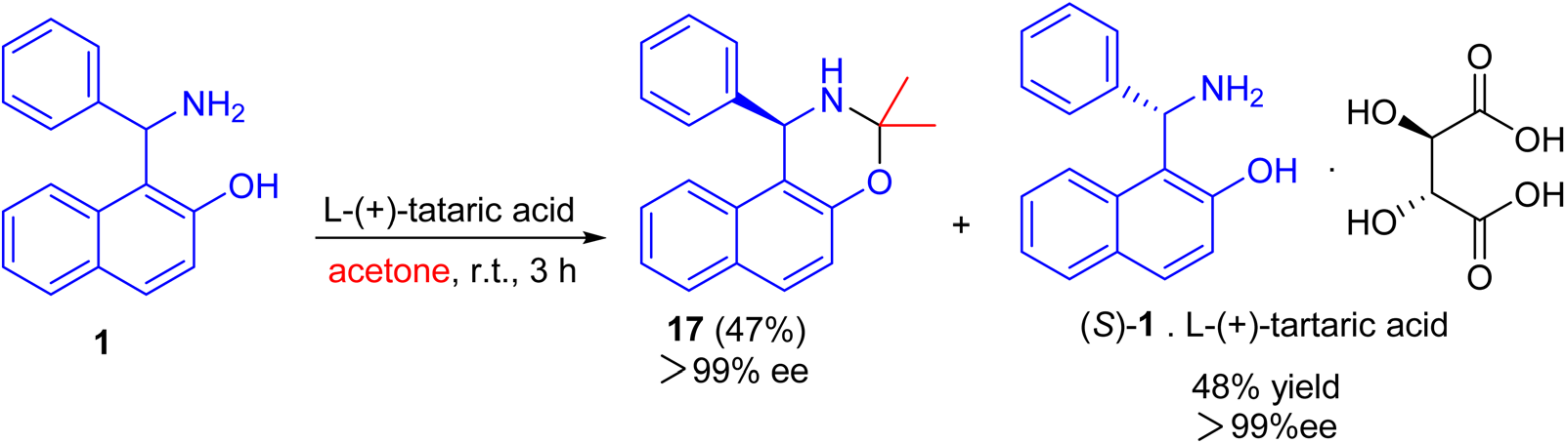
Kinetic resolution of racemic Betti base 1.

After that, an efficient kinetic resolution of racemic Betti base with l-(+)-tartaric acid in acetone was developed based on a enantioselective *N*,*O*-deketalization, by which the enantiopure *R*- and *S*-enantiomers of Betti base 1 were obtained as the corresponding *N*,*O*-ketal compound 17 and salt with l-(+)-tartaric acid, respectively, in excellent yields with a practically foolproof operation (Scheme 6).^31^

**Scheme 7:**
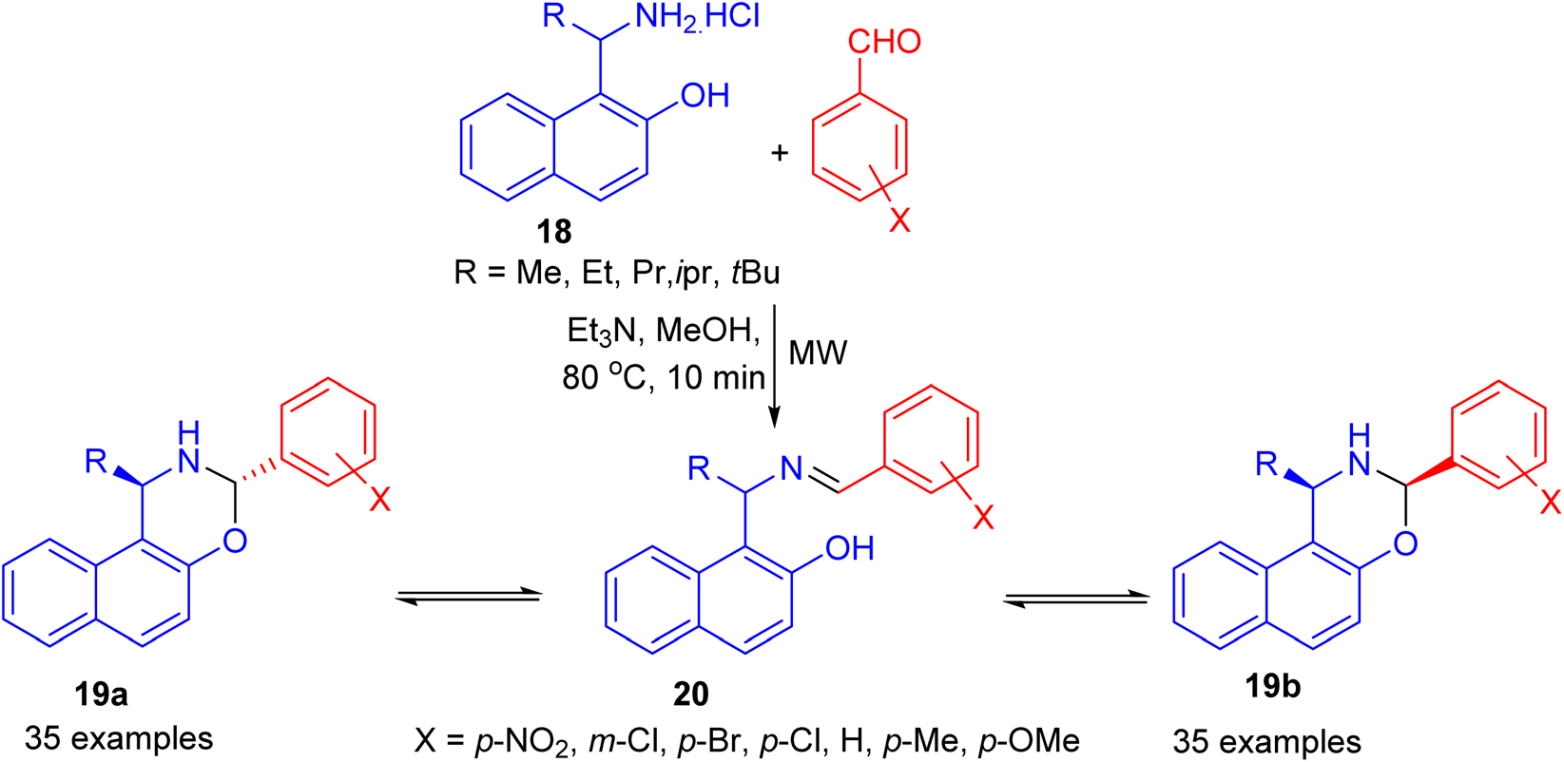
Preparation of 1-alkyl-3-aryl-2,3-dihydro-1*H*-naphth[1,2-*e*][1,3]oxazines 19.

In 2006, Fulop *et al.* developed condensation of aminonaphthol hydrochlorides 18 with substituted benzaldehydes under microwave irradiation in the presence of Et_3_N in MeOH at 80 °C for 10 min. The product 1-alkyl-3-aryl-2,3-dihydro-1*H*-naphth[1,2-*e*][1,3]oxazines 19 was obtained in 15–81% yields, which proved to be three-component tautomeric mixtures in CDCl_3_ at 300 K. The members of each set of compounds exist in three-component ring-chain tautomeric mixtures containing the C-3 epimericnaphthoxazines (19a and 19b) and also the open tautomer 20 as illustrated in Scheme 7.^32^

**Scheme 8:**
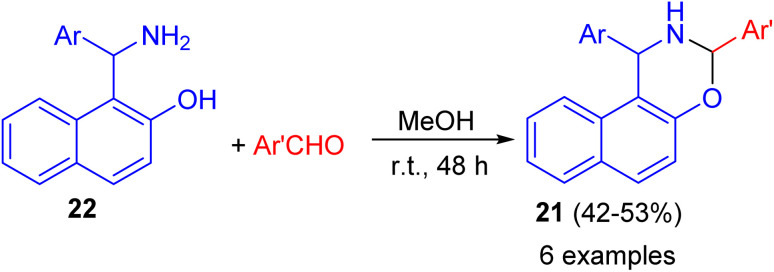
Synthesis of 1,3-disubstituted-2,3-dihydro-1*H*-naphth[1,2-*e*][1,3]oxazines 21.

In addition, 1,3-disubstituted-2,3-dihydro-1*H*-naphth[1,2-*e*][1,3]oxazines 21 prepared in 42–53% yields through the ring-closure reactions of the aminonaphthols 22 with substituted aryl- and heteroaryl aldehydes in MeOH at room temperature for 48 h as depicted in Scheme 8.^33^

**Scheme 9:**
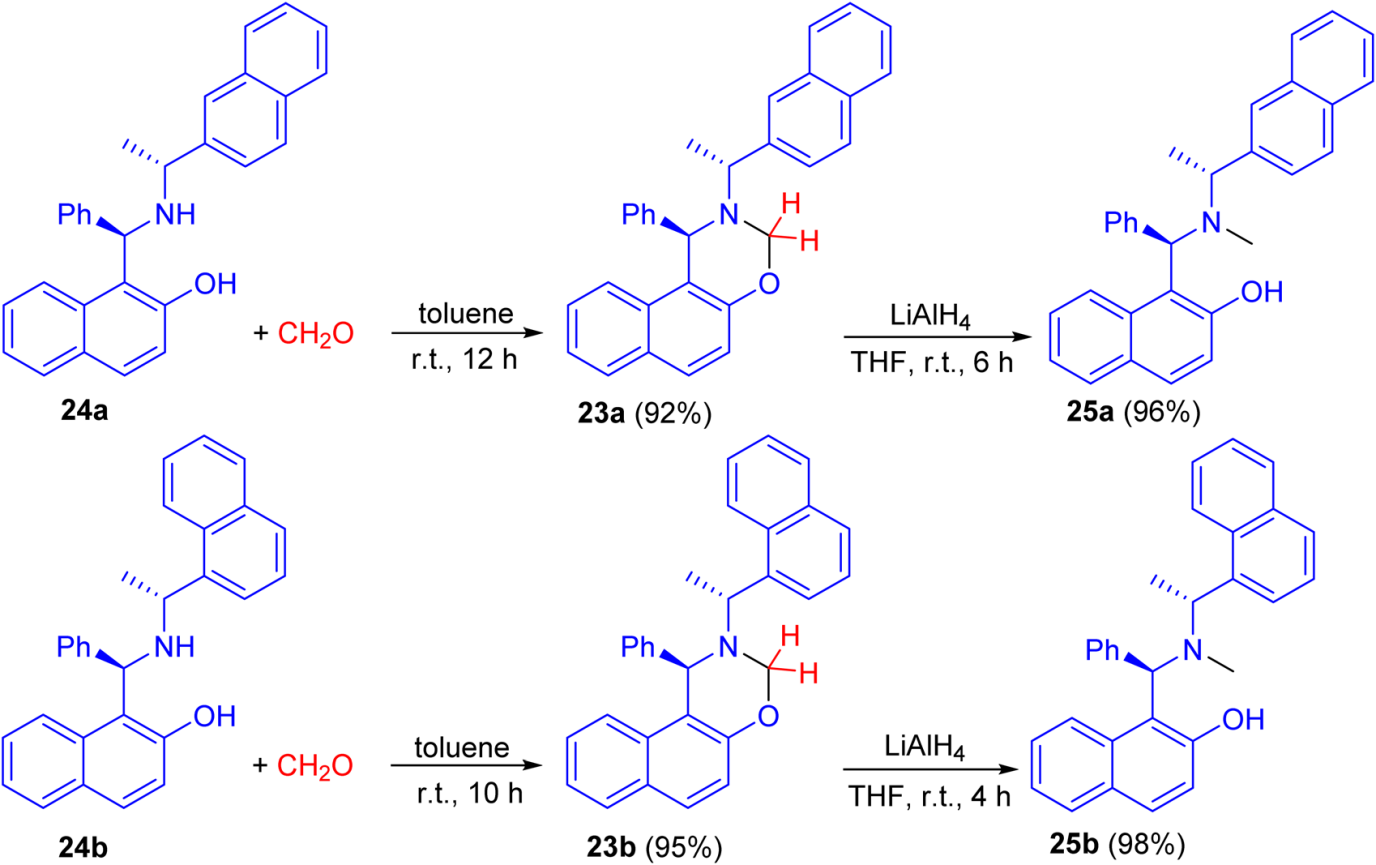
Synthesis of naphthoxazine derivatives 23 and chiral aminonaphthols 25.

In 2008, naphthoxazine derivatives 23a and 23b synthesized in 92–95% yields by the reaction of Betti base derivatives 24a and 24b with paraformaldehyde in toluene at room temperature for 10–12 h. The compound 23 was also reduced with LiAlH_4_ in THF at room temperature for 4–6 h affording aminonaphthol derivatives 25a and 25b in 96–98% yields (Scheme 9). The aminonaphthols 24 were found to catalyze the enantioselective ethylation of aryl aldehydes to 1-aryl-1-propanols (up to 92% ee).^34^

**Scheme 10:**
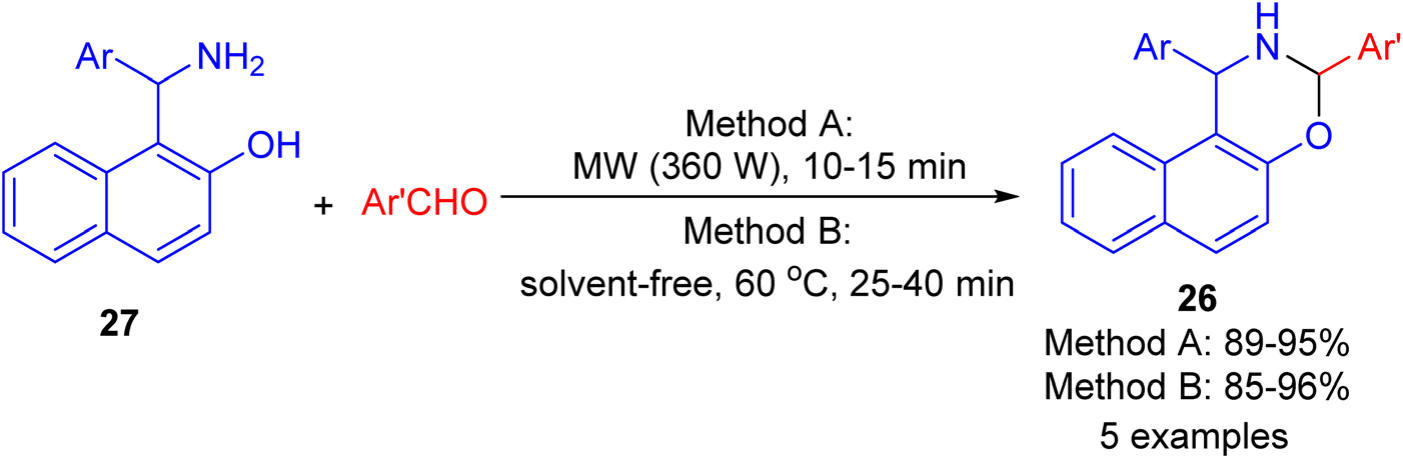
Synthesis of 1,3-disubstituted-2,3-dihydro-1*H*-naphth[1,2-*e*][1,3]-oxazines 26.

In 2009, synthesis of 1,3-oxazine derivatives 26 reported in 85–96% yields by the reaction of Betti base 27 with aromatic/heteroaromatic aldehydes under microwave irradiation (360 W) for 10–15 min or solvent-free conditions at 60 °C for 25–40 min. The simplicity of the reaction conditions, their efficiency, and the excellent results in shorter reaction times using both method A and method B under solvent and catalyst-free conditions, constitute an attractive contribution among the existing methodologies (Scheme 10).^35^

**Scheme 11:**
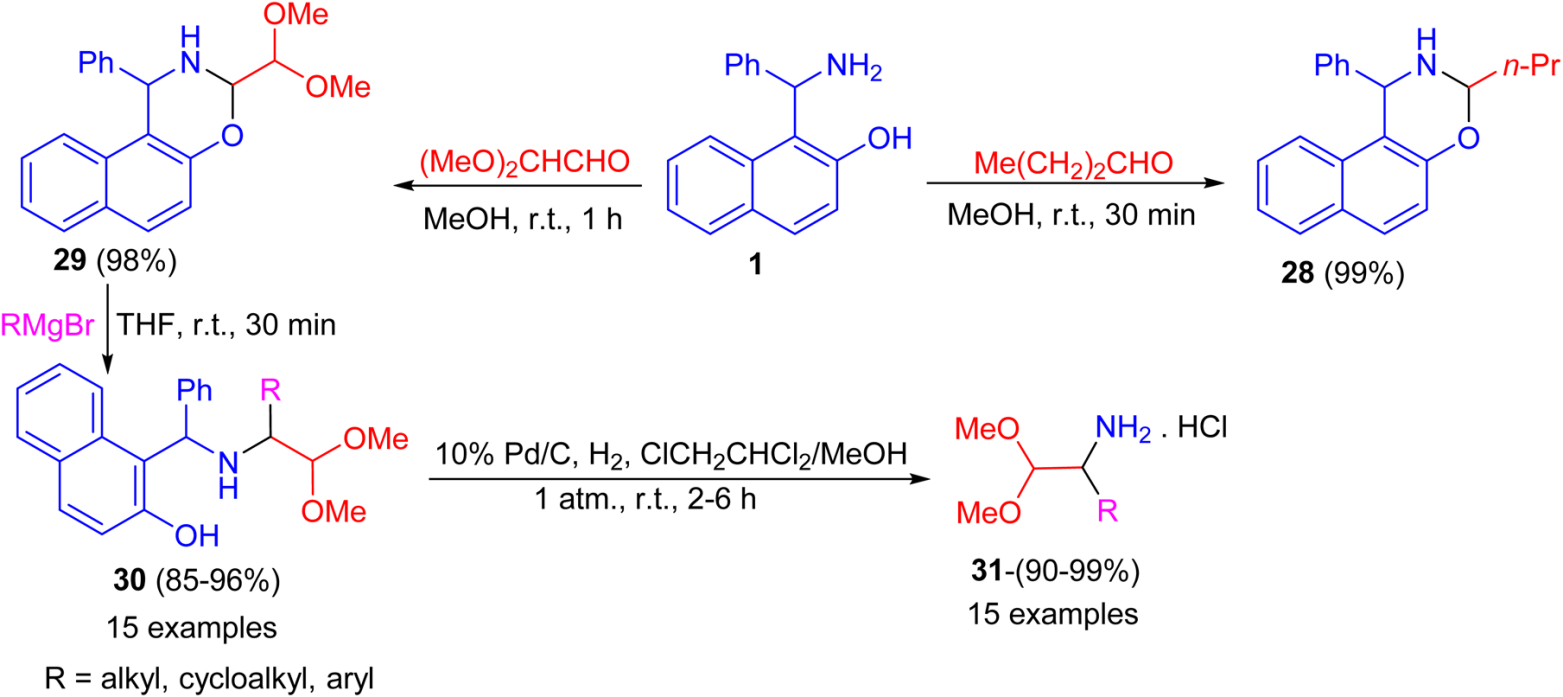
Preparation of naphthoxazines 28 and 29 and 1-substituted 2,2-dimethoxyethylamine hydrochlorides 31.

After that, the condensation of Betti base 1 and aliphatic aldehydes such as 1-butanal and (MeO)_2_CHCHO in MeOH at room temperature for 30–60 minutes afforded the corresponding *N*,*O*-acetal 28 and 29 in almost quantitative yields even without the use of an acidic catalyst. Also, alkylation of 29 by RMgBr was accomplished quickly in THF at room temperature within 30 minutes to give the desired product 30 in 85–96% isolated yield. Then, the diaryl benzylamine 30 was *N*-debenzylated quantitatively under mild Pd/C catalytic hydrogenolysis conditions in ClCH_2_CHCl_2_/MeOH at room temperature for 2–6 h, afforded 1-substituted 2,2-dimethoxyethylamine hydrochlorides 31 in 90–99% yields. The method reported is extremely convenient and highly efficient with wide substrate scopes (Scheme 11).^36^

**Scheme 12:**
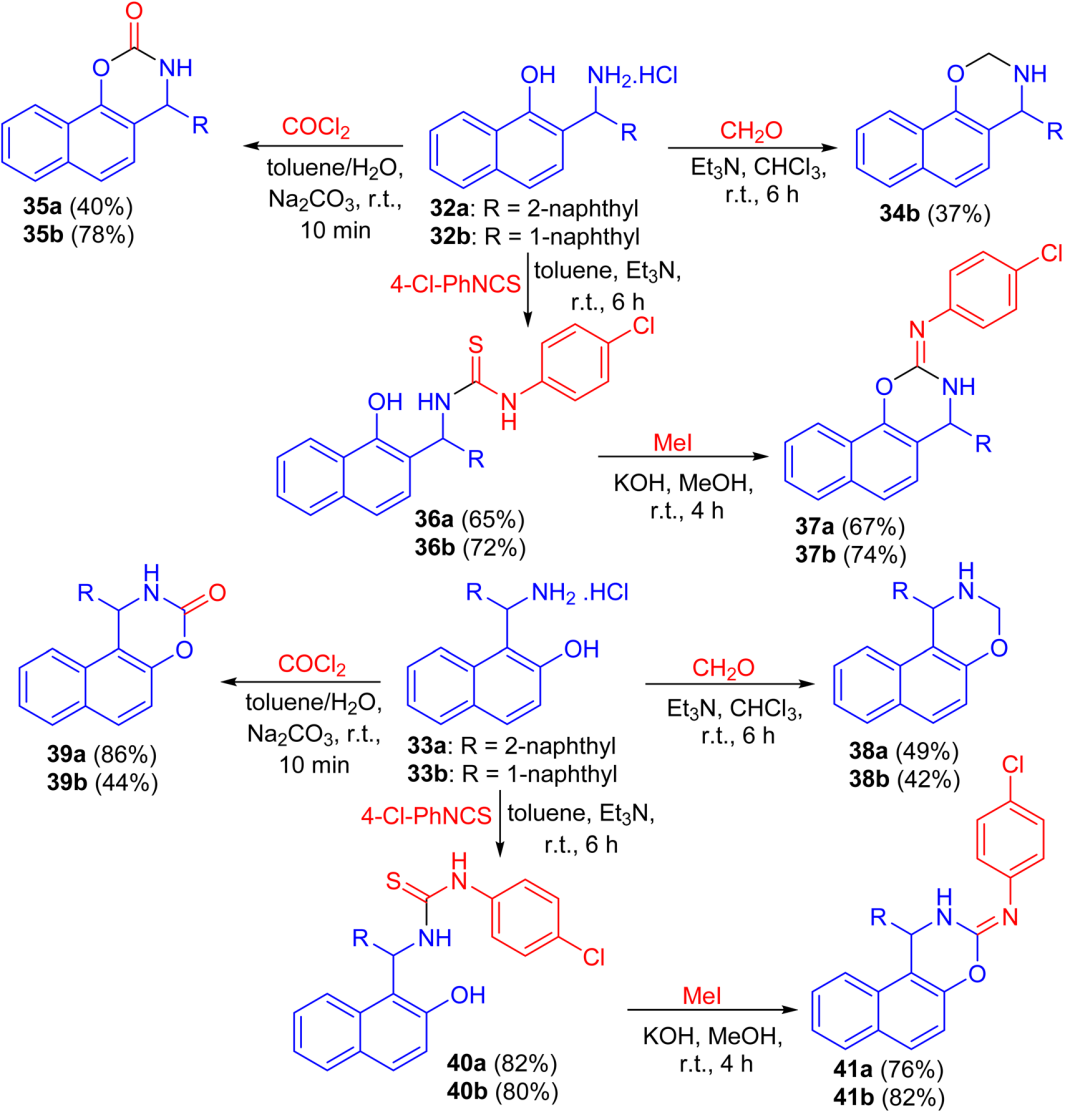
Synthesis of naphthoxazine derivatives 34, 35, 38, 39 and 41.

Next, the reaction of aminomethylnaphthols 32a and 32b and 33a and 33b with paraformaldehyde using Et_3_N in CHCl_3_ for 6 h, phosgene in toluene/H_2_O in the presence of Na_2_CO_3_ for 10 min and 4-chlorophenyl isothiocyanate in the presence of Et_3_N for 6 h followed by the reaction with MeI in MeOH using KOH at room temperature for 4 h led to naphthoxazine derivatives 34, 35, 38, 39 and 41 as shown in Scheme 12.^37^

**Scheme 13:**
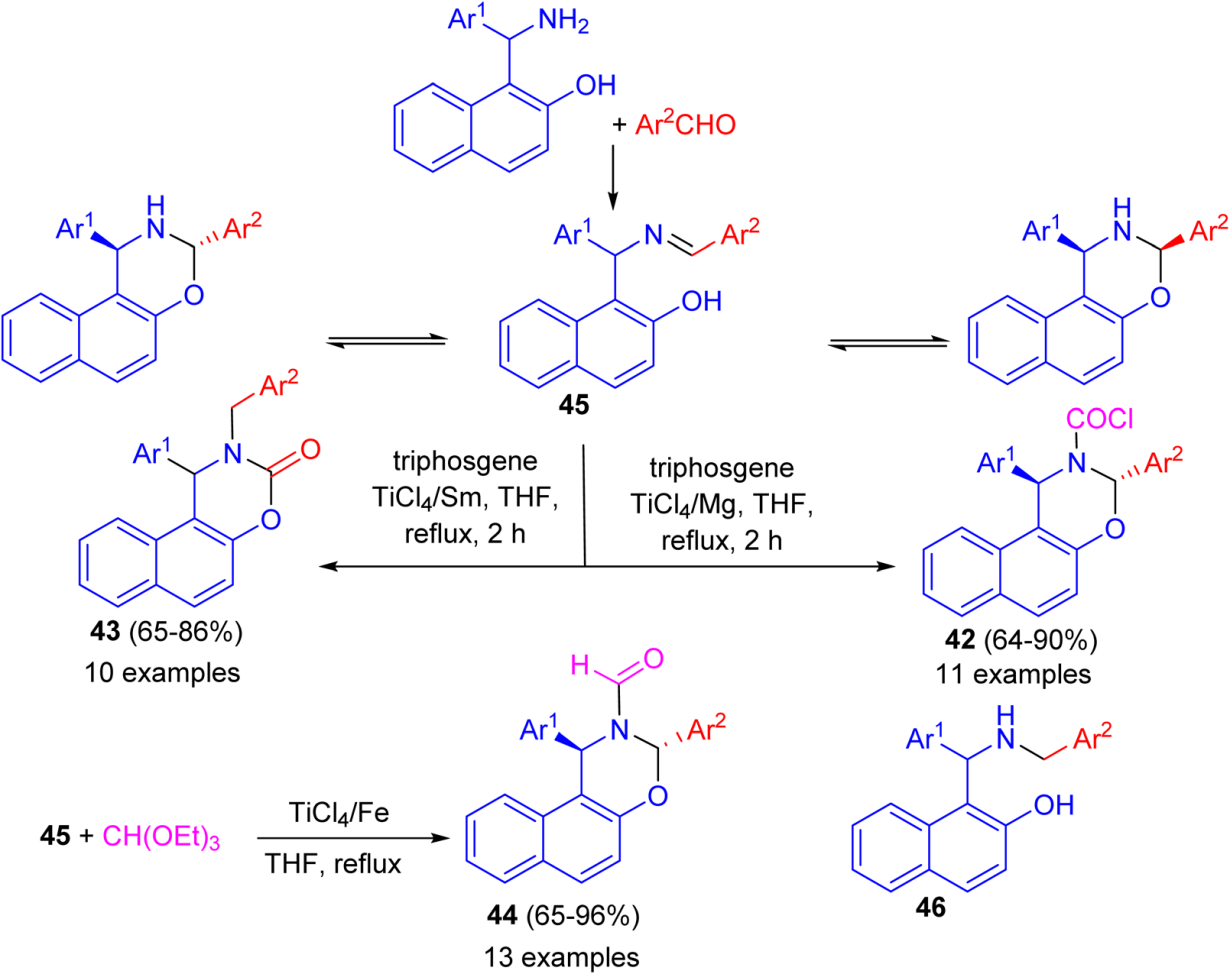
Synthesis of naphtho[1,2-*e*][1,3]oxazine derivatives 42–44.

In 2010, Shi and co-workers described synthesis a series of naphtho[1,2-*e*][1,3]oxazine derivatives such as *trans*-1,3-diaryl-1*H*-naphtho[1,2-*e*][1,3]oxazine-2(3*H*)-carbonyl chloride 42, 1-aryl-2-benzyl-1,2-dihydronaphtho[1,2-*e*][1,3]oxazine-3-one 43, and *trans*-1,3-diaryl-1*H*-naphtho[1,2-*e*][1,3]oxazine-2(3*H*)-carbaldehyde 44 in 65–96% yields *via* a chemoselective reaction of compound 45 with triphosgene or triethyl orthoformate in THF under reflux conditions, respectively, induced by different low-valent titanium/M systems. In these reactions, when the TiCl_4_/Mg system was used, TiCl_4_ is reduced by Mg dust to give low-valent titanium species, which catalysed the reaction of ring-closed tautomer and triphosgene to give product 42. When the TiCl_4_/Sm system was used, TiCl_4_ is reduced by Sm to give low valent titanium species and Sm^2+^. In the initial step, the open tautomer was reduced by Sm^2+^ and low-valent titanium to aminophenol intermediate 46. Then products 42 were obtained by the reaction of 46 and triphosgene catalyzed by low-valent titanium species. This method has the advantages of short reaction time, high chemoselectivity, accessible materials, and convenient manipulation (Scheme 13).^38^

**Scheme 14:**
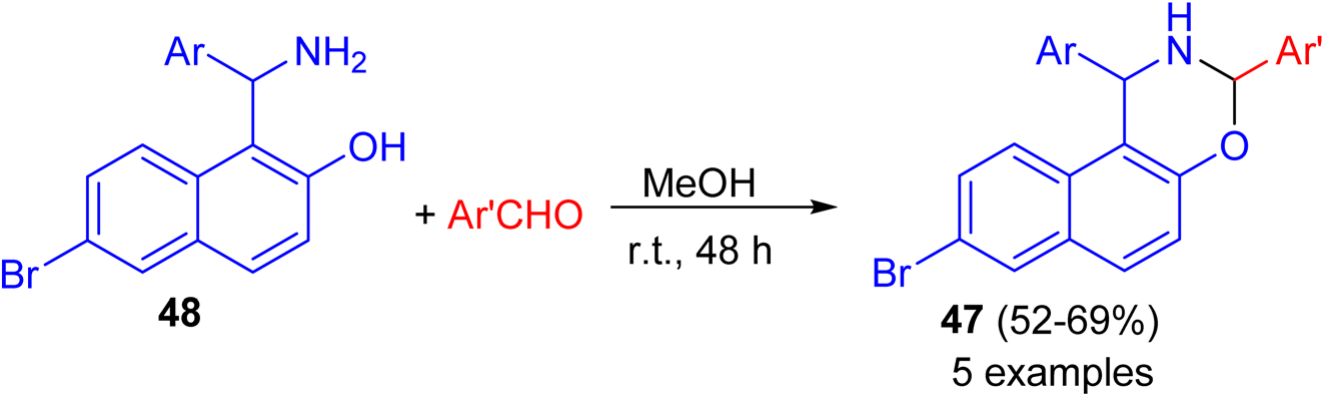
Synthesis of 8-bromo-1,3-diaryl-2,3-dihydro-1*H*-naphtho[1,2-*e*][1,3]oxazines 47.

After that, a series of 8-bromo-1,3-bis(aryl)-2,3-dihydro-1*H*-naphtho[1,2-*e*][1,3]oxazines 47 synthesized in 52–69% yields by the reaction of Betti base 48 with substituted aryl and heteroarylaldehydes in MeOH at ambient temperature for 48 h (Scheme 14). Some of the compounds found to exhibit good activity against tested bacterial and fungal strains. Compounds having fluoro, chloro and methyl substituted phenyl group attached to naphthoxazine showed promising activity.^39^

**Scheme 15:**
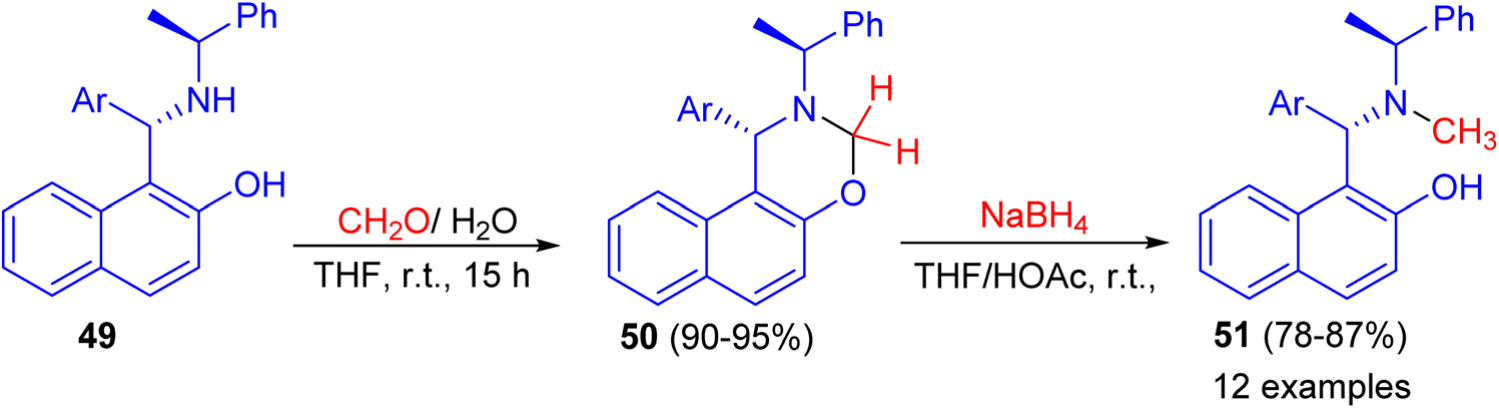
The preparation of chiral aminonaphthol ligands 51.

In addition, the reaction of aminonaphthol derivatives 49 with formaldehyde in THF at room temperature for 15 h gave naphthoxazine derivatives 50 in 90–95% yields, which were reduced with sodium boronhydride in THF/HOAc at room temperature to afford the target chiral aminonaphthol ligands 51 in 78–87% yields (Scheme 15). The results of asymmetric phenyl transfer to aromatic aldehydes catalyzed by these chiral ligands 51 indicated that enantioselectivities were greatly influenced by the electronic and steric effects of the ligands.^40^

**Scheme 16:**
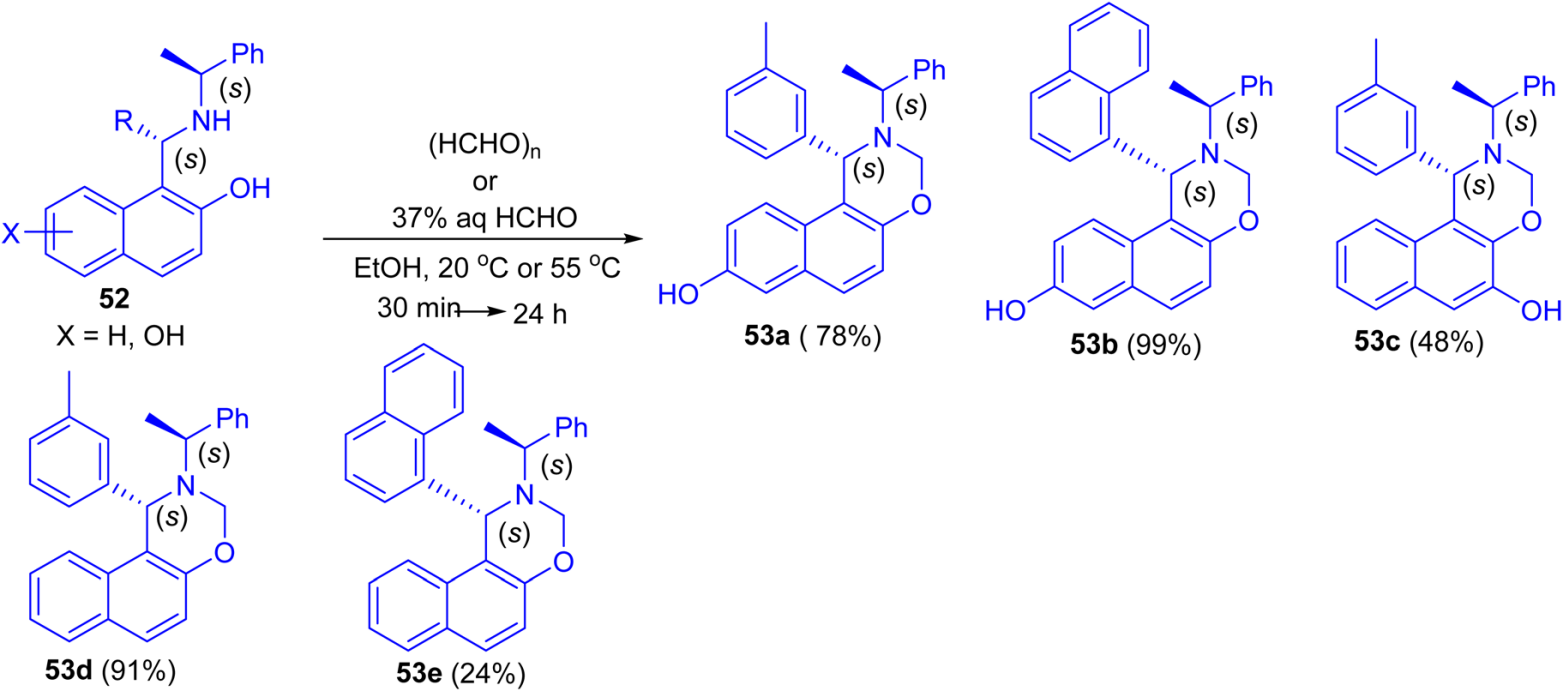
Synthesis of 1,3-oxazines 53a–e.

In 2013, Dimitrov and co-workers have demonstrated Betti base derivatives 52 were easily transformed into the corresponding 1,3-oxazines 53a–e in 24–99% yields by reacting them with formaldehyde (in the form of paraformaldehyde or formalin) in EtOH at 20 °C or 55 °C for 30 min to 24 h (Scheme 16).^41^

**Scheme 17:**
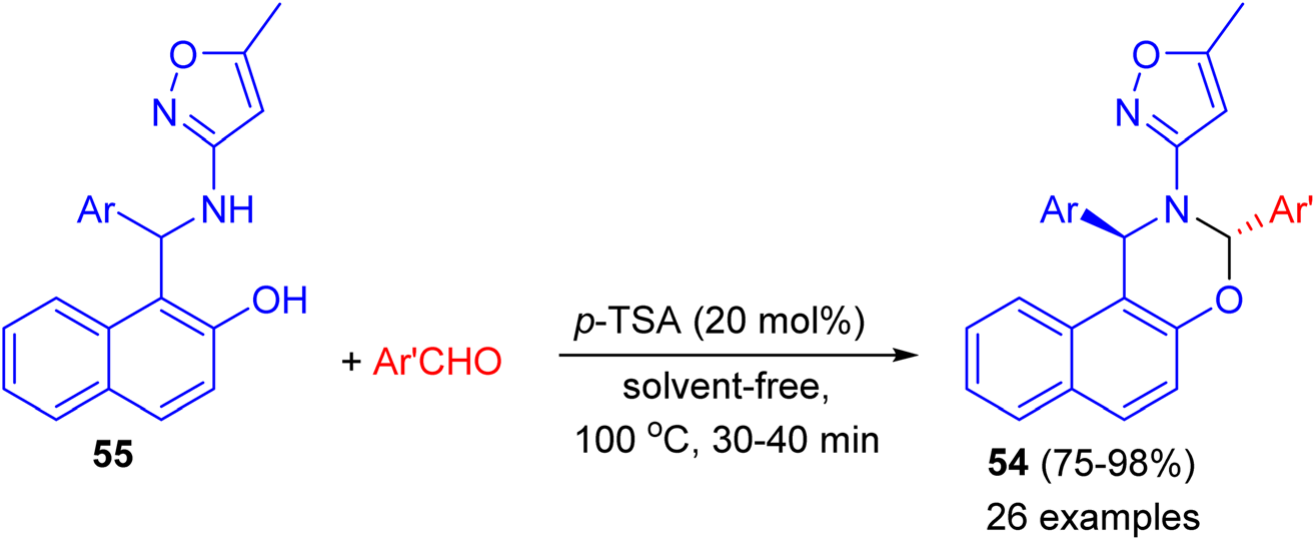
Diastereoselective synthesis of *trans*-3-(5-methylisoxazol-3-yl)-3,4-dihydro-2*H*-naphtho[2,3-*e*][1,3]oxazines 54.

In 2014, Khosropour and his group described stereoselective synthesis of *trans*-3-(5-methylisoxazol-3-yl)-3,4-dihydro-2*H*-naphtho[2,3-*e*][1,3]oxazine derivatives 54 in high yields (75–98%) by the reaction of 1-(aryl (5-methyl-isoxazol-3-ylamino)methyl)naphthalen-2-ols 55 with aromatic aldehydes using *p*-TSA as catalyst under solvent-free conditions at 100 °C for 30–40 min. This reaction includes some important aspects like straightforward operation, easy workup procedure and absence of transition metal catalysts. In the proposed mechanism, first, the Betti base attacks aryl aldehyde in the presence of the catalyst to generate the corresponding imine as the pivotal intermediate. Finally, the intermediate undergoes 6-*endo*-dig to give the corresponding [1,3]oxazine 54 as illustrated in Scheme 17.^42^

**Scheme 18:**
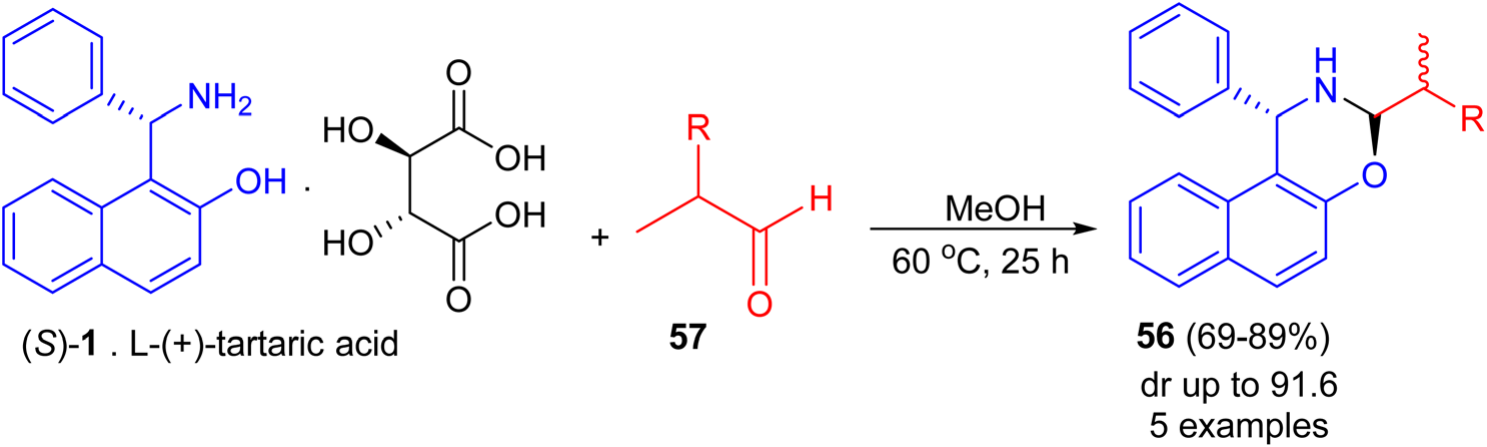
Preparation of (*S*)-naphthoxazines 56.

Next, Srimannarayana *et al.* reported preparation of (*S*)-naphthoxazines 56 with diastereoisomeric ratios (dr) in almost equal in 69–89% yields by the treatment of l-(+)-tartaric acid salt of the (*S*)-enantiomer 1 with various racemic α-alkyl dihydrocinnamic aldehydes (2-alkyl-3-phenylpropanals) 57 in MeOH at 60 °C for 25 h (Scheme 18).^43^

**Scheme 19:**
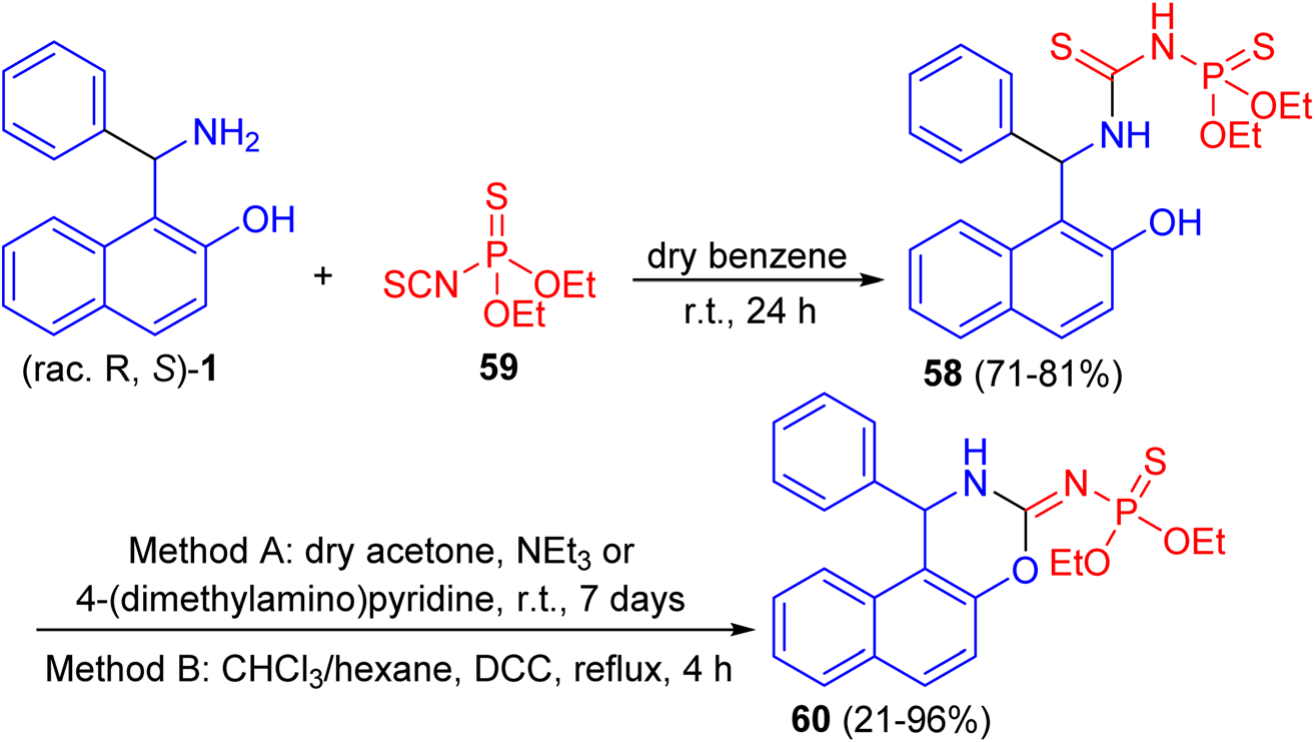
Synthesis of thiophosphorylated oxazines 60.

Further, a number of chiral racemic and enanthiopure thiophosphorylated thioureas 58 synthesized by the reaction of 1-(α-aminobenzyl)-2-naphthol 1 with *O*,*O*-diethyl thiophosphoryl isothiocyanate 59 in dry benzene at room temperature for 24 h. It was found that such thioureas undergo the cyclization reaction under basic conditions with hydrogen sulfide elimination *via* two methods (method A: in dry acetone, triethylamine or 4-(dimethylamino)pyridine at room temperature for 7 days; method B: in chloroform and hexane, *N*,*N*′-dicyclohexylcarbodiimide at reflux for 4 h) and afforded thiophosphorylated oxasines 60 as depicted in Scheme 19.^44^

**Scheme 20:**
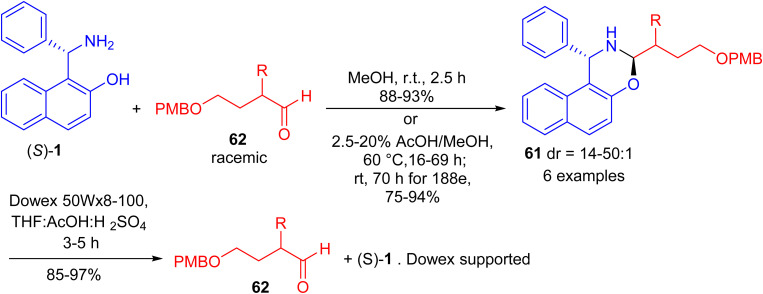
Synthesis of naphthoxazines 61.

In 2017, Paolucci and co-workers accomplished synthesis of naphthoxazines 61 in 75–94% yields by the reaction of α-epimerizable 4-hydroxybutyraldeydes 62 with (*S*)-Betti base 1 in MeOH at room temperature for 2 h or 2.5–20% AcOH in MeOH at 60 °C for 16–69 h. The diastereoisomeric naphthoxazines 61 underwent hydrolysis by using pre-washed resin 20 in a mixture of aq. H_2_SO_4_, THF and acetic acid within 3–5 hours, and the aldehydes are obtained with higher enantiomeric enrichment (Scheme 20).^45^

**Scheme 21:**
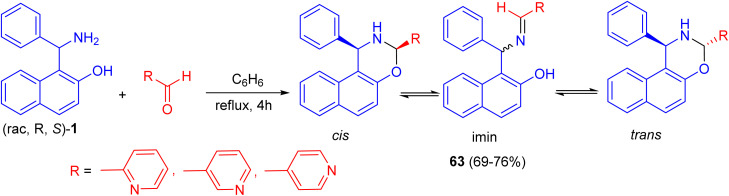
Synthesis of oxazines 63.

In 2018, Alfonsov *et al.* developed a method to synthesize oxazines 63 in 69–76% yields by the reaction of the racemic Betti base 1 with 2-, 3-, and 4-pyridinecarbaldehydes in benzene under reflux for 4 h. In the solution, a three-component ring-chain equilibrium is established between the imine form and *cis*- and *trans*-oxazines. It should be noted that the content of the imine form (CDCl_3_) in a series of compounds 63 is significantly lower compared with 1,3-diphenylnaphthoxazine. *Trans* form is predominant in all cases (Scheme 21). It can be assumed that the imine/oxazine ratio is mainly influenced by electronic interactions, while the *cis*-/*trans*-1,3-oxazine ratio is more influenced by steric factors.^46^

**Scheme 22:**
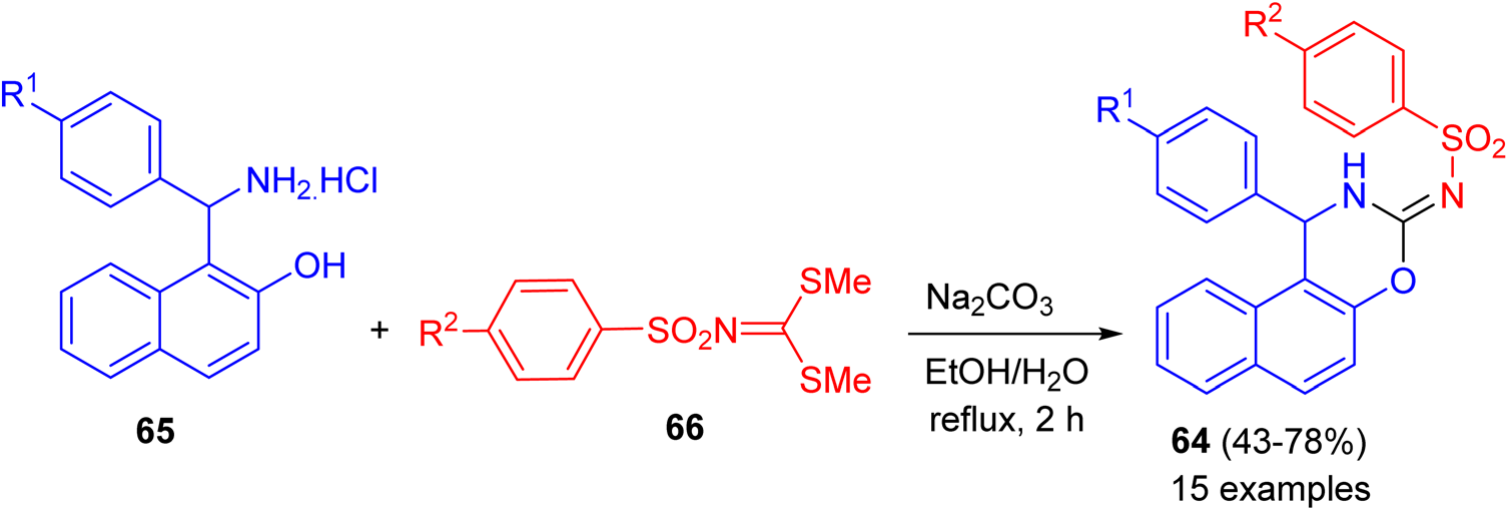
Synthesis of naphtho[1,2-*e*][1,3]oxazines 64.

In 2020, a series of naphtho[1,2-*e*][1,3]oxazines 64 in 43–78% yields bearing arylsulfonamide moiety synthesized *via* a one-pot reactions of 1-(amino(aryl)methyl)naphthalen-2-ol hydrochloride 65, dimethyl arylsulfonylcarbonimido dithioate 66 using Na_2_CO_3_ in H_2_O/EtOH (1 : 3) under reflux conditions for 2 h (Scheme 22). All of the compounds examined for their *in vitro* anticancer activity against breast (MCF-7), colon (HCT116), and B-CLL (Waco3-CD5) cancers. Some of these compounds showed remarkable activities against MCF-7 (breast) and HCT116 (colon) cancers with comparable IC_50_ (the half maximal inhibitory concentration) values as that of known drugs such as 5-fluorouracil (5-FU). *In vitro* antimicrobial activities of all compounds were also evaluated against five human pathogenic fungi strains and two bacteria (one Gram positive and one Gram negative). The best MICs (Minimum Inhibitory Concentrations) were found against the *C. albicans*.^47^

## 3 Synthesis of bis-naphthoxazines

**Scheme 23:**
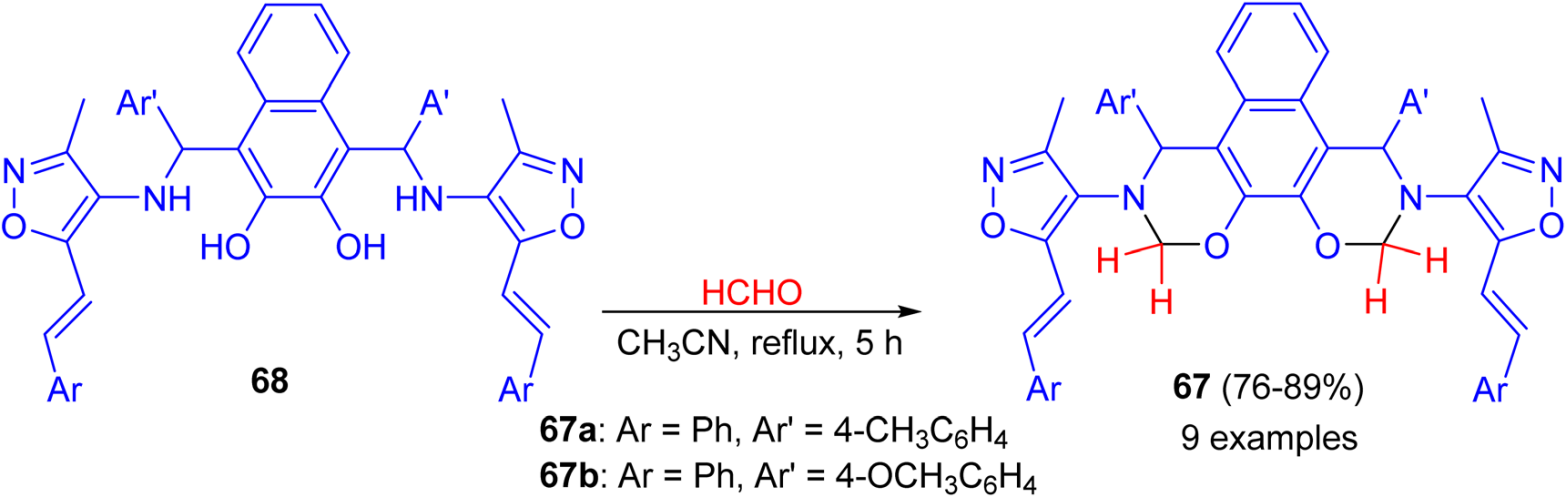
Synthesis of bis-(isoxazolyl-1,3-oxazine)naphthalenes 67.

In 2014, synthesis of bis-(isoxazolyl-1,3-oxazine)naphthalenes 67 in 76–89% yields accomplished by the reaction of Betti bases 68 with formaldehyde (37%) in acetonitrile under reflux conditions for 5 h. The synthesized compounds were evaluated for *in vitro* antimicrobial activity. Compounds 67a and 67b were proved to possess remarkable antimicrobial activity (Scheme 23).^48^

**Scheme 24:**
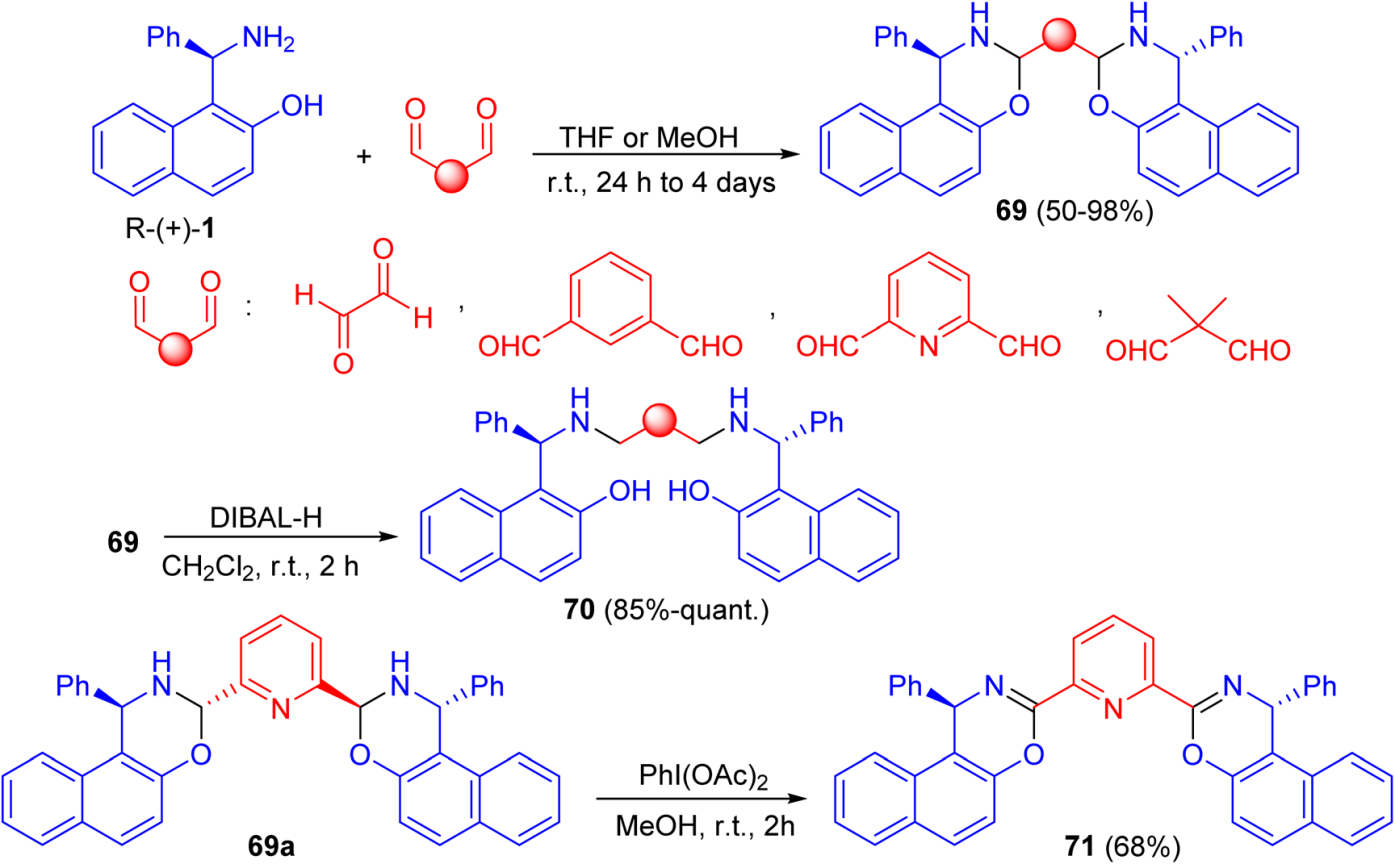
Synthesis of compounds 69–71.

In 2016, Paolucci *et al.* described the synthesis of *trans*, *trans*-bisdihydrooxazinic compounds 69 in 50–98% yields from (*R*)-Betti base 1 and various dialdehydes in THF or MeOH at room temperature for 24 h to 4 days. Reduction of 69 with diisobutylaluminium hydride (DIBAL-H) in CH_2_Cl_2_ at room temperature for 2 h gave the corresponding compound 70 in very good yields. The oxidation of compound 69a with PhI(OAc)_2_ in MeOH for 2 h afforded bisoxazinic compound 71 in 68% yield as shown in Scheme 24. Two Cu complexes and one Sc complex were prepared from compound 71. The metal complexes were employed in some reactions to test their ability to promote the transformation and the asymmetric induction.^49^

## 4 Synthesis of naphthopyrrolooxazines, naphthopyridooxazines and naphthooxazinoazepines

**Scheme 25:**
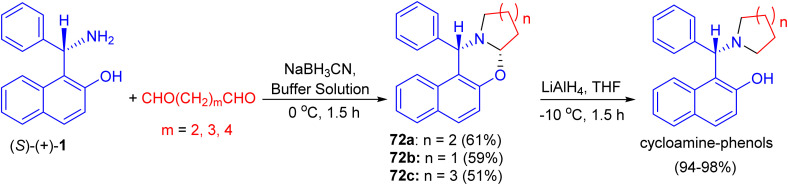
Synthesis of oxazine derivatives 72.

In 2002, Hu *et al.* synthesized unsubstituted piperido[2,1-*b*]oxazine derivative 72a in 61% yield by the reaction of (*S*)-1 with 1,5-pentanedial in the presence of NaBH_3_CN in a buffer solution (aqueous EtOH solution of Na_2_HPO_4_–KH_2_PO_4_) at 0 °C for 1.5 h. Similarly, 72b (59%) and 72c (51%) were obtained smoothly by using 1,4-butanedial and 1,6-hexanedial, respectively. When compounds 72 were treated with LiAlH_4_, the CO bond was cleaved selectively to yield the desired cycloamine-phenols without any loss of enantiomeric excess at −10 °C in 1.5 h. The asymmetric addition of ZnEt_2_ to benzaldehyde was tested in toluene with 10 mol% of cycloamine-phenol ligands to give the products in 93–96% yields and 73–99% ee (Scheme 25).^50^

**Scheme 26:**
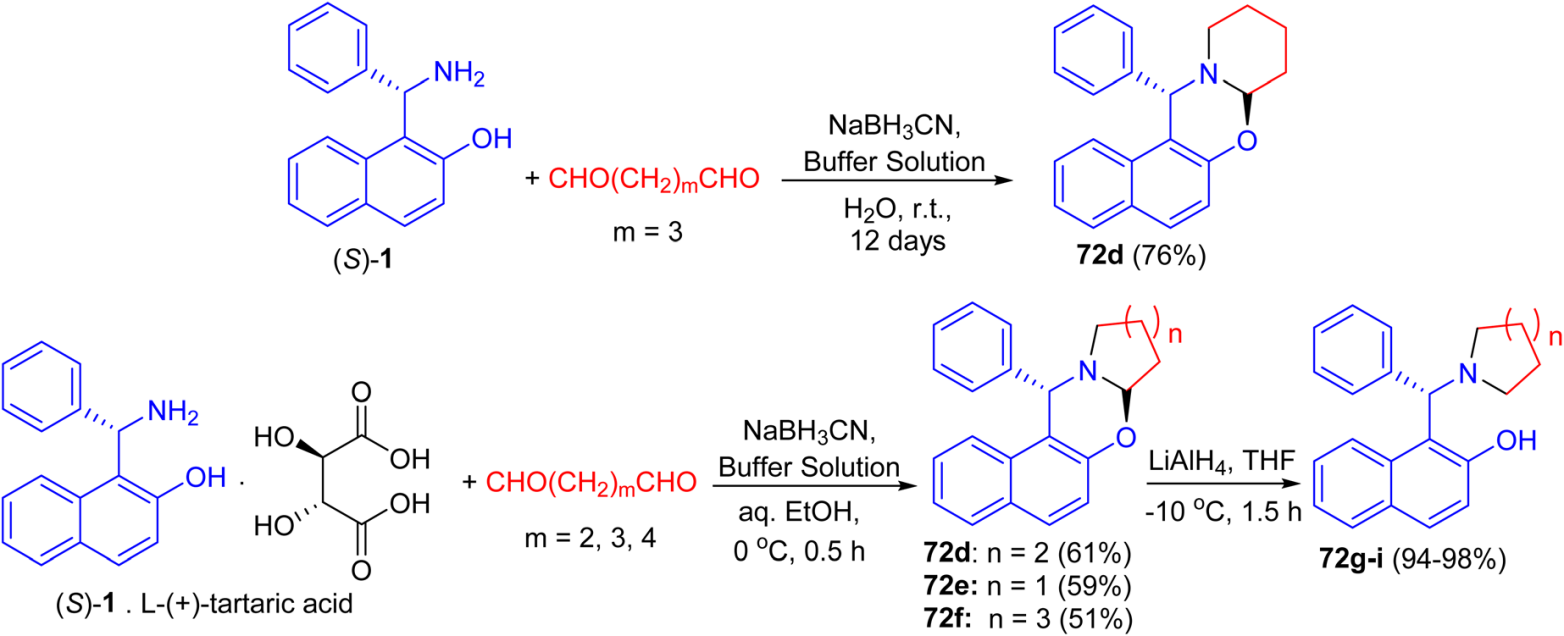
Synthesis of tetrahydropyrido[2,1-*b*]oxazines 72d–f.

After that, unsubstituted tetrahydropyrido-[2,1-*b*]oxazine 72d was obtained in 76% yield *via* the reaction of (*S*)-1 with pentane-1,5-dial in the presence of NaBH_3_CN in an aqueous buffer solution (Na_2_HPO_4_–KH_2_PO_4_) at room temperature for 12 days. Also, the salt of (*S*)-1 with l-(+)-tartaric acid, which is a precursor of (*S*)-1 in its optical resolution, was used directly as the starting material and 72d was obtained in 61% yield within 30 min in 50% aqueous EtOH. Similarly, 72e (59%) and 72f (51%) were prepared respectively by the replacement of pentane-1,5-dial with butane-1,4-dial and hexane-1,6-dial under the same conditions. These compounds converted to non-racemic 1-[-(1-azacycloalkyl)benzyl]-2-naphthols 72g–i by the selective cleavage of a C–O bond with LiAlH_4_ in THF at −10 °C for 1.5 h. The ligands with pyrrolidine and piperidine lead to highly efficient asymmetric induction in the addition of diethylzinc to aryl aldehydes with up to 96% yield and 99% ee (Scheme 26).^51^

**Scheme 27:**
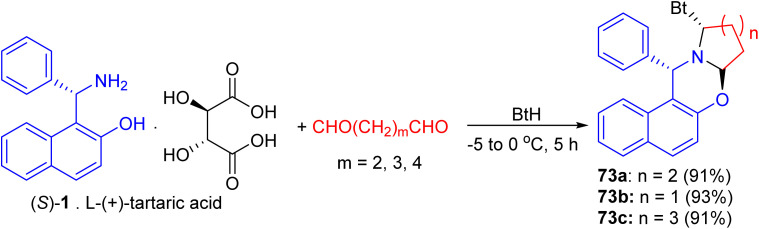
Preparation of α-benzotriazolyl 1-piperido[2,1-*b*][1,3]-oxazine 73a–c.

In 2004, following Katritzky's procedure, a mixture of *S*-1 [as a salt of l-(+)-tartaric acid], pentane-1,5-dial and BtH in CH_2_Cl_2_ was stirred at 0 °C for 5 hours. As expected, the diastereopure α-benzotriazolyl-piperido[2,1-*b*][1,3]-oxazine 73a was obtained in 91% yield (Scheme 2). Similarly, replacement of pentane-1,5-dial by butane-1,4-dial and hexane-1,6-dial, the five- and seven membered azacyclic analogues 73b and 73c were obtained respectively in high yields (93% and 91%) and diastereoselectivities. Moreover, both C–Bt and C–O bonds in the oxazines 73 were cut clearly *via* LiAlH_4_ within half hour at 0 °C to afford chiral Betti bases in high yields, which have been proved to be excellent chiral ligands in the asymmetric addition of ZnEt_2_ to aldehydes (Scheme 27).^52^

**Scheme 28:**
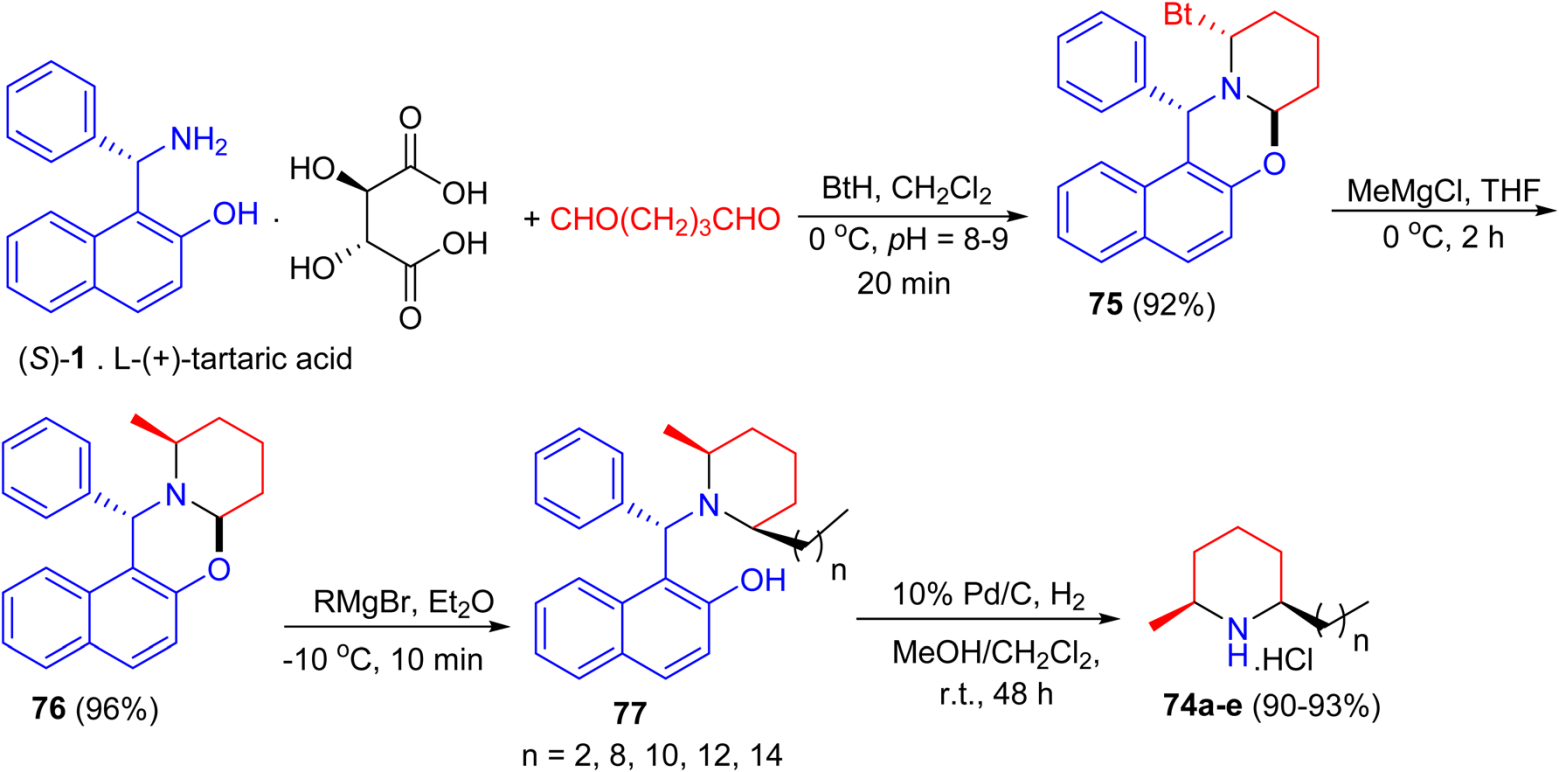
Total syntheses of enantiopure (2*S*,6*R*)-dihydropinidine and (2*S*,6*R*)-isosolenopsins 74.

In 2005, total syntheses of enantiopure alkaloidal natural products (2*S*,6*R*)-dihydropinidine (74a, as hydrochloride) and (2*S*,6*R*)-isosolenopsins (74b–e, as hydrochlorides) achieved with the shortest steps and unprecedented high total yields by using a strategy of the formation-cleavage of 1,3-oxazinane. First, Betti base (*S*)-1 [as a salt of l-(+)-tartaric acid] condensed with pentane-1,5-dial and benzotriazole to diastereopurely yield 75 in 92% yield. By using THF as solvent, a solvent controlled monoalkylation of 75 was achieved to give diastereopure 76 in 96% yield. Then 76 was alkylated with the corresponding Grignard reagent followed by *N*-debenzylation straightforward to amine hydrochloride by Pd/C-catalyzed hydrogenolysis in the presence of CH_2_Cl_2_ to yield target products 74a–e in 90–93% yields (two steps), respectively (Scheme 28).^53^

**Scheme 29:**
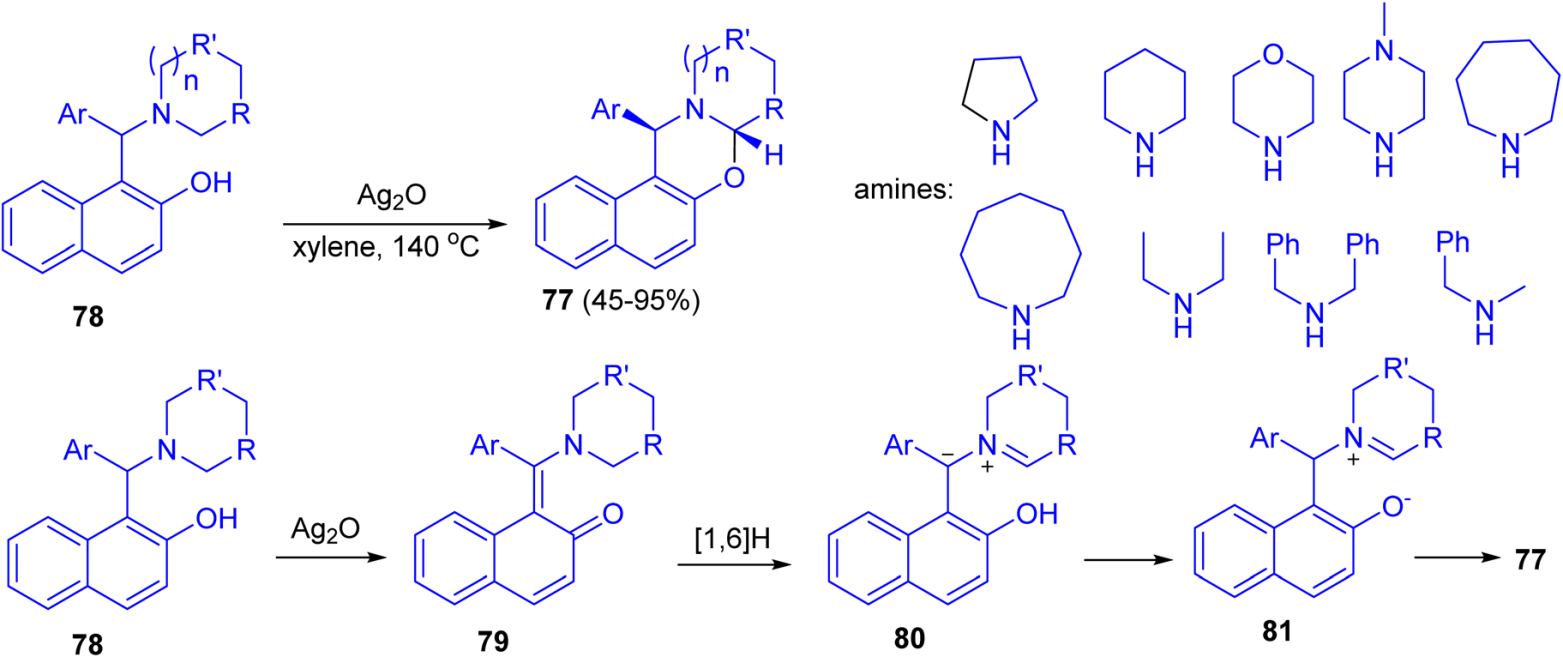
Synthesis of ring fused oxazines 77.

In 2014, Jana and co-workers reported diastereoselective α-C–H functionalization of aliphatic N-heterocycles for the synthesis of ring fused oxazines 77. The reaction of Betti base derivatives 78 with Ag_2_O in xylene at 140 °C afforded the desired product 77 in 45–95% yields. A probable mechanism is depicted in Scheme 29. First, Betti base 78 reacted with Ag_2_O to provide the corresponding *o*-quinone methide 79. Thus a 1,6-H (alpha to the nitrogen) transfer can be operative on 79 to furnish the zwitterionic intermediate 80. Alternatively, 80 can also be formed *via* mesomerization of quinone methide 79 followed by a proton transfer. Protonation of 80 and subsequent diastereoselective cyclization of the resulting iminium ion 81 gave rise to *trans*-oxazine 77. The electron-withdrawing group on Ar and the electron donating nature of R will stabilize the zwitterionic intermediate 80. The expected lower yields for morpholine derivatives are due to the reduced stability of the zwitterionic intermediate 80 because of the negative inductive effect of the ring oxygen atom.^54^

**Scheme 30:**
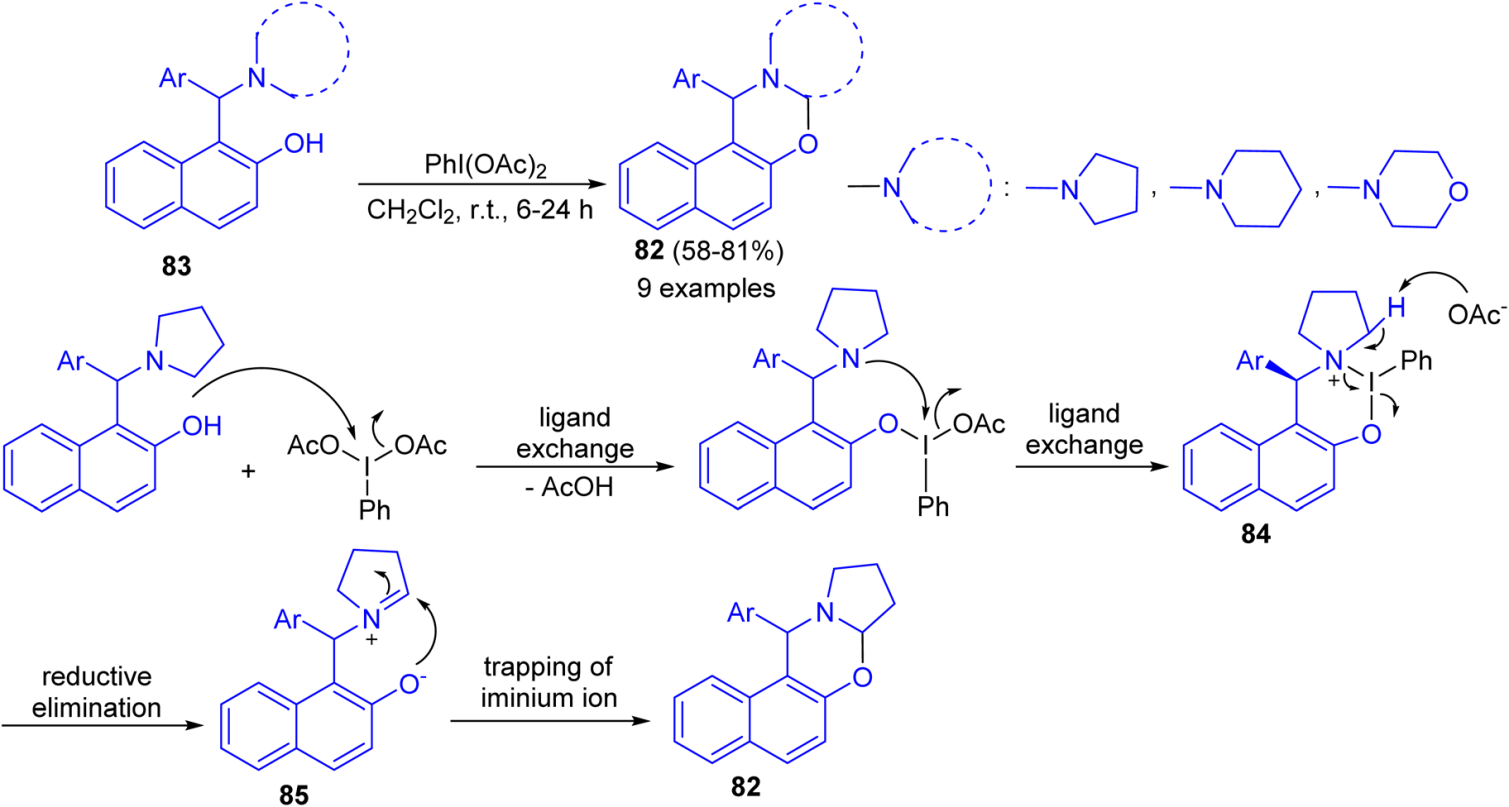
Synthesis of 1,3-naphthoxazines 82.

In 2016, Karade *et al.* synthesized a series of 1,3-napthoxazines 82 in 58–81% yields by the reactions of 1-(α-aminoalkyl)-2-naphthols 83 with (diacetoxyiodo)benzene (DIB) in CH_2_Cl_2_ at room temperature for 6–24 h. This synthesis of 1,3-naphthoxazine involves transition metal-free cross-dehydrogenative C–O bond formation at sp^3^ C–H bond adjacent to tertiary nitrogen. The tentative mechanism is shown in Scheme 30. The reaction of DIB with phenolic substrate 83 can inhibit the oxidative dearomatization due to the formation of putative six membered iodine(iii) heterocycle 84. The propensity of 84 for reductive elimination of iodobenzene will generate the requisite iminium ion 85 which will be intramolecularly trapped by phenoxide anion to form *trans*-1,3-naphthoxazine 82.^55^

**Scheme 31:**
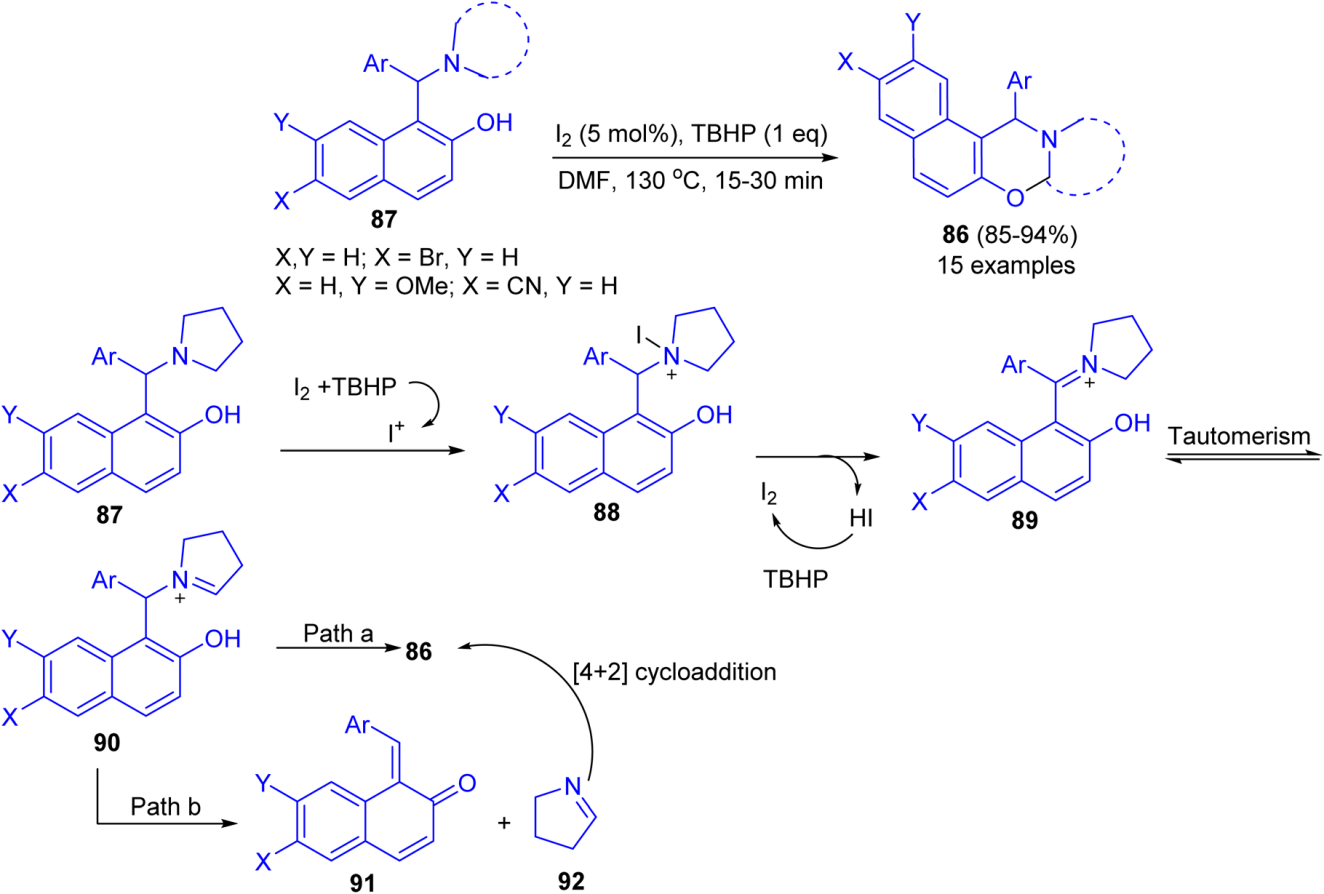
Intramolecular CDC of Betti base leading to 1,3-oxazines 86.

Deb and co-workers developed an iodine-*tert*-butylhydroperoxide for the synthesis of 1,3-oxazines 86 in 85–94% yields from Betti bases 87 under heating at 130 °C in DMF as solvent within 15–30 min. The method uses inexpensive and nonhazardous I_2_ catalyst and TBHP as the oxidant. Moreover, the reaction could easily be scaled up to multigram scale with excellent yield, in which the product can be successfully isolated by recrystallization. The proposed a tentative mechanism for the reaction is outlined in Scheme 31. First, 87 is converted to iodopyrrolidinium 88 in the presence of iodine source. The intermediate 88 then releases a molecule of HI to form iminium ion 89, which undergoes tautomerisation to iminium ion 90. Next, 90 undergoes cyclization by two different pathways. In path a, 90 directly cyclizes to the final product 86. On the other hand, in path b, 90 first undergoes fragmentation to 91 and 92. Subsequently 91 and 92 coupled through the [4 + 2]-cycloaddition reaction to form 86.^56^

**Scheme 32:**
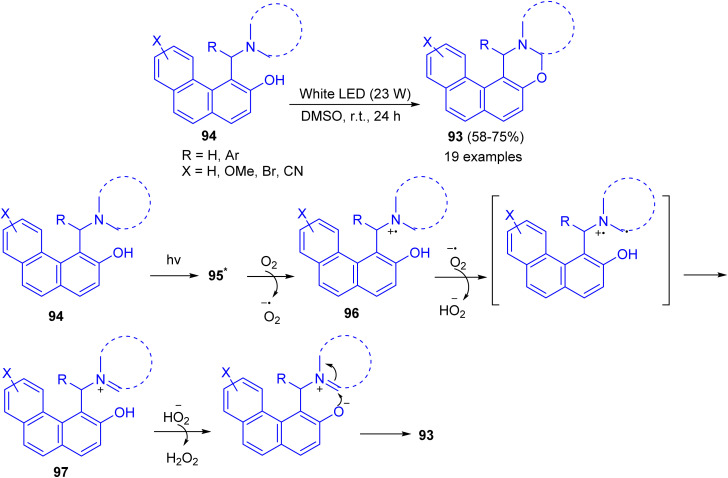
Preparation of 1,3-oxazines 93.

Recently, a catalyst-free cross-dehydrogenative coupling reaction accomplished by visible light for the synthesis of 1,3-oxazines 93. When α-aminoalkylnaphthols 94 are irradiated with white light-emitting diode (LED, 23 W) in dimethyl sulfoxide as solvent in an open reaction vessel at room temperature for 24 h, cyclization takes place *via* α-C–H activation of tertiary amine moiety and forms a new C–O bond. Biologically important 1,3-oxazines are obtained in 58–85% yields. The various advantages of this methodology are (i) the reaction is catalyst-free, (ii) only aerial oxygen is needed as the oxidant and (iii) the reaction is visible light-promoted. A mechanism is proposed in Scheme 32. The Betti base 94 is first excited by visible light to 95 which reacts with O_2_ and transfer a single electron to O_2_. This generates a radical cation 96 and a superoxide. The superoxide then abstracts a hydrogen atom from 96 which forms the iminium ion 97 and hydroperoxide anion. The formation of iminium ion proves that C–H activation takes place rather than O–H activation, though in some cases O–H activation may prefer. The hydroperoxide anion then removes a proton from –OH group of 97 followed by cyclization with the elimination of a molecule of hydrogen peroxide.^57^

## 5 Synthesis of naphthoxazinobenzoxazines

**Scheme 33:**
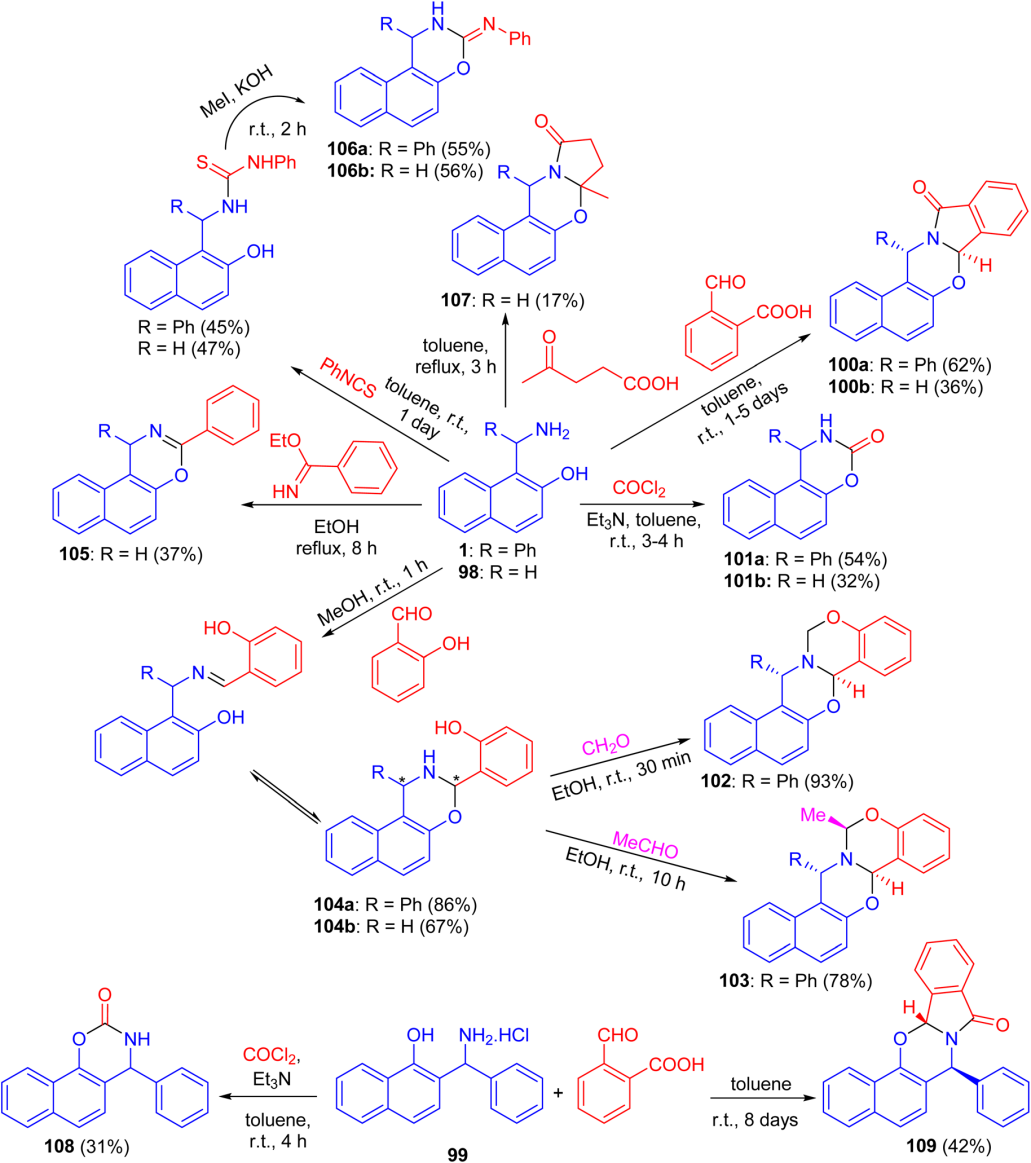
Synthesis of naphthoxazine derivatives 100–109.

In 2004, Fulop *et al.* described domino ring-closure reactions of 1-(α-aminobenzyl)-2-naphthol (1), 1-aminomethyl-2-naphthol (98) and 2-(α-aminobenzyl)-1-naphthol (reverse Betti base: 99) with phosgene, ethylbenzimidate, phenylisocyanate/MeI, 2-carboxybenzaldehyde, levulinic acid, salicylaldehyde/formalin or salicylaldehyde/acetaldehyde which afforded naphth[1,2-*e*][1,3]oxazine and naphth[2,1-*e*][1,3]oxazine derivatives 100–109 in 17–93% yields (Scheme 33).^58^

**Scheme 34:**
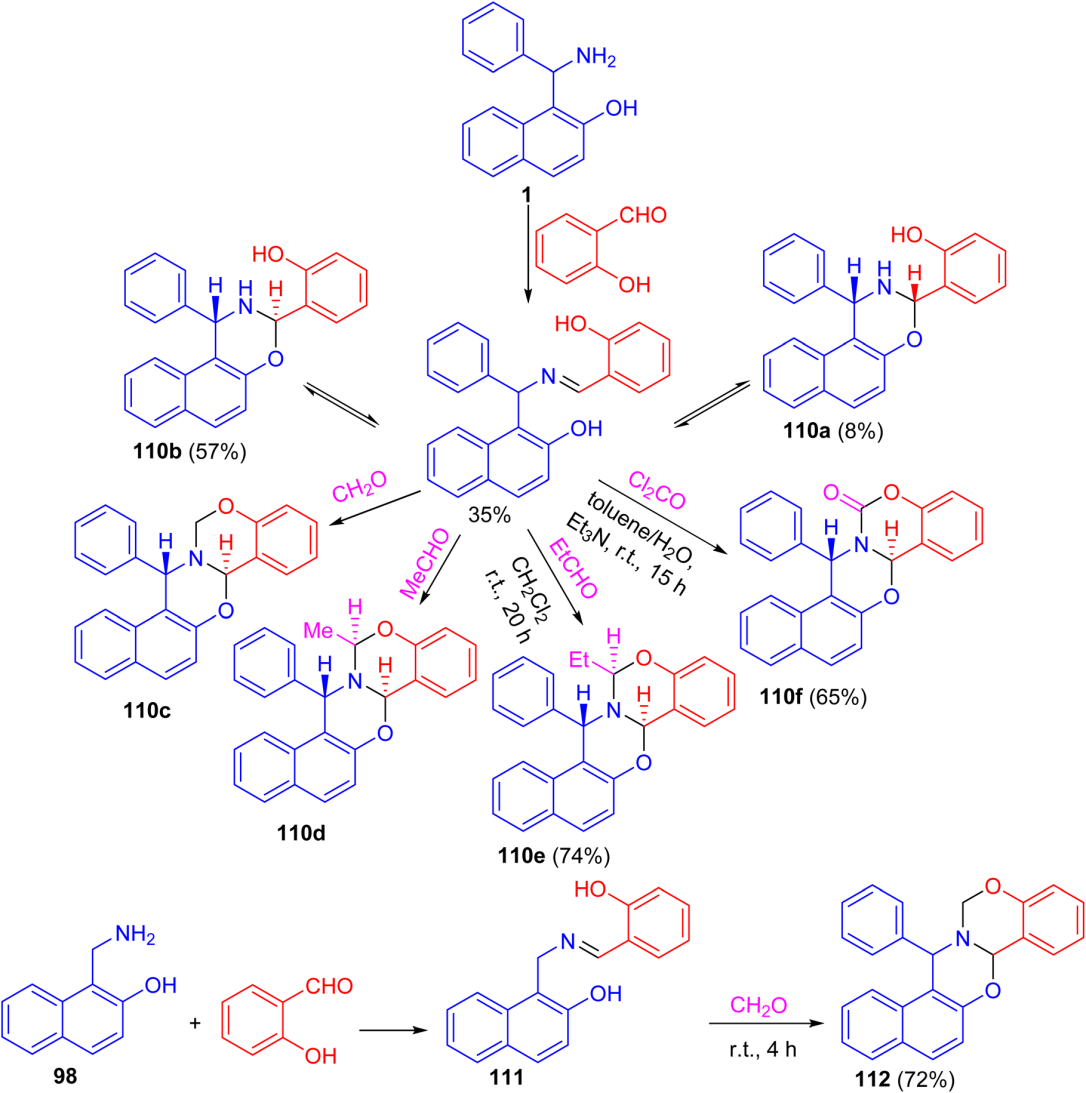
Synthesis of naphth[1′2′:5,6][1,3]oxazino[3,2-*c*][1,3]benzoxazines 110 and 112.

After that, a functional group, the hydroxy group, was inserted into a Betti base 1 by reaction with salicylaldehyde, and the naphthoxazine derivatives thus obtained were converted by ring-closure reactions with formaldehyde, acetaldehyde, propionaldehyde or phosgene to the corresponding naphth[1′2′:5,6][1,3]oxazino[3,2-*c*][1,3]benzoxazine derivatives 110. Moreover, condensation of 1-aminomethyl-2-naphthol 98 with salicylaldehyde led to the Schiff base 111, which could be easily converted into the unsubstituted naphth[1′,2′:5,6][1,3]-oxazino[3,2-*c*][1,3]benzoxazine derivative 112 in 72% yield. Further, by NMR spectroscopy and an accompanying molecular modelling, both quantitative anisotropic ring current effects of the aromatic moieties and steric substituent effects were employed to determine the stereochemistry of the naphthoxazinobenzoxazine derivatives (Scheme 34).^59^

## 6 Synthesis of naphthoxazinoisoquinolines/naphthoxazinoquinolines

**Scheme 35:**
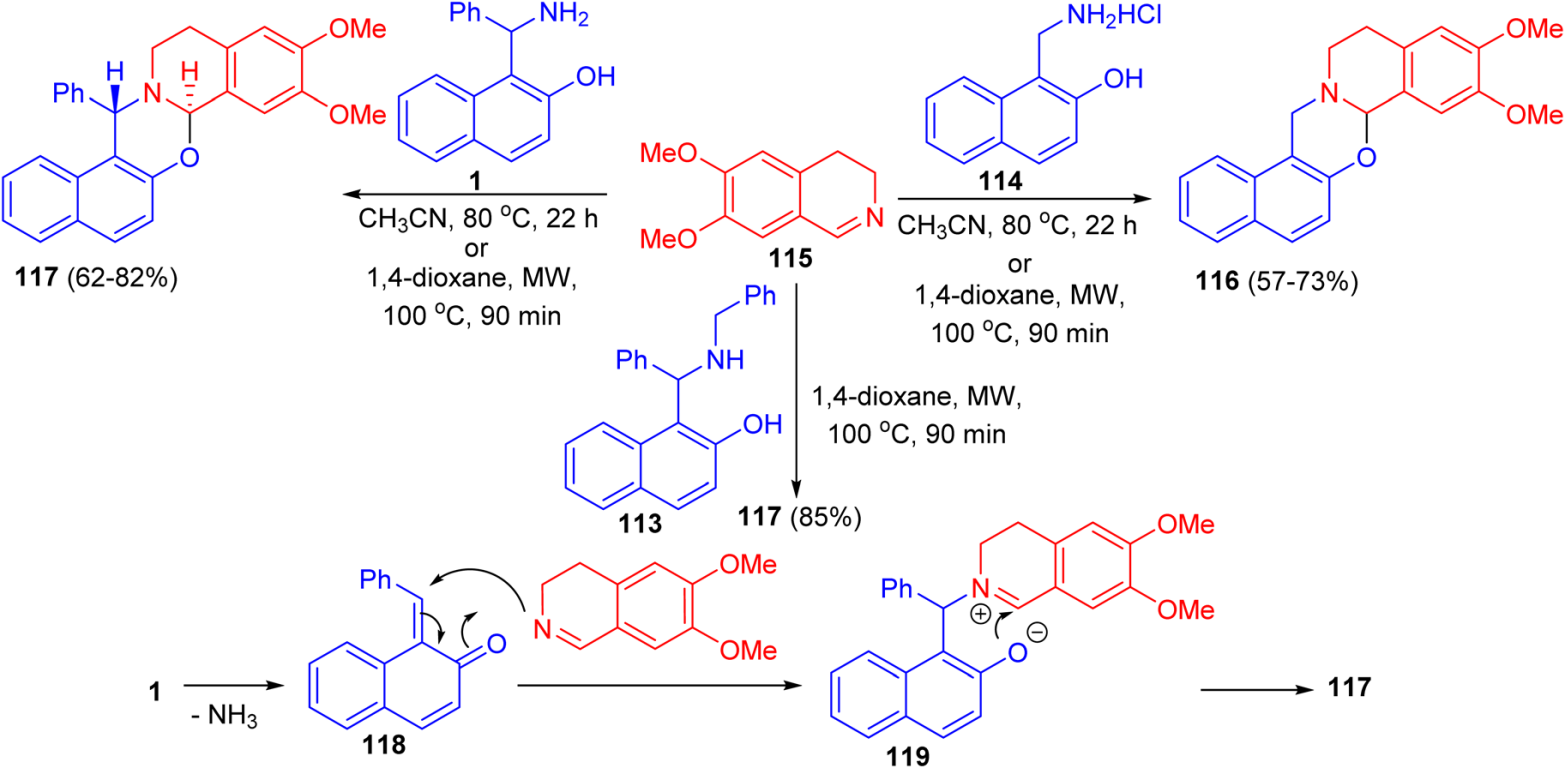
Synthesis of pentacyclic oxazinoisoquinolines 116 and 117.

In 2011, unexpected reactions between 1-α-aminobenzyl-2-naphthol (1), *N*-benzyl-1-α-aminobenzyl-2-naphthol (113), 1-aminomethyl-2-naphthol hydrochloride (114) and 6,7-dimethoxy-3,4-dihydroisoquinoline (115) to furnish naphth[1,2-*e*][1,3]oxazino[2,3-*a*]isoquinolines 116 and 117 were reported. The reaction conditions involved classical heating at 80 °C in MeCN for 22 h, or the use of microwave conditions (100 °C), which allowed a reduction of the reaction time to 90 min and resulted in somewhat higher yields of the product. The proposed reaction pathway for the formation of 117 is depicted in Scheme 35. The first step is the loss of ammonia, leading to 118. The next step involves nucleophilic attack of the dihydroisoquinoline nitrogen on the C=C bond, forming 119. The driving force for this step is aromatization. The ionic intermediate 119 is stabilized by attack of the phenolic ion on the imine carbon of the dihydroisoquinoline, leading to 117.^60^

**Scheme 36:**
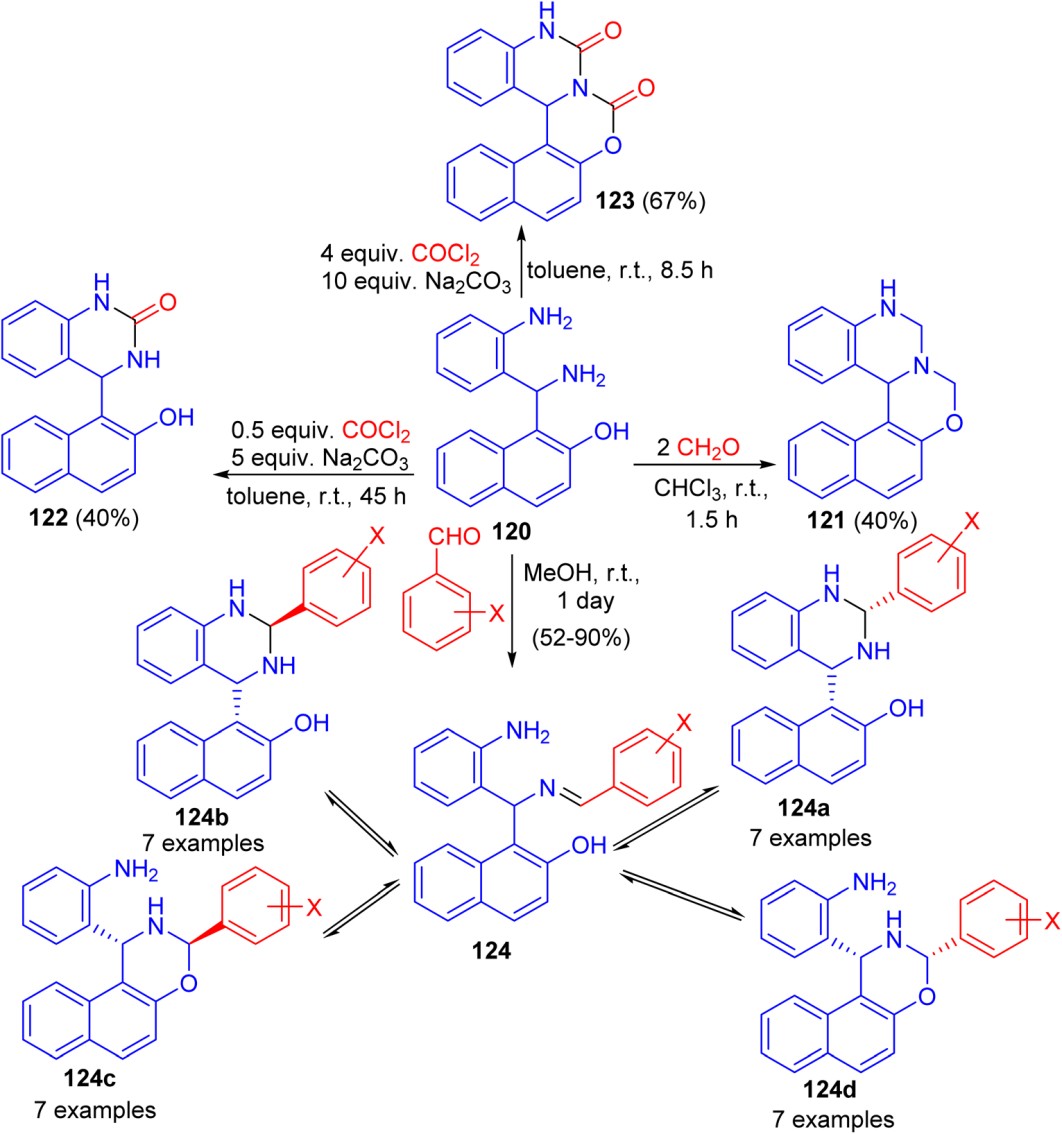
Synthesis of naphth[1,2-*e*][1,3]oxazino[3,4-*c*]quinazoline derivatives 121–124.

Next, Folup and co-workers described a highly functionalized aminonaphthol derivative 120 converted to the corresponding naphth[1,2-*e*][1,3]oxazino[3,4-*c*]quinazoline derivatives 121–124 in ring-closure reactions with formaldehyde in CHCl_3_, benzaldehyde in MeOH and/or phosgene in toluene at room temperature. The products obtained *via* the reactions of 120 with substituted benzaldehydes can potentially furnish five-component tautomeric mixtures 124 in CD_2_Cl_2_ at 300 K (Scheme 36).^61^

**Scheme 37:**
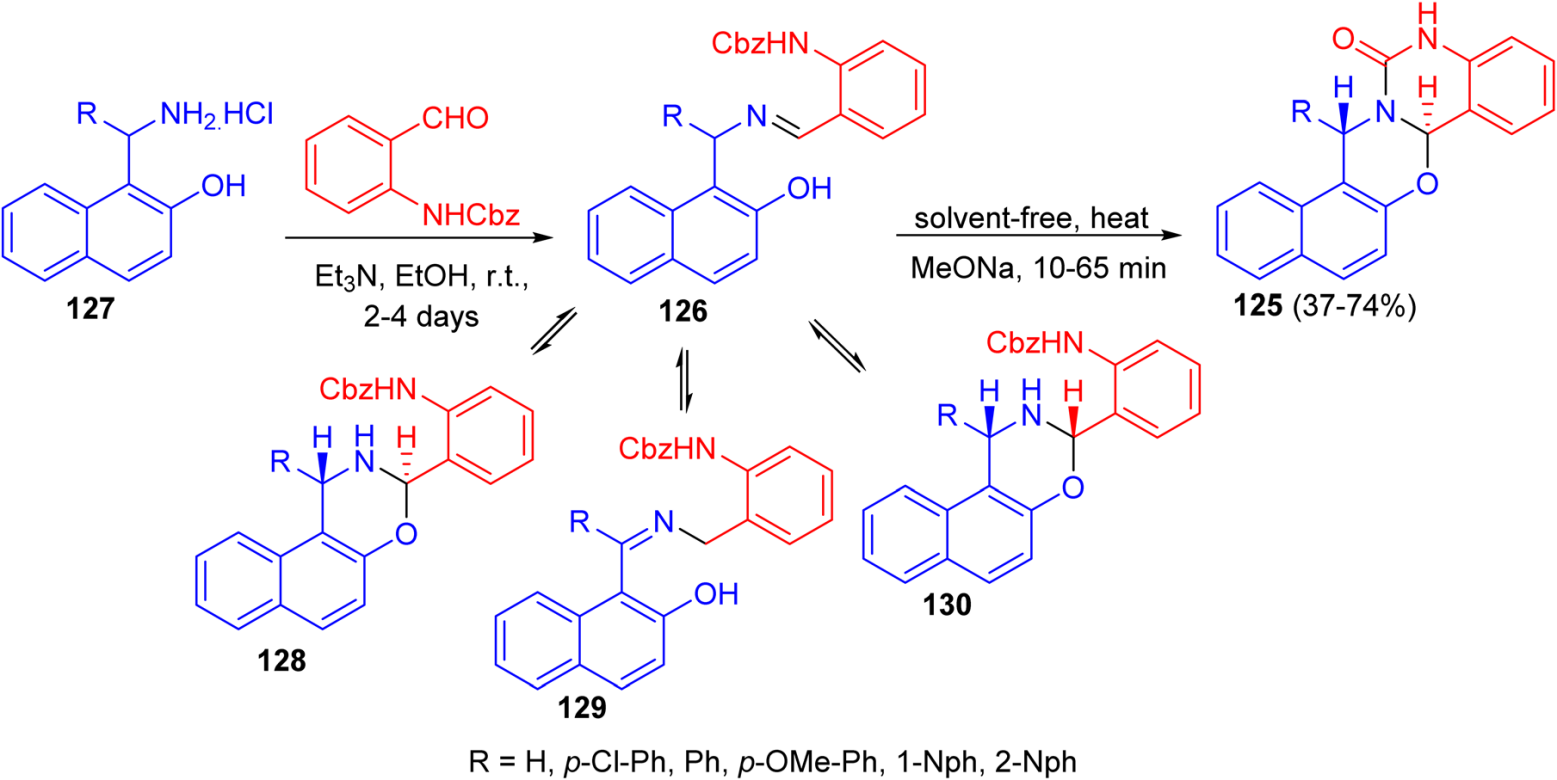
Synthesis of naphth[1,2-*e*][1,3]oxazino[3,2-*c*]quinazolin-13-one derivatives 125.

In 2012, the syntheses of naphth[1,2-*e*][1,3]oxazino[3,2-*c*]quinazolin-13-one derivatives 125 in 37–74% yields were reported by the solvent-free heating of benzyloxycarbonyl-protected intermediates 126 with MeONa for 10–65 min. For intermediates 126, prepared by the reactions of substituted aminonaphthols 127 with benzyl *N*-(2-formylphenyl)carbamate in the presence of Et_3_N in EtOH for 2–4 days at room temperature, not only the expected *trans* ring form 128 and chain form 126, but also the rearranged chain form 129 as a new tautomer were detected in DMSO at room temperature. The quantity of 130 in the tautomeric mixture was changed with time. Moreover, conformational analyses of the target heterocycles 125 by NMR spectroscopy and accompanying theoretical calculations at the DFT level of theory revealed that the oxazine ring preferred a twisted chair conformation and the quinazolone ring was planar. Besides the conformations, both the configurations at C-7a and C-15 and the preferred rotamers of the 1-naphthyl substituent at C-15 were assigned, which allowed evaluation of the aryl substituent-dependent steric hindrance in this part of the molecules (Scheme 37).^62^

**Scheme 38:**
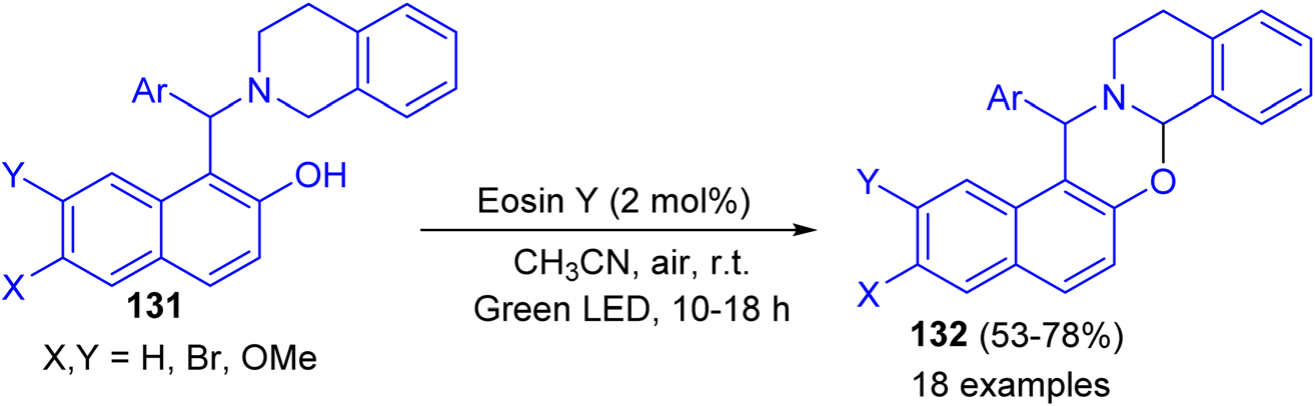
Visible light-promoted synthesis of 1,3-oxazines 132.

In 2017, Baruah and co-workers developed visible light intramolecular cross dehydrogenative coupling of 1-aminoalkyl-2-naphthols 131 to 1,3-oxazines 132 in 53–78% yields using green LED lamp as the light source and eosin Y functions as photoredox catalyst in CH_3_CN at room temperature for 10–18 h. Organic photoredox catalyst eosin Y is used which is very cheap and non-hazardous. Moreover, aerial oxygen is used as the oxidant. In the proposed mechanism, the reaction proceeds through the formation of iminium ion intermediate (Scheme 38).^63^

**Scheme 39:**
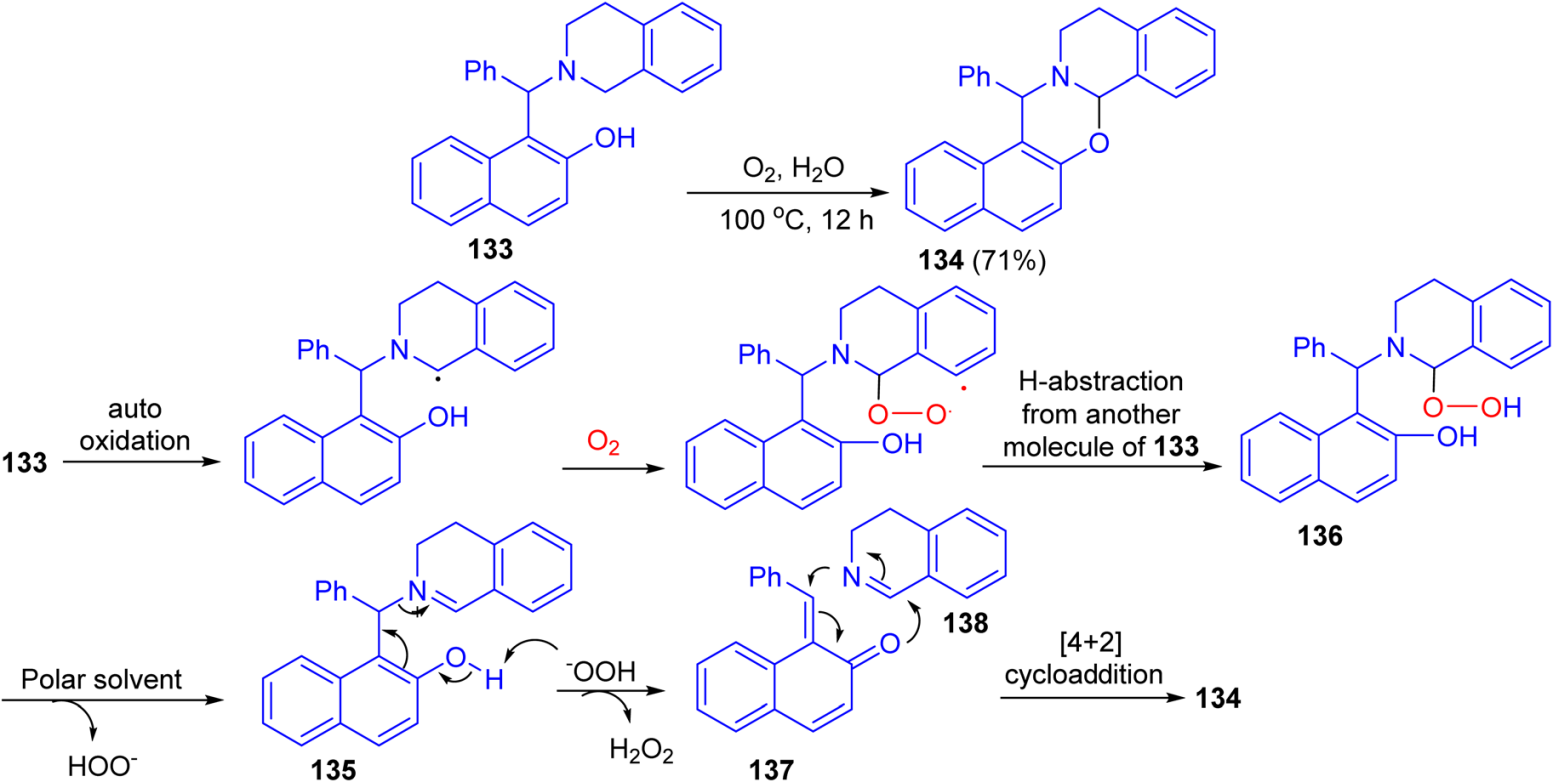
Synthesis of 1,3-oxazine 134.

After that, the reaction of Betti base 133 in H_2_O in the presence of O_2_ as the sole oxidant at 100 °C afforded 1,3-oxazine 134 in 71% yield after 12 h. The proposed mechanism is illustrated in Scheme 39. Compound 133 gets oxidized by oxygen to iminium ion 135. They believe that a polar solvent might help eliminate the –OOH group from 136 through solvation. The iminium ion 135 then undergoes fragmentation to quinone methide 137 and 3,4-dihydroisoquinoline 138. These two fragments are then coupled with each other through [4 + 2]-cycloaddition to form the product 134.^64^

## 7 The other fused-heterocycles

**Scheme 40:**
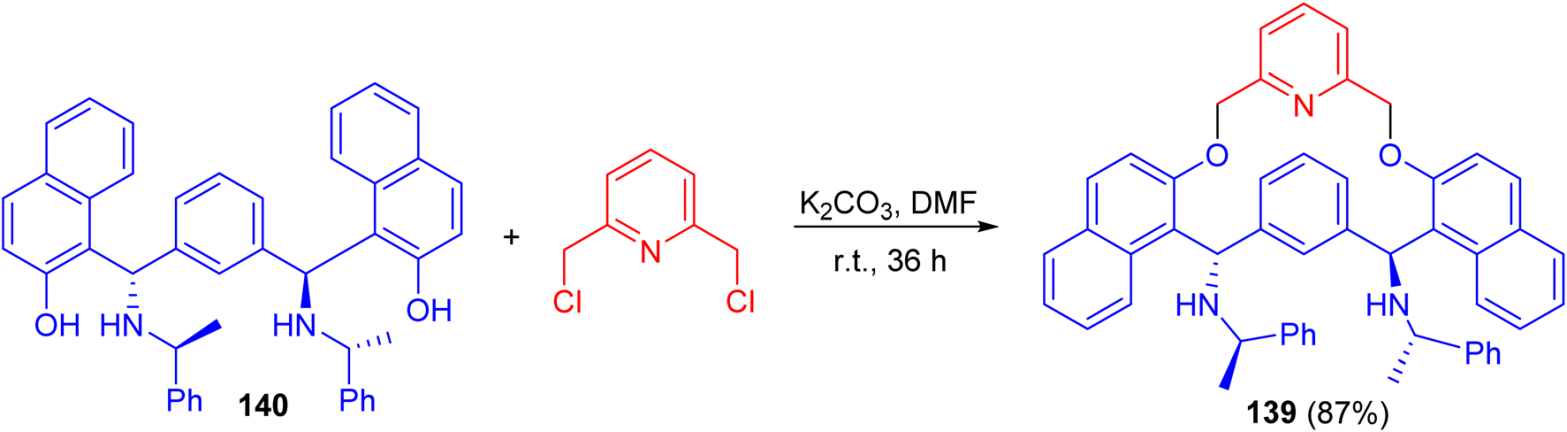
Synthesis of macrocyclic compound 139.

In 2007, Zhang and co-workers prepared macrocyclic compound 139 in 87% yield by the reaction of *C*_2_-symmetric aminonaphthol 140 with 2,6-dichloromethylpyridine using K_2_CO_3_ in dry DMF at room temperature for 36 h. Compound 139 exhibited excellent ability to discriminate the enantiomers of a broad variety of carboxylic acids by ^1^H-NMR spectroscopy (Scheme 40).^65^

**Scheme 41:**
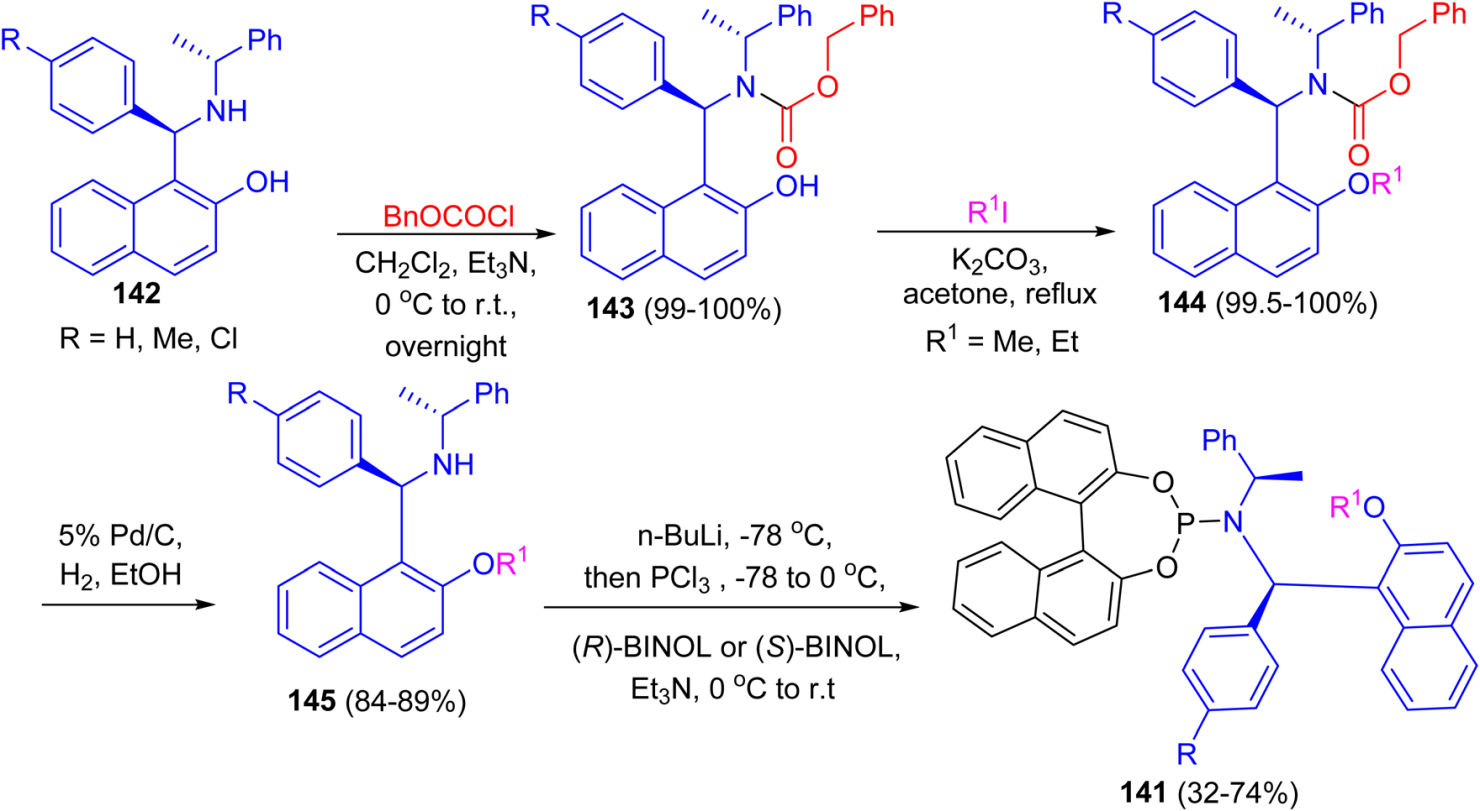
Synthesis of chiral phosphoramidite ligands 141.

After that, a series of chiral phosphoramidite ligands 141 were prepared in high yields from unsymmetrical secondary amines 142 and chiral BINOL. Alkylation of the hydroxy group of naphthols 142 with methyl or ethyl iodide in acetone after protection of the amino group with benzyl chloroformate 143 in dichloromethane provided 144 in quantitative total yields. Hydrogenolysis of carbamates 144 with 5% palladium on carbon in alcohol afforded free secondary amines 145 in good yields. The free amines reacted with phosphorus trichloride and chiral BINOL to provide chiral phosphoramidites 141 in 32–74% yield after flash chromatography (Scheme 41). The ligands with (*S*,*R*,*R*)-configuration were effective for the palladium-catalyzed enantioselective hydrosilylation of styrene derivatives. The resulting silanes were oxidized to provide secondary alcohols in medium to high yields (up to 96% yield) and good enantiomeric excesses (up to 97% ee).^66^

**Scheme 42:**
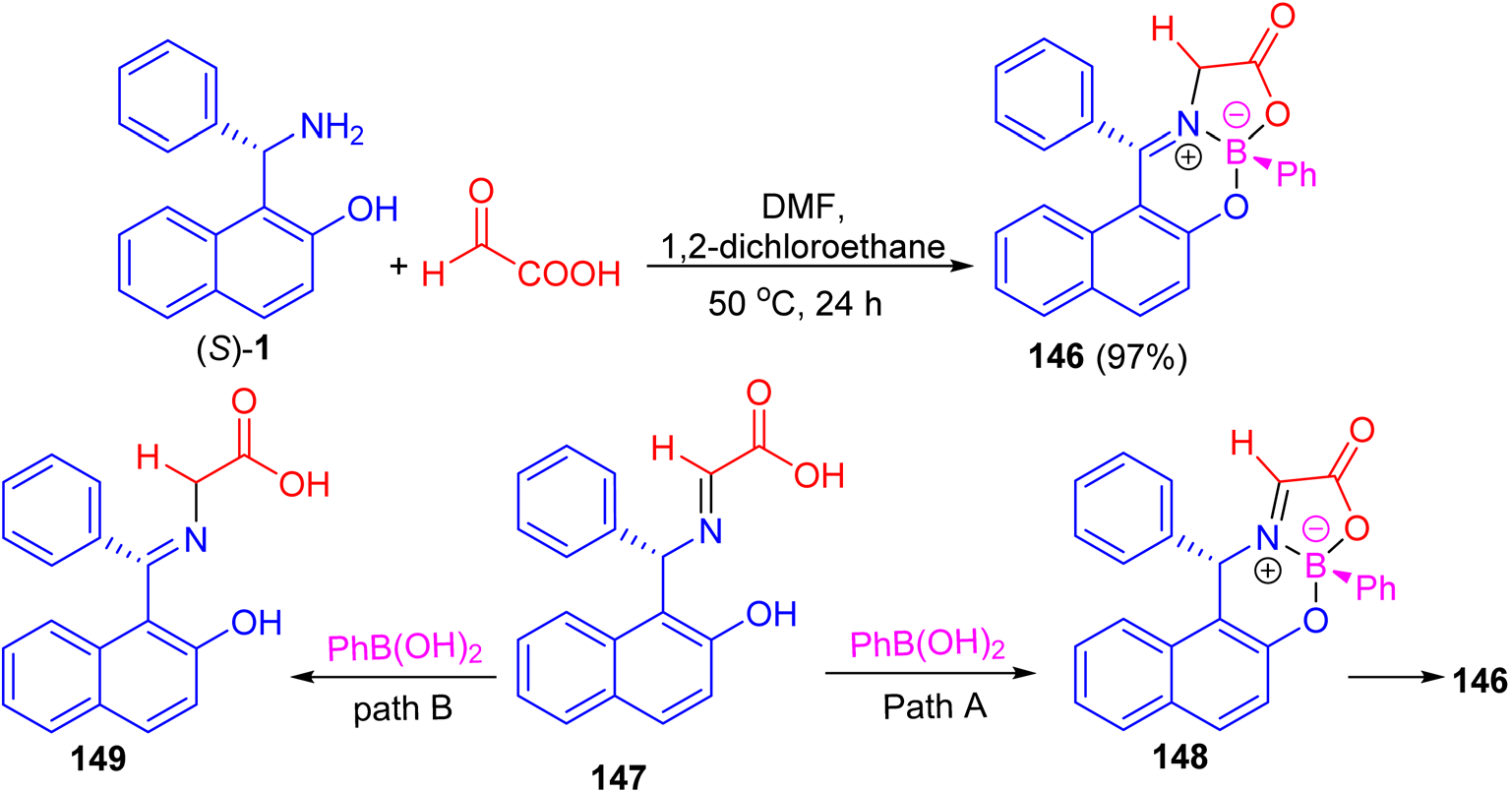
Enantioselective preparation of a stable boronate complex 146.

In addition, Hutton and co-workers constructed boronate complex 146 in 97% yield by the reaction of (*S*)-1 with glyoxylic acid and phenylboronic acid in DMF/1,2-dichloroethane at 50 °C for 24 h. Formation of 146 must involve three processes: complexation of aldimine 147 with phenylboronic acid would generate boronate complex 148, with subsequent tautomerization of the aldimine to the ketimine generating final complex 146 (path A). Alternatively, tautomerization of the aldimine to the ketimine 149 could precede complexation with the boronic acid (path B) as shown in Scheme 42. To achieve enantioselective formation of boronate complex 146, the reaction must proceed by path A, with diastereoselective formation of glyoximine–boronate complex 148 controlled by the configuration at the carbon stereocenter. The alternative reaction sequence (path B), in which tautomerization of the aldimine 147 to the achiral ketimine 149 precedes complexation with boronic acid, would result in formation of complex 146 as a racemate.^67^

**Scheme 43:**
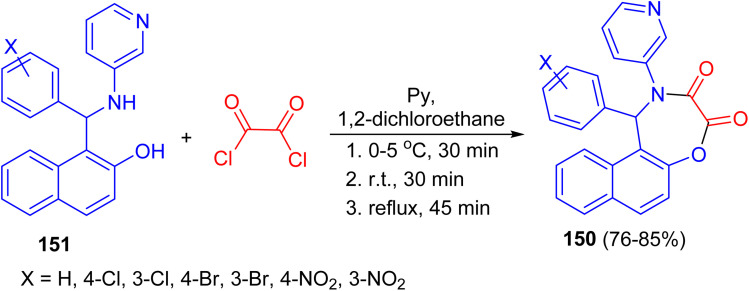
Synthesis of naphth[1,2-*f*][1,4]oxazepine-3,4-diones 150.

In 2009, Olyaei and his group reported synthesis of naphth[1,2-*f*][1,4]oxazepine-3,4-dione heterocycles 150 in 76–85% yields by the reaction of N-heteroaryl aminonaphthols 151 with oxalyl chloride in dry 1,2-dichloroethane containing pyridine under argon atmosphere. The reaction mixture was stirred for 30 min at 0–5 °C, then 30 min at room temperature, and refluxed for an additional 45 min. This method with the advantages, such as generality and simplicity of procedure, lower reaction time, elimination of acid catalyst, and obtaining excellent yields are worth noting (Scheme 43).^68^

**Scheme 44:**
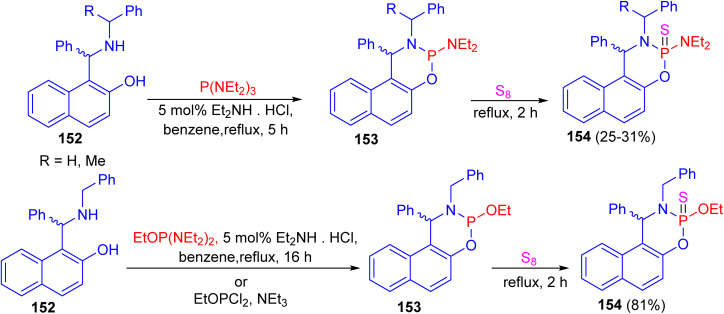
Phosphorylation of Betti bases 152.

In 2014, the reaction of *N*-substituted 1-(α-aminobenzyl)-2-naphthols 152 with phosphorus(iii) compounds in refluxing benzene in the presence of diethylamine hydrochloride as catalyst for 5–16 h afforded cyclic phosphorylated diastereomeric derivatives 153, followed by addition of elemental sulfur under reflux conditions for 2 h resulted cyclic thiophosphorylated diastereomeric derivatives 154 in 25–81% yields as depicted in Scheme 44.^69^

**Scheme 45:**
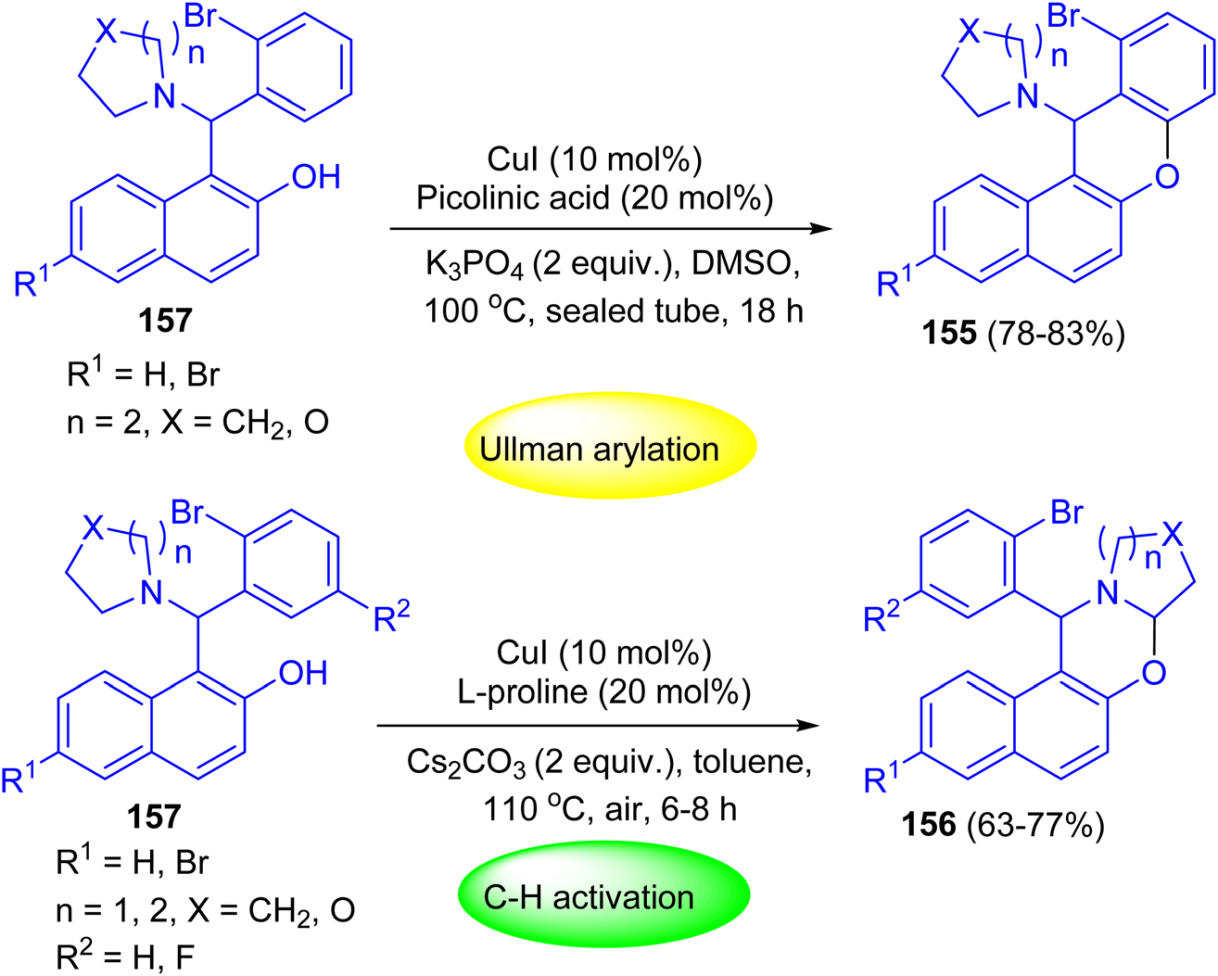
Chemoselective sequential Betti–Ullmann coupling and Betti-C–H activation reactions of 157.

After that, Shahrisa and his group reported chemoselective sequential reactions for the synthesis of 12*H*-benzo[*a*]xanthenes 155 and dihydro-1*H*-naphtho[1,2-*e*][1,3]oxazines 156. Depending on the used conditions selective, Ullmann-type arylation or α-C–H aryloxylation of 2-bromophenyl alkylaminonaphthols 157 occurred. The two-step sequence proceeded efficiently in a one-pot manner by heating of 157 in the presence of CuI (10 mol%), picolinic acid (20 mol%) and K_3_PO_4_ at 100 °C in DMSO for 18 h to afford the desired product 155 in 78–83% yields. Moreover, compounds 156 were achieved in 63–77% yields by heating of 157 in toluene at 110 °C using Cs_2_CO_3_, CuI and l-proline for 6–8 h (Scheme 45).^70^

**Scheme 46:**
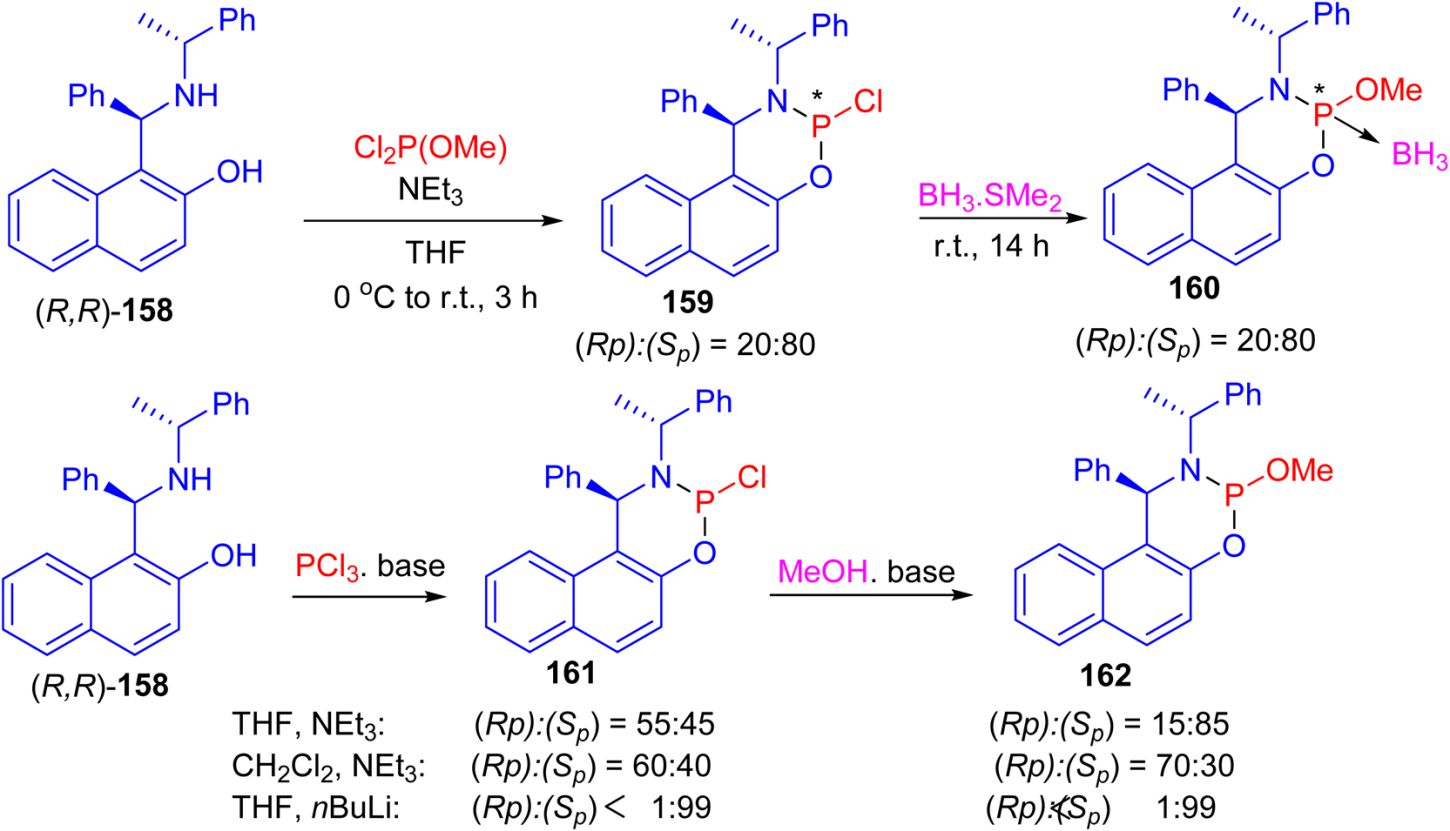
Synthesis of *P*-stereogenic phosphoramidites 159–162.

Next, the reaction of (*R*,*R*)-Betti base 158 with commercially available dichloro(methoxy)phosphine in the presence of triethylamine in tetrahydrofuran (THF) at 0 °C to room temperature for 3 h led to the formation of two phosphorus-containing species phosphoramidites 159, in a ratio of (*S*_p_) : (*R*_p_)/80 : 20. Subsequent, the mixture of P-epimers 159 was treated with a solution of BH_3_·SMe_2_ at room temperature for 14 h to give the borane adducts 160·BH_3_ in 84% yield. When the same reaction sequence was carried out in CH_2_Cl_2_ or THF with triethylamine or *n*-BuLi as base, phosphoramidochloridite 161 was formed. In the following reaction with methanol the P-epimers of the resulting phosphoramidite 160 were obtained (Scheme 46).^71^

**Scheme 47:**
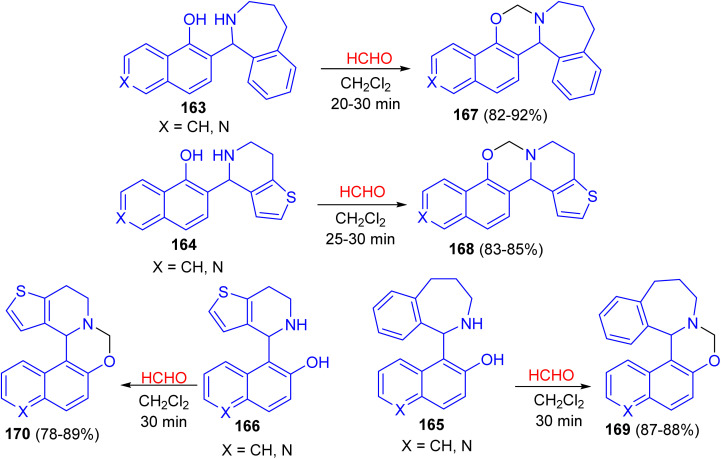
Synthesis of naphthoxazines, isoquinolinoxazines and quinolinoxazines 167–170.

In 2016, the ring closure of the Betti bases 163–166 by using a 35% solution of CH_2_O as cyclizing agent in CH_2_Cl_2_ at room temperature led to the formation of naphthoxazines, isoquinolinoxazines and quinolinoxazines 167–170 in 78–92% yields. The reactions proved to be complete after relatively short reaction times (20–30 min) and the desired products 167–170 were isolate (Scheme 47).^72^

**Scheme 48:**
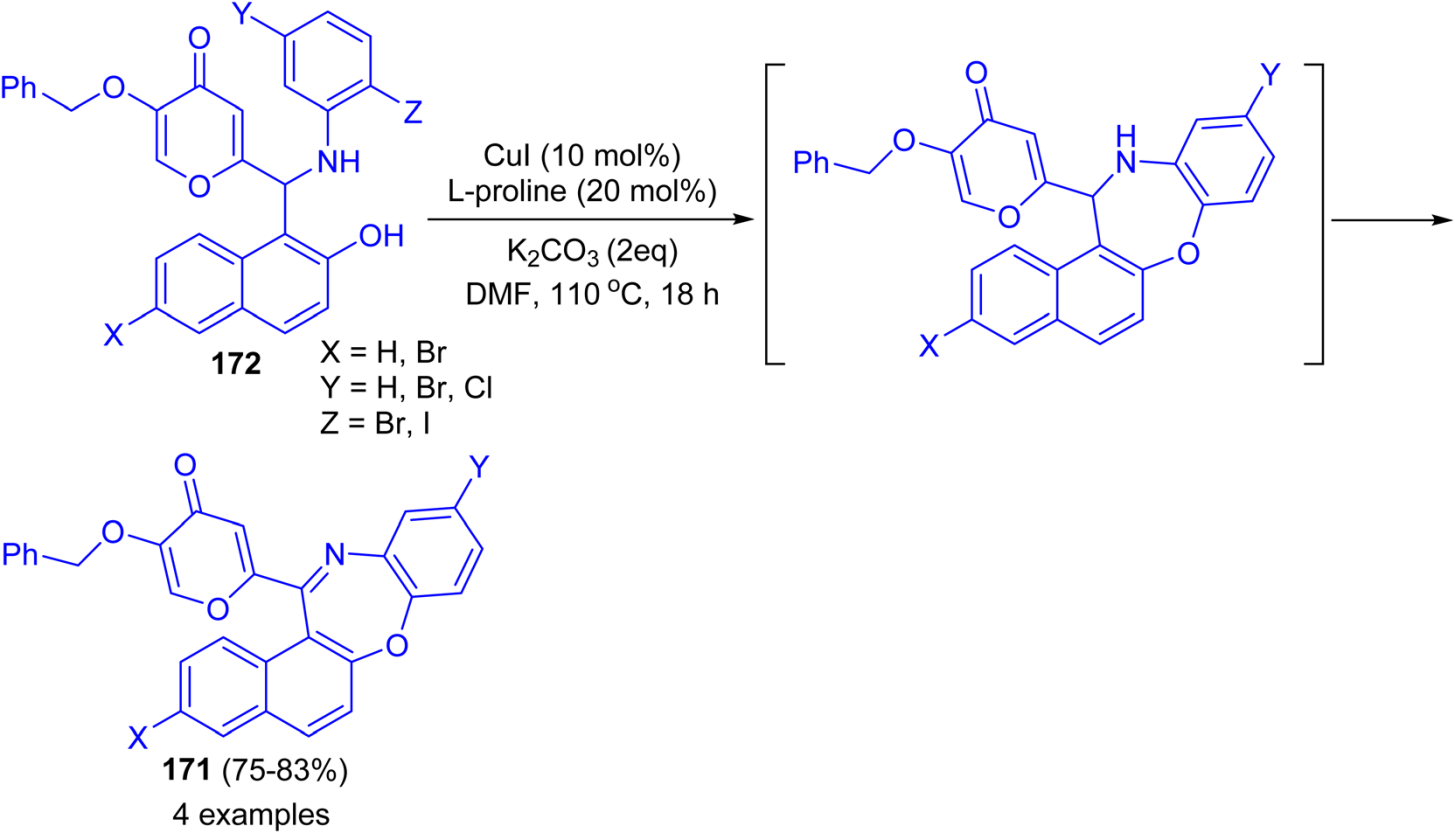
Synthesis of oxazepine derivatives 171.

In 2017 Teimuri-Mofrad *et al.* reported synthesis of oxazepine derivatives 171 in 75–83% yields *via* intramolecular Ullmann reaction of Betti bases 172 in the presence of catalytic amount of CuI, l-proline and K_2_CO_3_ in DMF at 110 °C for 18 h followed by subsequent oxidation reaction as shown in Scheme 48.^73^

**Scheme 49:**
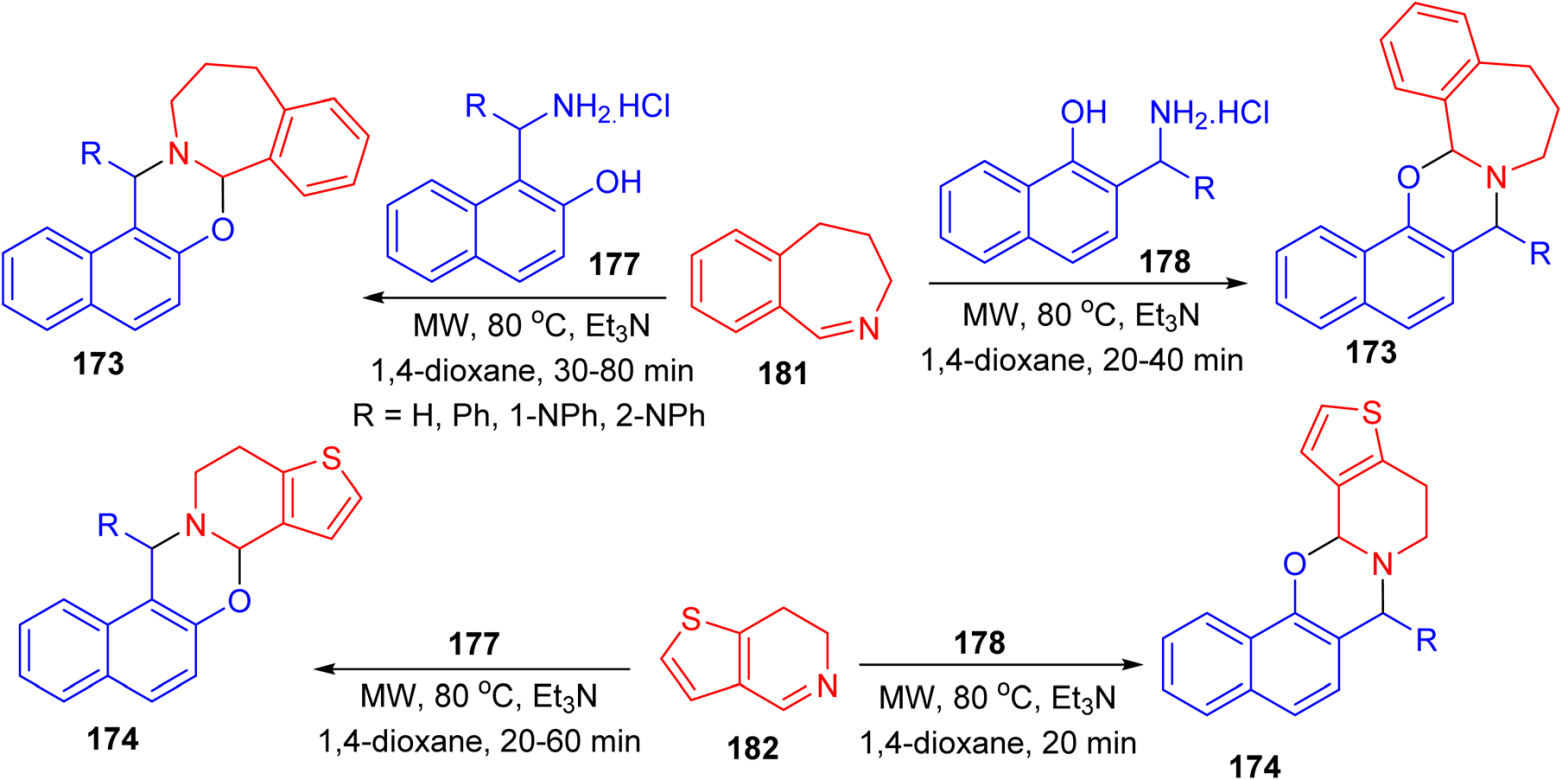
Synthesis of naphth[1,3]oxazino-benzazepines 173 and -thienopyridines 174.

Further, a series of naphth[1,3]oxazino-benzazepines 173 in 7–48% yields and -thienopyridines 174 in 10–49% yields were synthesized using a modified Mannich-type synthetic pathway by the reaction of 4,5-dihydro-3*H*-benz[*c*]azepine 175 or 6,7-dihydrothieno[3,2-*c*]pyridine 176 and different substituted aminonaphthols 177 and 178 using microwave irradiation in 1,4-dioxane in the presence of Et_3_N at a temperature of 80 °C for 20–80 min. In these reactions, the mixture of diastereomers was formed and the conversion and the diastereomeric ratio were monitored by crude product NMR spectra in all cases (Scheme 49).^74^

**Scheme 50:**
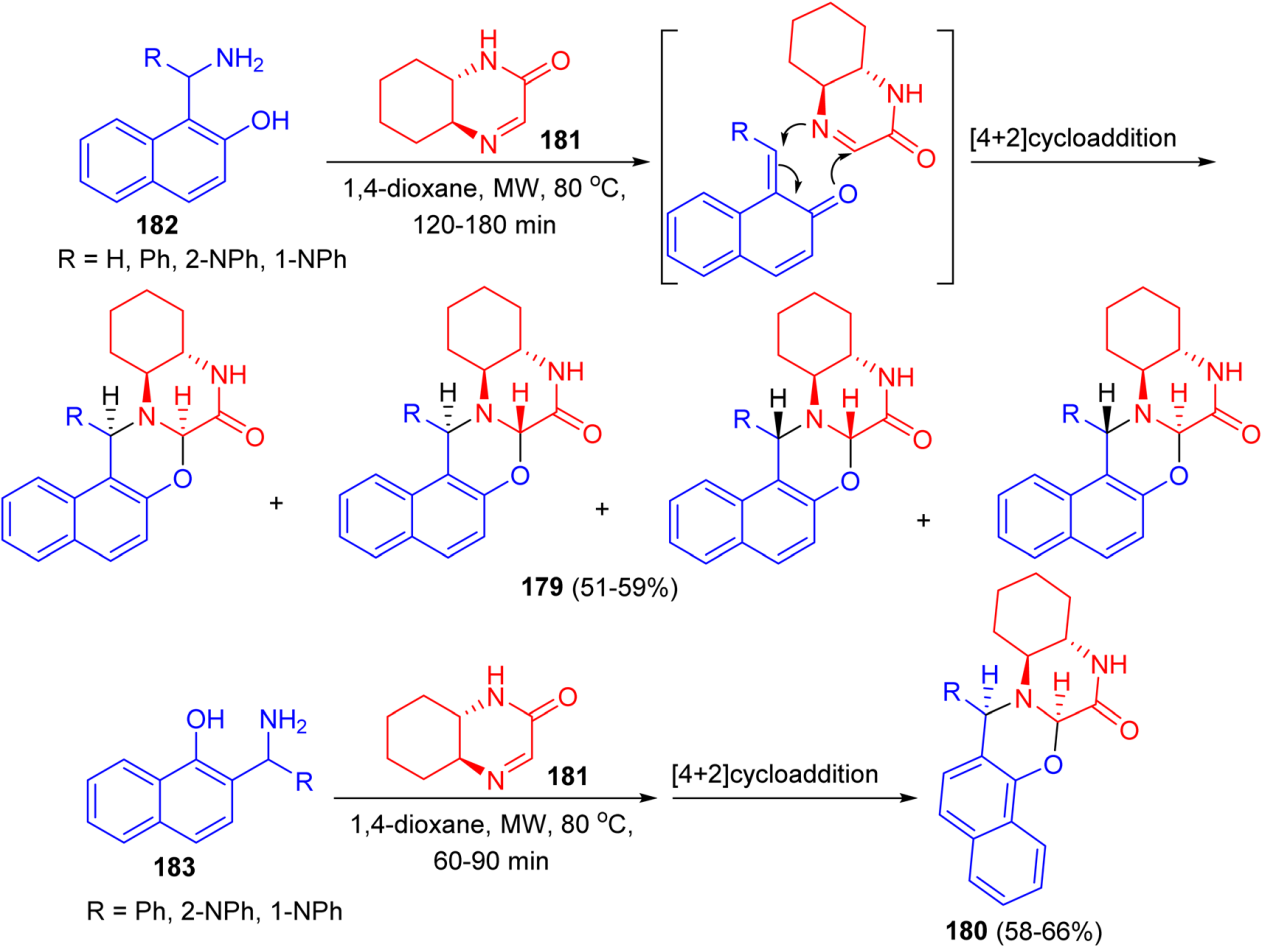
Preparation of naphth[1,3]oxazino[3,2-*a*]quinoxalinones 179 and 180.

Fulop and co-workers reported synthesis of non-racemic naphth[1,3]oxazino[3,2-*a*]quinoxalinones 179 and 180 in 51–66% yields starting from (4*aS*,8*aS*)-4*a*,5,6,7,8,8*a*-hexahydro-2-quinoxalinone 181 and 1-aminoalkyl-2-naphthols 182 or 2-aminoalkyl-1-naphthols 183 in 1,4-dioxane under microwave irradiation at 80 °C for 60–180 min (Scheme 50). In all cases, NMR studies were performed to analyse the structures and diastereoselectivities of the products.^75^

**Scheme 51:**
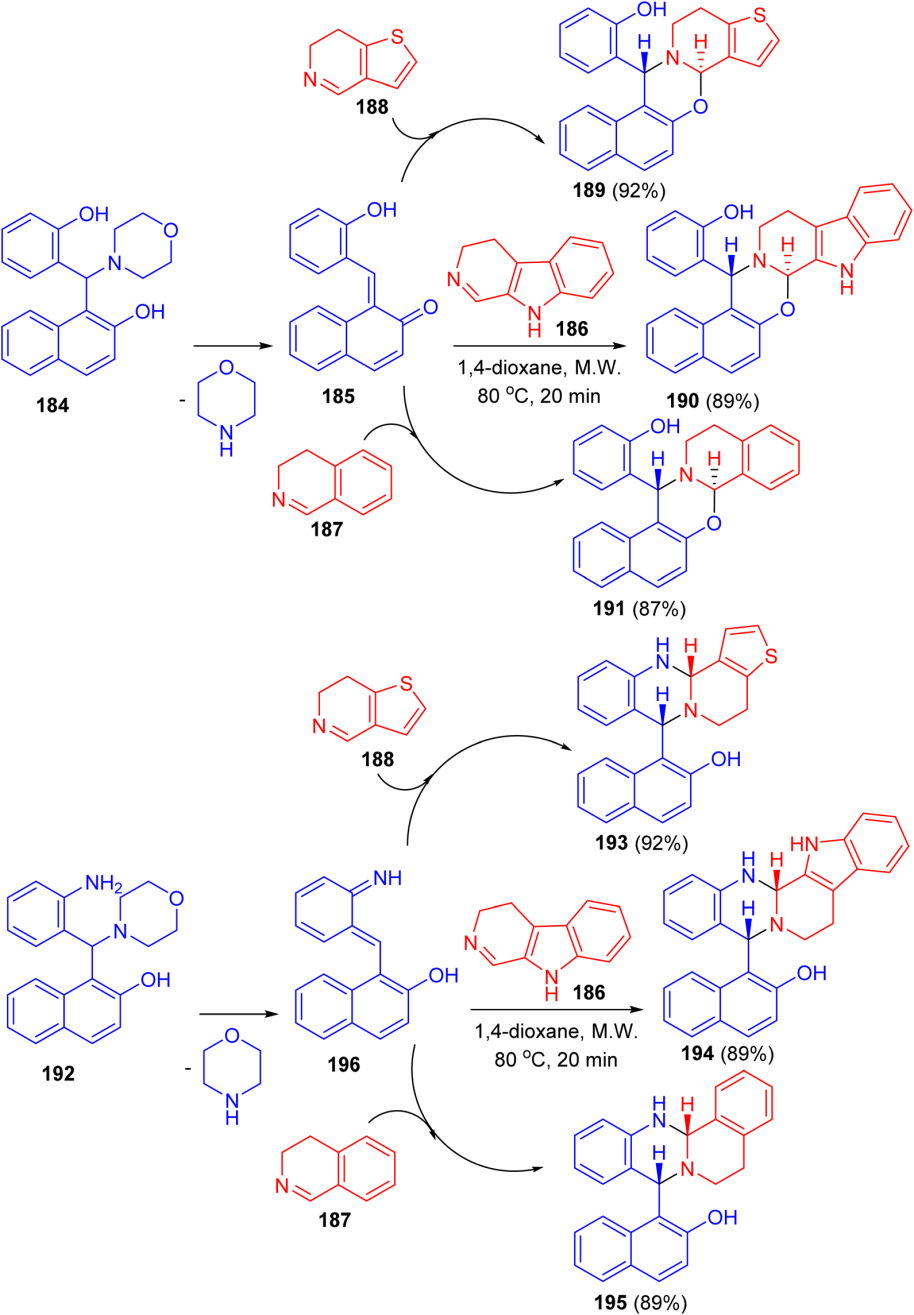
Synthesis of O,N- or N,N-heterocycles 189–191 and 193–195.

In 2019, Szatmari and co-workers described synthesis of O,N- or N,N-heterocycles by the reactions of highly functionalized aminonaphthol 184 with different cyclic imines *via* [4 + 2] cycloaddition. Initially, *o*-QM intermediate 185 was formed by thermal decomposition of 184. Then, the reaction of 185 with β-carboline 186, 4,5-dihydro-3*H*-benz[*c*]azepine 187 and 6,7-dihydrothieno[3,2-*c*]pyridine 188 in 1,4-dioxane under microwave irradiation at 80 °C for 20 min led to the formation of aminonaphthol 192 with different cyclic imines 186–188 under the same reaction conditions afforded quinazolines 193–195 in 89–92% yields. The reactions were found to be diastereo- and regioselective naphthoxazines 189–191 in 87–92% yields. Also, the reaction of leading to *trans* naphthoxazine. Its structure was proved by DFT computed structures in comparison with the experimental ^1^H/^13^C-NMR spectra, and a detailed analysis of the spatial magnetic properties of the preferred diastereomers. The possible reaction pathway *via* formation of the *o*-QM intermediate 196 (Scheme 51).^76^

**Scheme 52:**
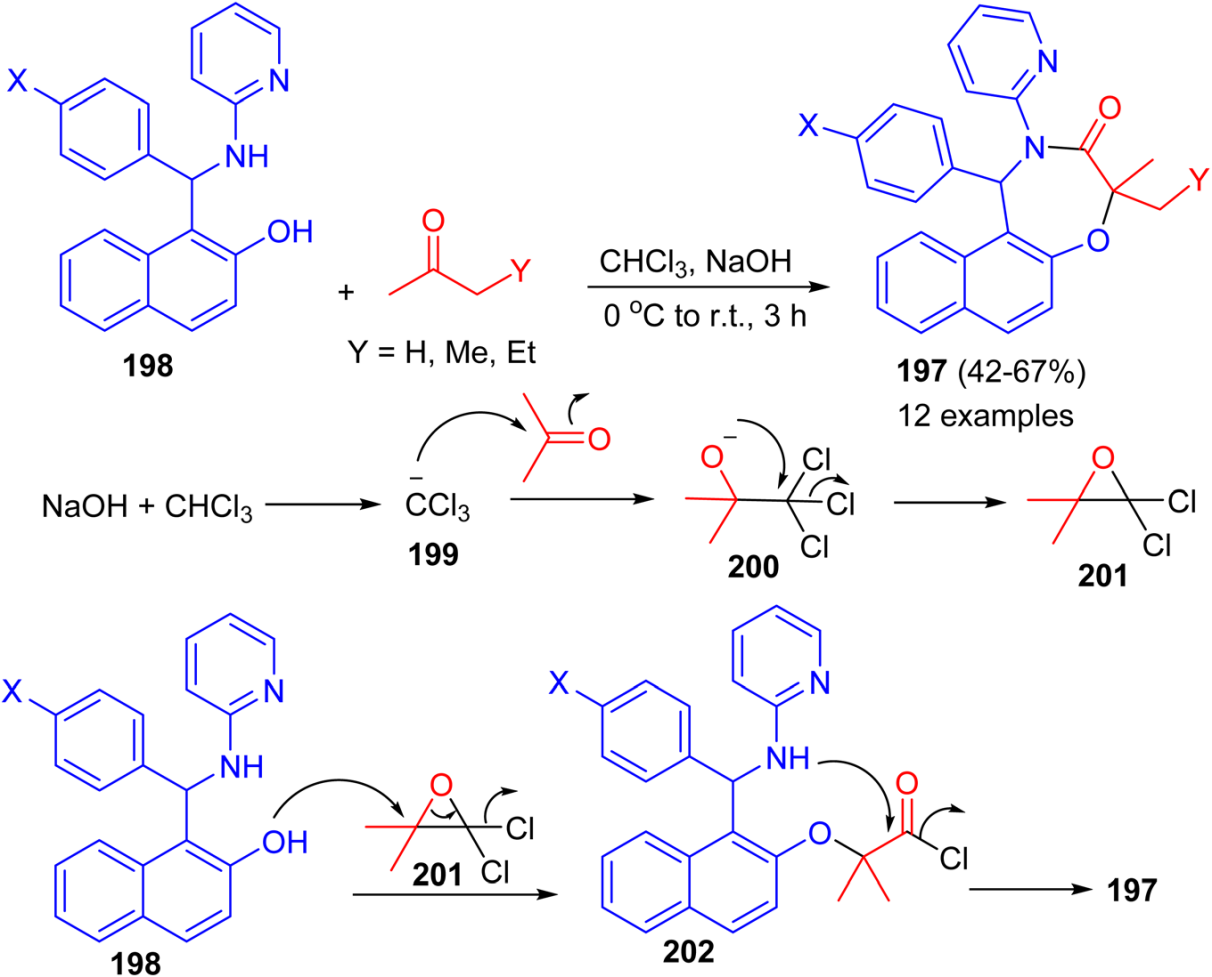
Synthesis of naphtho[1,2-*f*][1,4]oxazepines 197.

In 2022, Shaabani *et al.* reported an efficient and convenient approach for the synthesis of naphtho[1,2-*f*][1,4]oxazepines 197 in 42–67% yields based on the consecutive Betti/Bargellini multicomponent reactions of aminobenzylnaphthols 198 with aliphatic ketones in chloroform in the presence of sodium hydroxide at 0 °C to room temperature for 3 h. A plausible mechanism for the formation of 197 is depicted in Scheme 52. At first, sodium hydroxide abstracts a proton from chloroform to afford the carbanion 199, which subsequently attacks acetone and generates the unstable carbinol 200. Next, the intermediate 200 cyclizes to produce the Bargellini epoxide 201. Afterward, the intermediate 202 is formed *via* the attack of oxygen nucleophile of the Betti base to intermediate 201. Finally, intramolecular cyclization occurs and leads to the formation of desired oxazepine scaffold 197.^77^

**Scheme 53:**
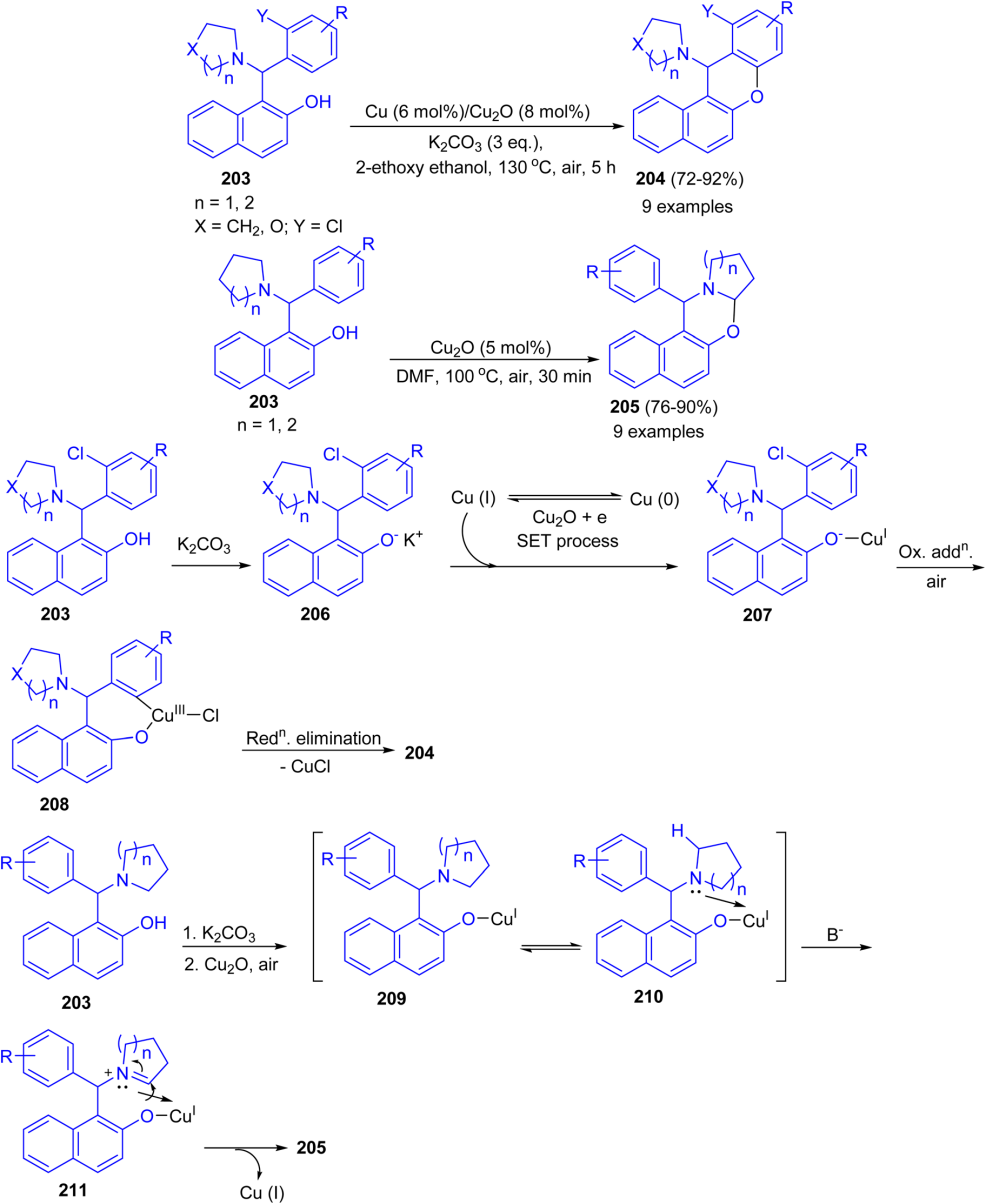
Synthesis of benzo-xanthenes 204 and 1,3-oxazine derivatives 205.

Ahmed and co-workers developed Cu–Cu_2_O combination synergic effects in catalyzing intramolecular Ullmann coupling reaction for halo-Betti bases 203 in 2-ethoxyethanol at 130 °C for 5 h to afford fused benzo-xanthenes 204 in 72–92% yields from both electron-rich and electron-deficient aromatic systems in good yield under mild reaction conditions. However, without *ortho*-halo substituted Betti-bases failed to afford fused benzo-xanthene derivatives. Exploring the reaction optimization without Cu metal, serendipitously it was produced 1,3-oxazine derivatives 205 in 76–90% yields in DMF at 100 °C for 30 min *via* intramolecular cross-dehydrogenative coupling (CDC) reaction. Similarly, electron-rich, electron-deficient and sterically hindered Betti-bases provided the products in good to excellent yield under mild condition. A possible mechanism for the formation of 204 is proposed in Scheme 53. The abstraction of proton from 203 by K_2_CO_3_ led to Betti base anion 206 (soft nucleophile) which reacts with Cu(i) to give intermediate 207. Then, intermediate 207 undergoes intramolecular Ullmann oxidative addition step to afford intermediate 208. Followed by, reductive elimination of CuX led the product 204 formation. In the proposed mechanism for the synthesis of 205, abstraction of proton from Betti base 203 by K_2_CO_3_ led to Betti base anion 209 (soft nucleophile) which reacts with Cu(i) to form intermediate 210. Then, intermediate 210 undergoes coordination with heterocycle N-atom which causes positive charge on atom. This resulted in alpha hydrogen abstraction in basic condition to afford imine intermediate 211. Finally, nucleophilic addition to imine intermediate 211 gave the product 205.^78^

**Scheme 54:**
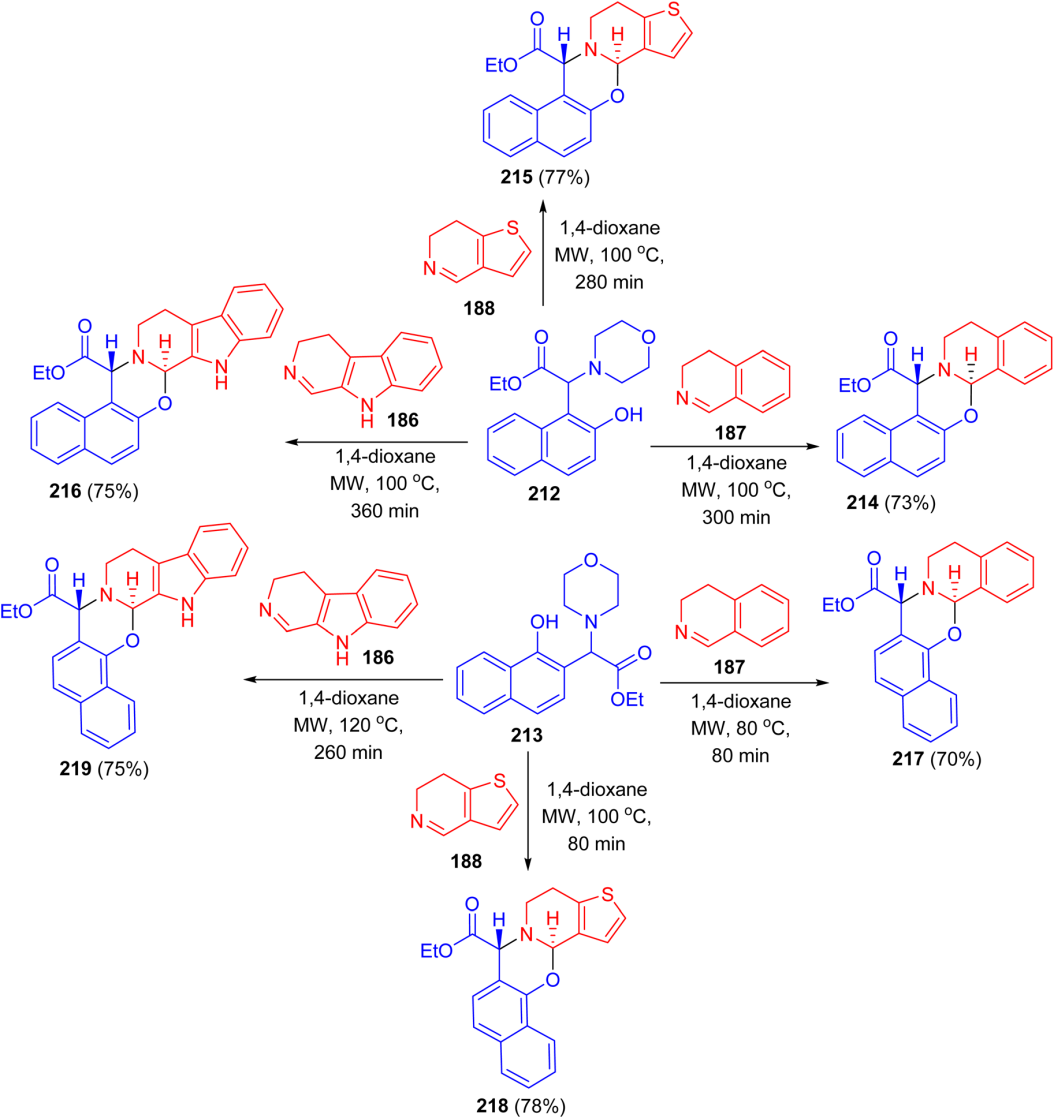
Synthesis of naphthoxazines 214–219*via* [4 + 2] cycloaddition reactions.

After that, the reaction of Betti bases 212 and 213 with different cyclic imines (3,4-dihydroisoquinoline, 6,7-dihydrothieno[3,2-*c*]pyridine, 3,4-dihydro-β-carboline) in [4 + 2] cycloaddition in 1,4-dioxane under MW irradiation at 100 °C for 80–360 min afforded the desired naphthoxazines 214–219 in 70–78% yields (Scheme 54). During the reaction *via* loss of morpholine, the presence of partially aromatic *ortho*-quinone methide intermediates proved and they were transformed with dienophiles to new α-amino acid esters. Regarding the biological results, it can be concluded that in the case of some compounds antibacterial effect was observed on the reference *S. aureus* ATCC 25923 strain.^79^

## 8 The other reactions

**Scheme 55:**
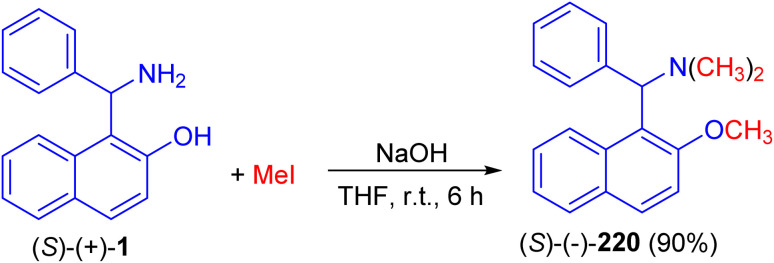
Synthesis of [(2-methoxynaphth-1-yl)benzyl]dimethylamine (220).

In 1998, Naso and co-workers reported synthesis of [(2-methoxynaphth-1-yl)benzyl]dimethylamine (220) in 90% yield by the reaction of 1-(α-aminobenzyl)-2-naphthol (1) with CH_3_I in the presence of NaOH in THF at room temperature for 6 h as illustrated in Scheme 55.^80^

**Scheme 56:**
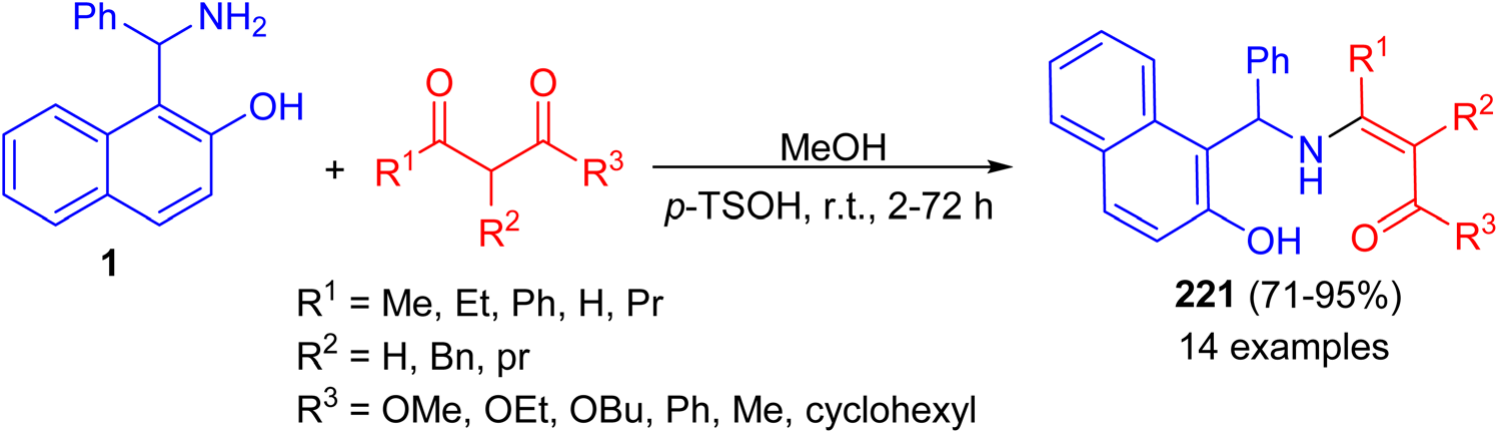
Condensations of Betti base 1 with 1,3-dicarbonyl compounds.

Next, the reaction of the Betti base 1-(α-aminobenzyl)-2-naphthol (1) with 1,3-dicarbonyl compounds at room temperature in CH_3_OH in the presence of *p*-toluenesulfonic acid led to the corresponding enamino carbonyl products 221 after 2–72 h, in high yield (71–95%) and with high chemical purity (Scheme 56).^81^

**Scheme 57:**
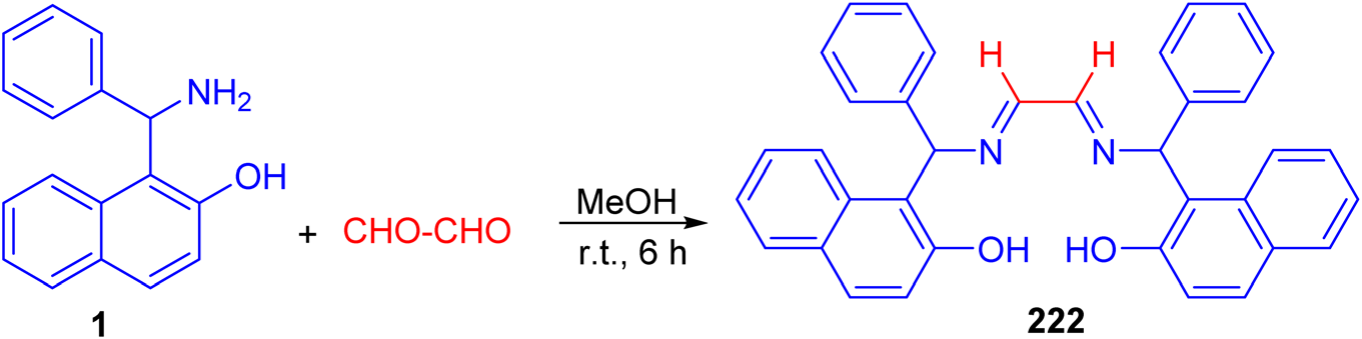
Synthesis of tetradentate Betti base derivative 222.

After that, Betti base-derived tetradentate ligand 222 was designed and synthesized in good yield through the condensation of Betti base 1 with aqueous glyoxal in MeOH at room temperature for 6 h (Scheme 57). The application of the ligand 222 in copper-catalyzed *N*-arylation of imidazoles was investigated briefly, and good yields of the products were obtained.^82^

**Scheme 58:**
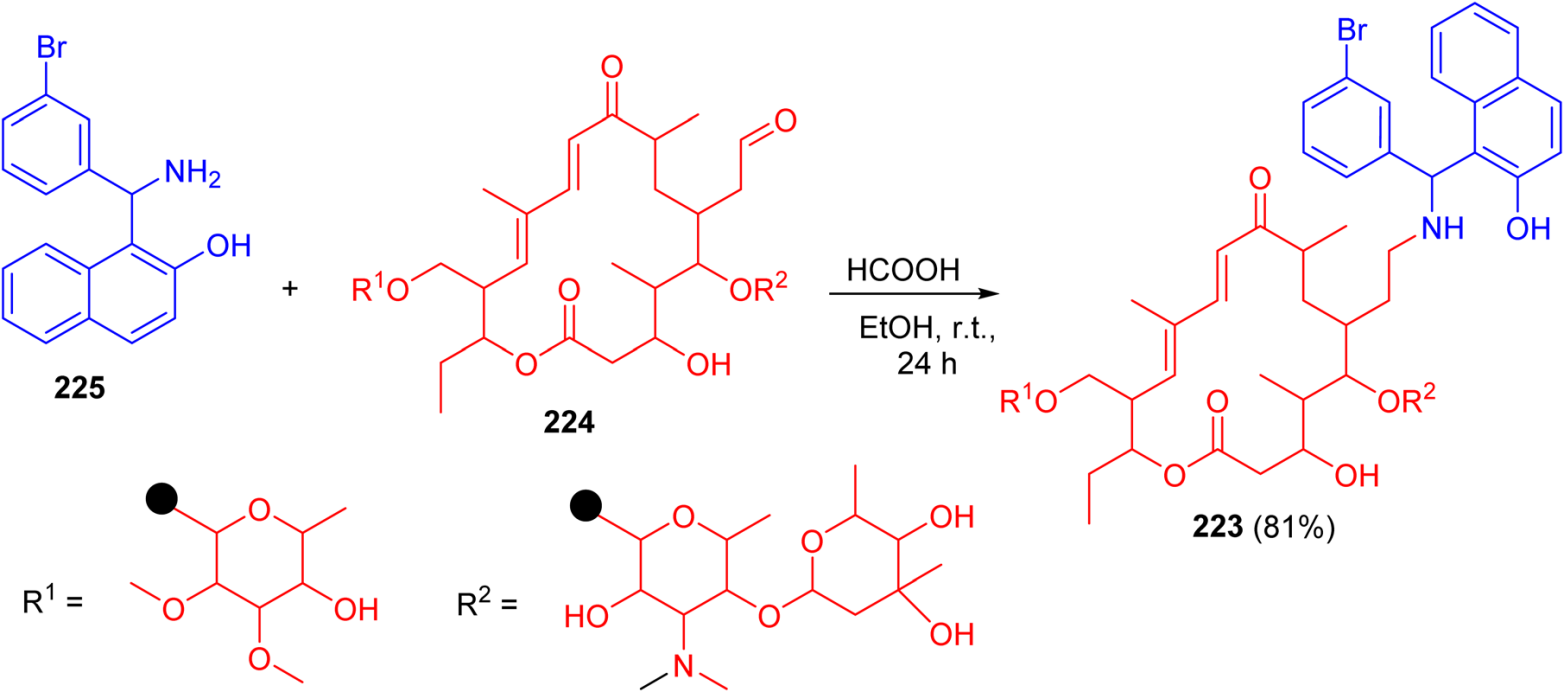
Preparation of *N*-tylosil-1-α-amino-(3-bromophenyl)-methyl-2-naphthol (223).

In 2010, Witte *et al.* described synthesis of *N*-tylosil-1-α-amino-(3-bromophenyl)-methyl-2-naphthol (TBN) (223) in 81% yield by the reaction of Tylosin (224) with Betti-base 225 in EtOH in the presence of HCOOH at room temperature for 24 h (Scheme 58).

TBN dramatically increased the P-gp-mediated cellular uptake of the fluorescent substrate rhodamine 123. Similarly, TBN was found to act as a very potent enhancer of the cytotoxicity of doxorubicin on the resistant cell line.^83^

**Scheme 59:**
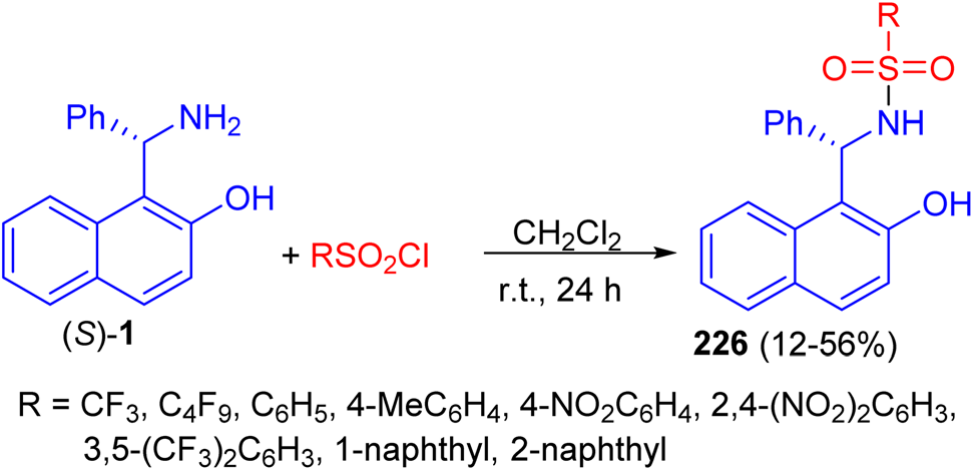
Synthesis of mono-sulfonamide organocatalysts 226.

In 2011, a variety of mono-sulfonamide organocatalysts 226 prepared in 12–56% yields by coupling of (*S*)-1 with various sulfonyl chlorides in CH_2_Cl_2_ at room temperature for 24 h and applied as catalyst to the asymmetric hetero-Diels–Alder reaction of ethyl glyoxylate with Danishefsky's diene. The sulfonamides exhibited catalytic activity as hydrogen bond donor (Scheme 59).^84^

In 2015, Baruah *et al.* converted 1-(α-aminoalkyl) 2-naphthols 227 to 1-alkyl-2-naphthols 228*via* a metal-free transfer hydrogenation with Hantzsch 1,4-dihydropyridine 229 in CH_3_CN using *p*-TSA (10 mol%) as catalyst under microwave irradiation (500 W) for 5–9 min.

**Scheme 60:**
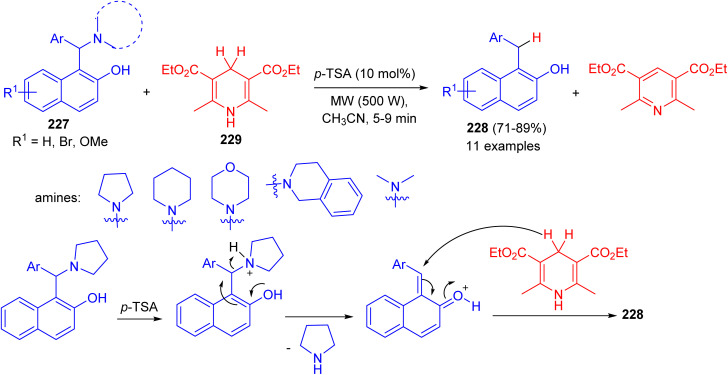
Synthesis of 1-alkyl-2-naphthols 228.

The corresponding aryl methylnaphthols which contain a wide range of substituents, could be obtained in 71–89% yields. The reaction does not use any hazardous metal catalyst or reductant. The method uses *p*-TSA as catalyst which is a convenient, easily available and cheap reagent. Moreover, the reaction is general and having wide substrate compatibility. A mechanistic proposal involving elimination–addition mechanism is described in Scheme 60.^85^

**Scheme 61:**
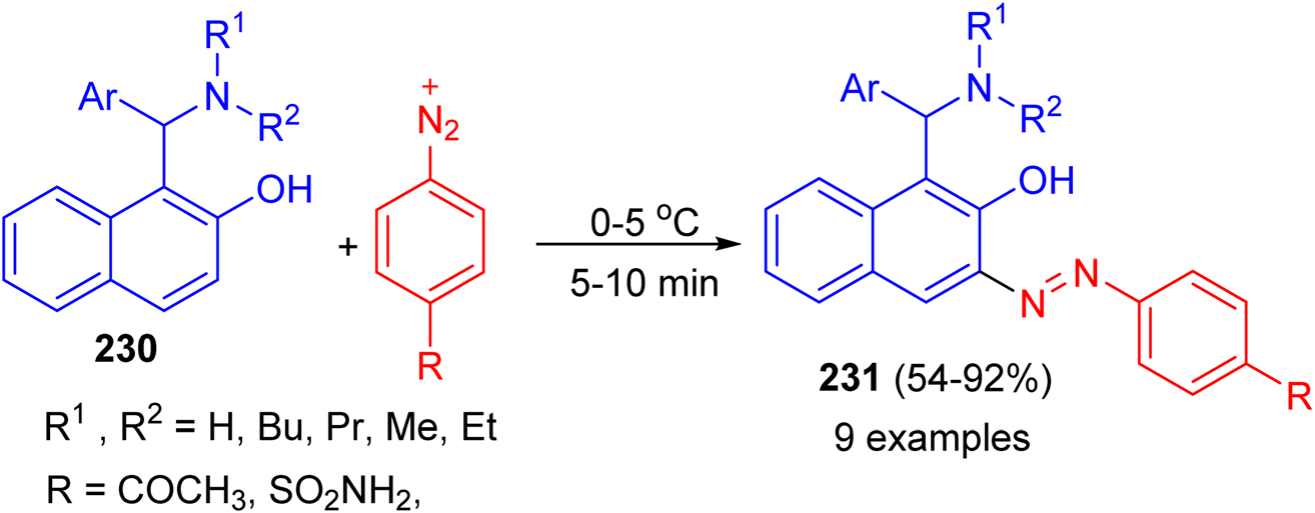
Synthesis azo dye compounds 231.

In addition, the Betti bases 230 were diazotized by treating with equivalent amounts of sulfanilamide or *p*-aminoacetophenone in the presence of sodium nitrite and hydrochloric acid at 0–5 °C. After 5–10 min, azo dye compounds 231 obtained in 54–92% yields (Scheme 61).^86^

**Scheme 62:**
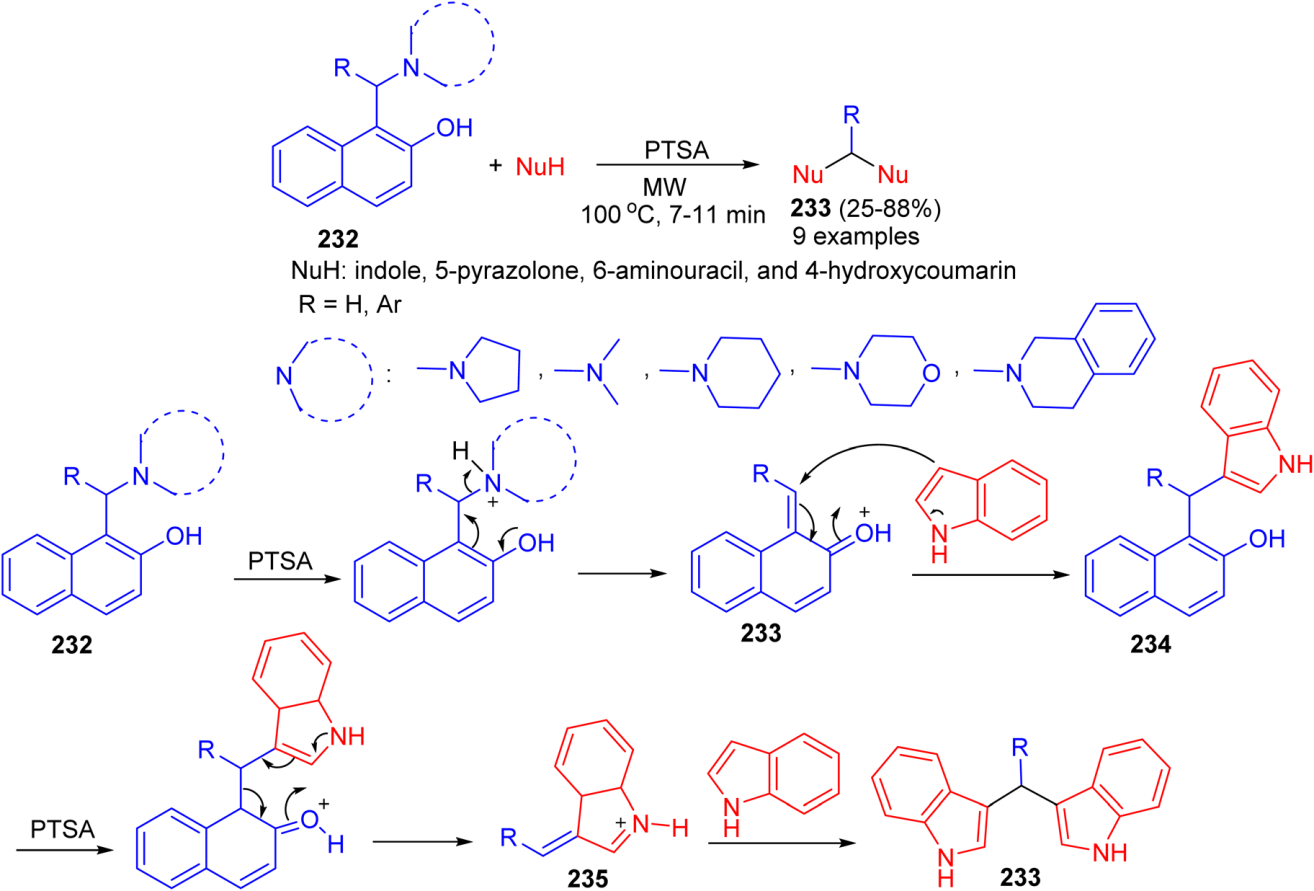
Synthesis of bis(heterocycle)methanes 233.

In 2016, Deb and co-workers reported the reaction of Betti bases 232 with various heterocycles like indole, 5-pyrazolone, 6-aminouracil, and 4-hydroxycoumarin for the formation of bis(heterocycle)methanes 233 using *p*-toluenesulphonic acid in CH_3_CN under microwave irradiation at 100 °C for 7–11 min. A plausible mechanism for the reaction is proposed in Scheme 62. *p*-TSA first helps to eliminate the *tert*-amine moiety of the Betti base by protonating nitrogen, which generates *o*-quinone methide 233. Subsequently a molecule of indole attacks 233, producing 3-(α,α-diarylmethyl) indole 234. In the presence of *p*-TSA, a naphthol ring of 234 gets protonated and eliminated as free naphthol through the formation of alkylideneindoleninium ion 235. Then another molecule of indole attacks 235, producing bis(indolyl)methanes 233 in 25–88% yields.^87^

**Scheme 63:**
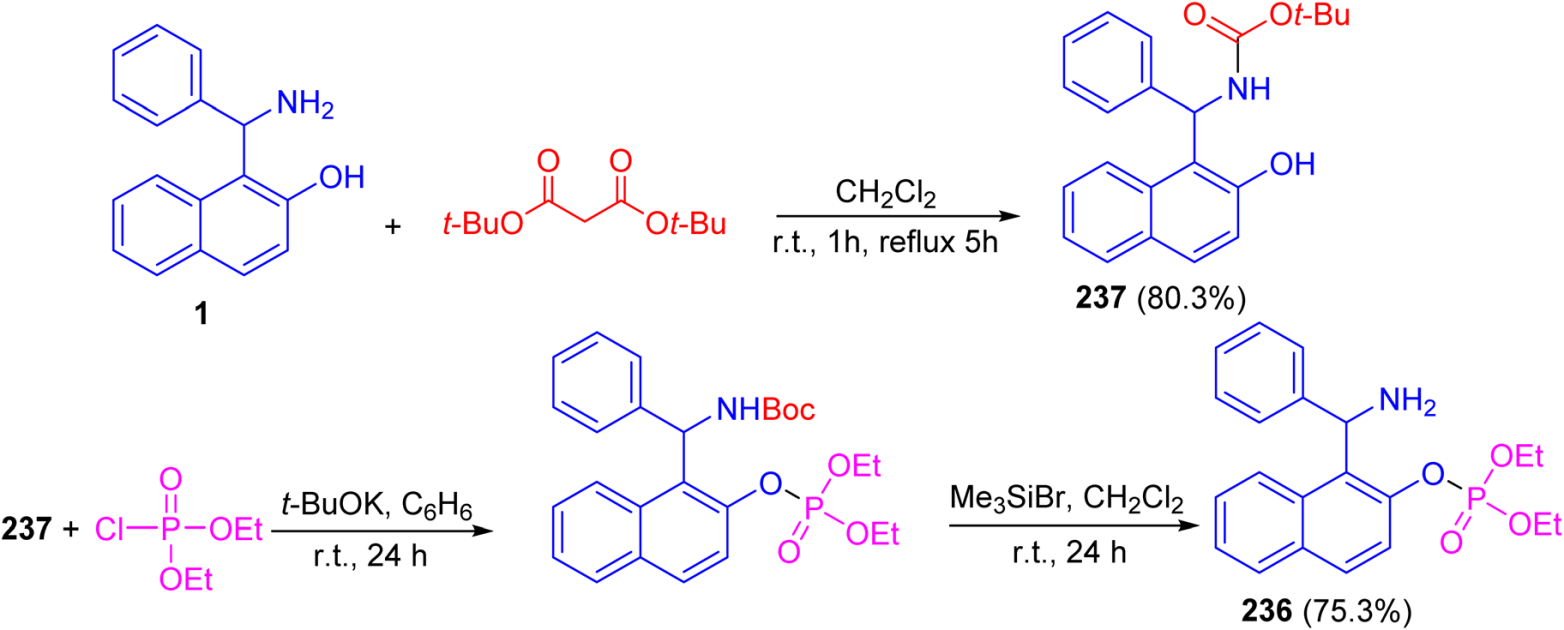
Synthesis of (±)-*O*-1-[amino(phenyl)methyl]naphth-2-yl phosphate 236.

Alfonsov and co-workers published the synthesis of phosphoric acid derivatives containing chiral Betti base fragment 236 by reacting racemic and enantiopure *N*-Boc-protected 1-(α-aminobenzyl)-2-naphthol 237 with diethyl chlorophosphate in anhydrous benzene using potassium *tert*-butylate for 24 h followed by deprotection with bromotrimethylsilane in CH_2_Cl_2_ at room temperature under argon for 24 h. It should be noted that *N*-Boc-protected Betti base 237 synthesized in 80.3% yield by the reaction of (±)- and (*S*)-(+)-1-(α-aminobenzyl)-2-naphthol (1) with di-*tert*-butyl dicarbonates in CH_2_Cl_2_ at room temperature for 1 h followed by refluxing for 5 h. This method is simple and effective approach to synthesis of racemic and enantiopure phosphoric acids containing chiral Betti base fragment with free amino group (Scheme 63).^88^

**Scheme 64:**
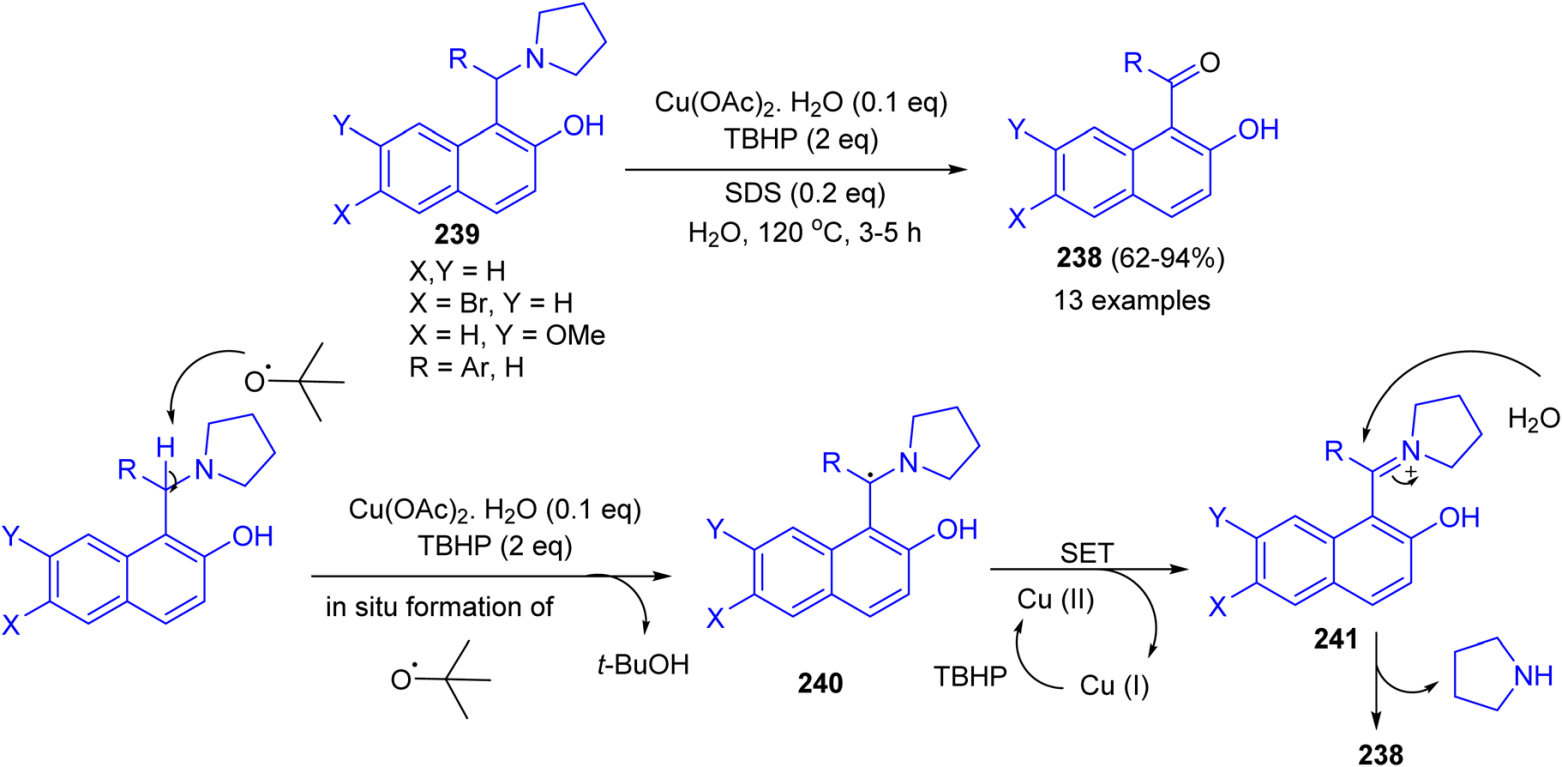
Copper catalyzed synthesis of hydroxy aryl ketones 238.

Next, an efficient route for benzoylation or formylation of naphthols for the synthesis of hydroxyaryl ketones 238 developed *via* oxidative deamination of Betti bases 239 in the presence of copper salt catalyst with TBHP as an oxidant in water as a reagent as well as solvent. The products were obtained in 62–94% yields after 3–5 h. The obtained results showed that *o*/*m* substituted aryl ring of Betti base offered relatively lower yield of product with longer time. Electron withdrawing group on the R of Betti base increases the product yield, whereas electron releasing group decreases the yield. A tentative mechanism for the reaction is proposed in Scheme 64. The benzylic radical 240 could be formed through H-abstraction by *tert*-butoxy radical (formed by copper catalyzed decomposition of TBHP), which followed by a single-electron transfer (SET) from 240 to generate the benzylic iminium carbocation 241. Subsequently, the intermediate 241 was attacked by a molecule of water giving ketone 238.^89^

**Scheme 65:**
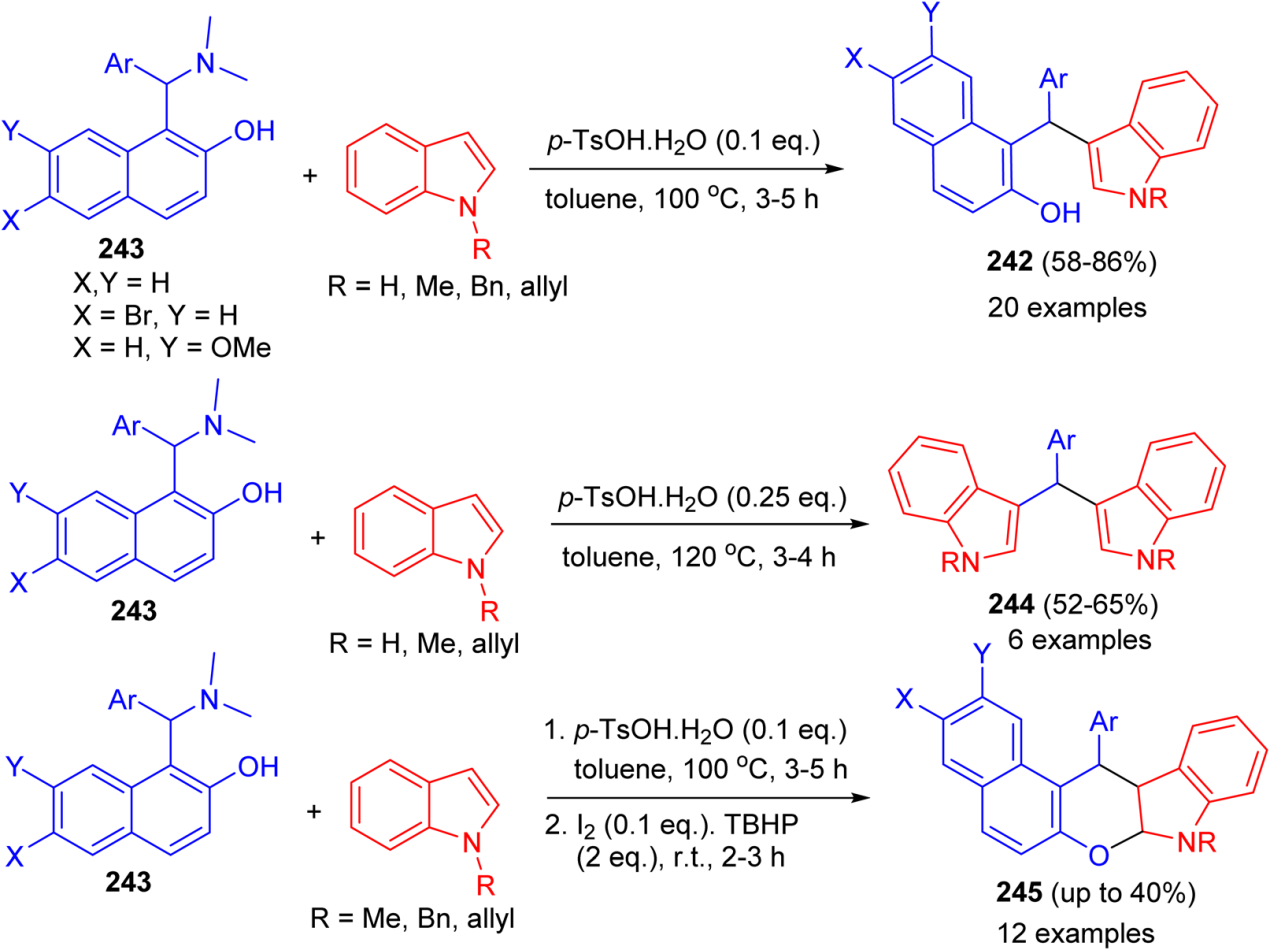
Brønsted-acid-mediated divergent reactions of Betti bases with indoles.

Baruah and co-workers published the synthesis of compounds 242 in 58–86% yields by the reaction of Betti bases 243 with indoles using *p*-TsOH·H_2_O (0.1 eq.) as catalyst in toluene at 100 °C for 3–5 h. Electron-donating groups on the aryl ring of Betti bases decreased the product yield, whereas electron-withdrawing groups increased the yield. Betti bases having *ortho*-substituted aryl rings provided lower yields with longer reaction times. When the reaction was carried out by 2.0 equivalents of indoles in the presence of *p*-TsOH·H_2_O (0.25 eq.), bis(indolyl)methanes 244 were obtained in 52–65% yields after 3–4 h. Also, the reaction of 243 with indoles using *p*-TsOH·H_2_O (0.1 equiv.) in toluene at 100 °C for 3–5 h and then I_2_ (0.1 equiv.) and TBHP (2.0 equiv.) afforded chromeno[2,3-*b*]indoles 245 after 2.5 h by stirring the mixture at room temperature. Moreover, the reactions do not use any expensive metal catalyst or solvent. Neither dry solvents nor precautions for an inert atmosphere are required (Scheme 65).^90^

**Scheme 66:**
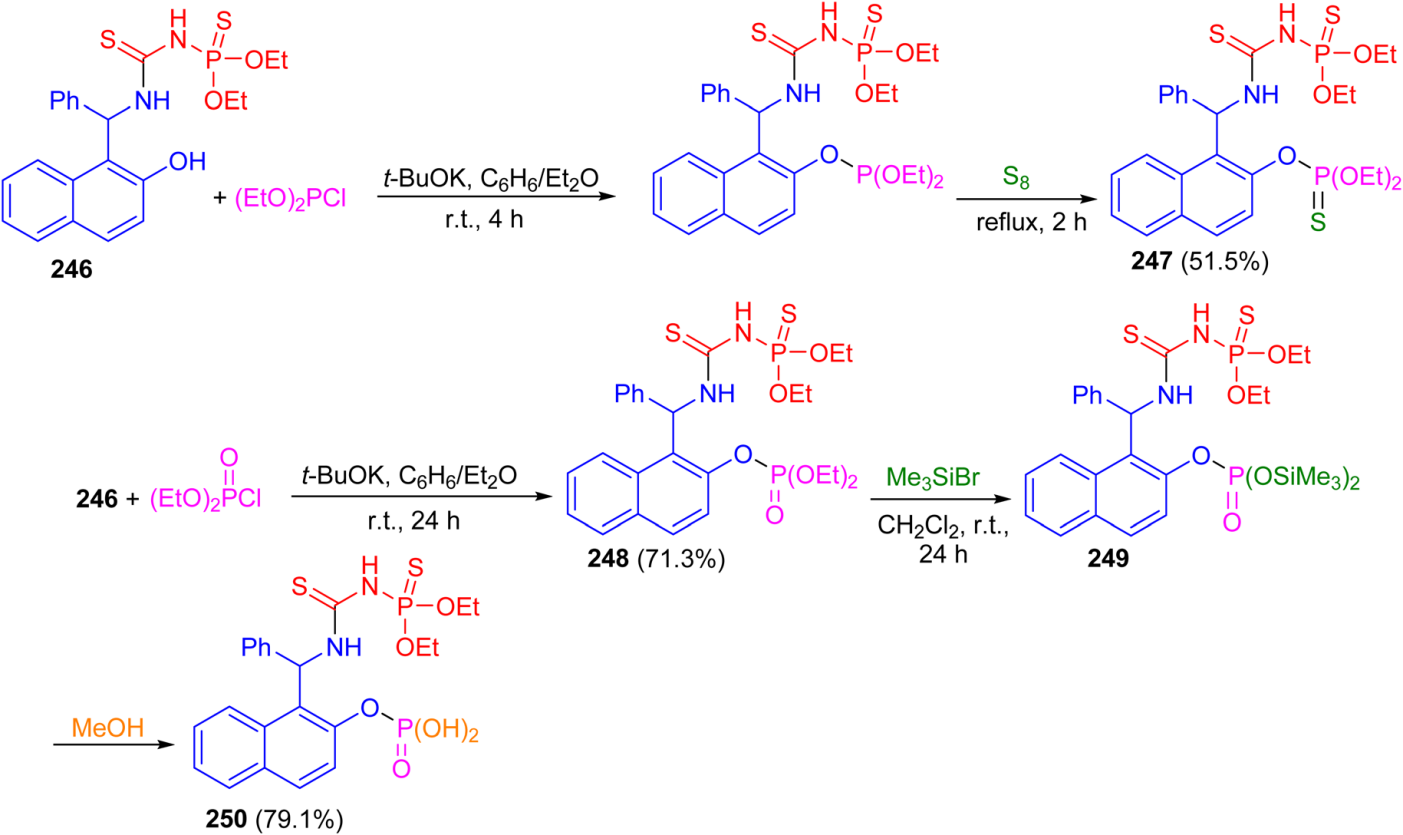
Synthesis of thiourea derivatives 247–250.

In 2017, the reaction of racemic urea 246 with diethyl chlorophosphite in the presence of potassium *t*-butoxide in benzene/diethyl ether at room temperature for 4 h followed by the treatment with elemental sulfur at reflux conditions for 2 h furnished bis-thiophosphorylated thiourea 247 in 51.5% yield. Then, the reaction of thiourea 246 with diethyl chlorophosphate in the presence of potassium *tert*-butoxide as the base in benzene/diethyl ether at room temperature for 24 h resulted thiourea 248 in 71.3% yield. Also, the reaction of 248 with an excess of bromotrimethylsilane in methylene chloride at room temperature for 24 h afforded bisilylated phosphorylthiourea 249 followed by the addition of methanol to the reaction mixture resulted in the synthesis of polyfunctional compound 250 in 79.1% yield containing a thiourea fragment, a thionamidophosphate ester group, and a free phosphate dibasic acid function (Scheme 66).^91^

**Scheme 67:**
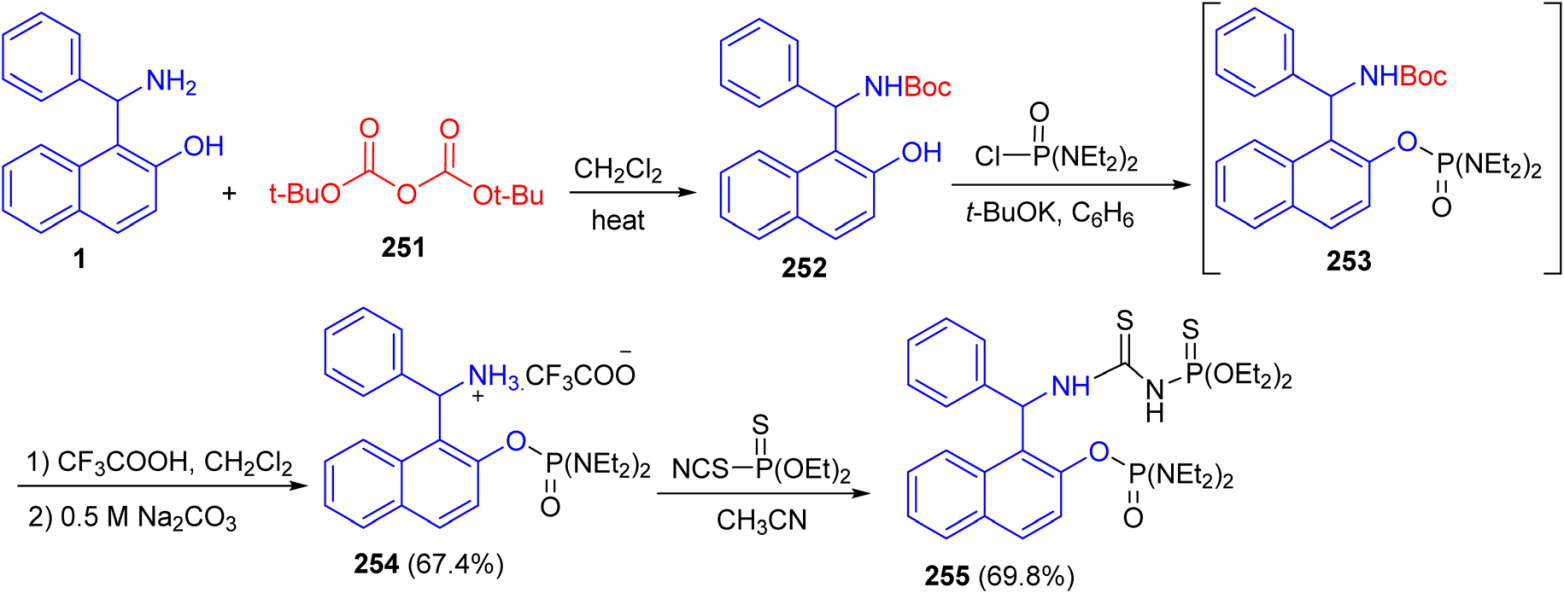
Synthesis of the Betti base phosphorylated 254 and 255.

In 2018, Alfonsov and co-workers described the reaction of the Betti base 1 with di-*tert*-butyl decarbonate (251) gave Boc-protection of the amino group of the Betti base 252. The product 252 which reacted with tetra ethyl diamidochlorophosphate in the presence of potassium *tert*-butylate to form compound 253. To remove the Boc protecting group, compound 253 without isolation was treated with trifluoroacetic acid to form trifluoroacetate 254 in 67.4% yield. The latter reacted with *O*,*O*-diethyl thiophosphorylisothiocyanate to give thiourea bearing a chiral Betti base fragment 255 (Scheme 67).^92^

**Scheme 68:**
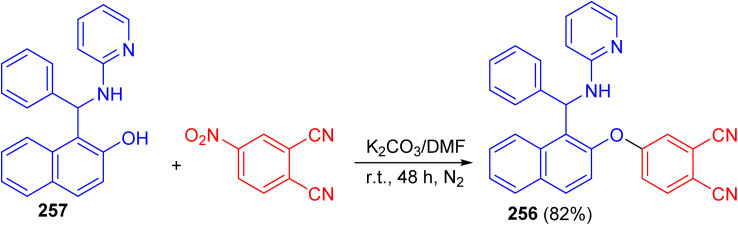
Synthesis of phthalonitrile 4-((1-(phenyl(pyridin-2-ylamino)methyl)naphthalen-2-yl)oxy)phthalonitrile 256.

After that, Vasudevan *et al.* described synthesis of phthalonitrile 4-((1-(phenyl(pyridin-2-ylamino)methyl)naphthalen-2-yl)oxy)phthalonitrile 256 in 82% yield by aromatic nucleophilic coupling reaction of 4-nitrophthalonitrile with phenyl-(2-pyridinylamino)-methyl-2-naphthalenol 257 in DMF using anhydrous K_2_CO_3_ at room temperature for two days under nitrogen atmosphere. The compound 256 exhibited a selective and sensitive fluorescence detection tendency towards chromium Cr^3+^ ion by the fluorescence turn on processes (Scheme 68).^93^

**Scheme 69:**
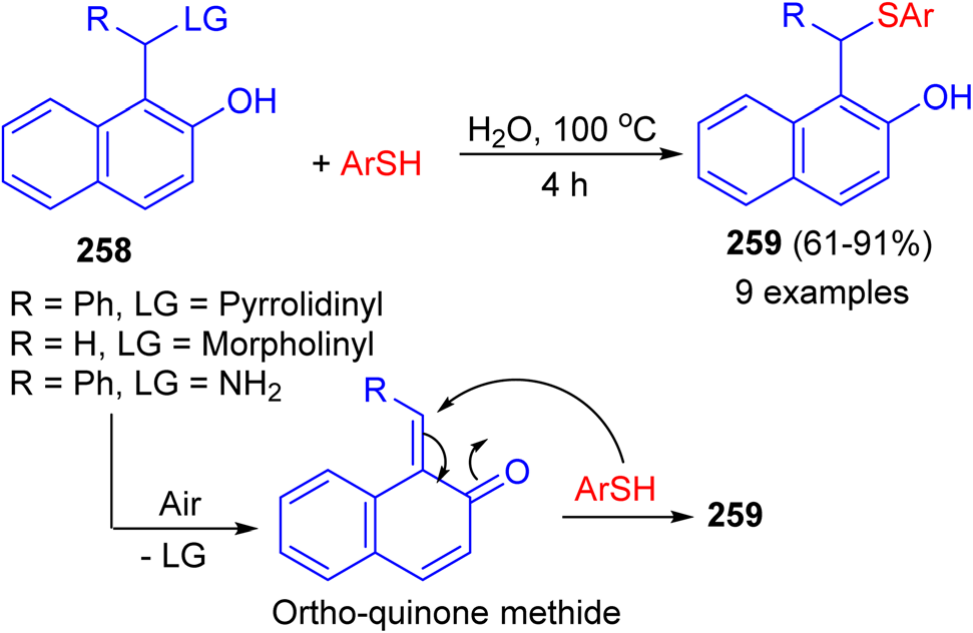
Synthesis of thioethers 259 from aminonaphthol.

In 2022, the treatment of aminonaphthol derivatives 258 with thiols under catalyst-free in water at 100 °C for 4 h afforded thioethers 259 in 61–91% yields. A plausible mechanism illustrated in Scheme 69. The aerobic oxidation of the aminonaphthol could result in the formation of *ortho*-quinone methide intermediate. The addition of thiol onto the *in situ* generated *ortho*-quinone methide could result in the formation of the desired product 259. Saturated brine solution was identified as a suitable medium for the synthesis of thioethers from aminophenol derivatives. Control experiments revealed that the nature of leaving group and reaction atmosphere plays a vital role in determining the yield of the products.^94^

**Scheme 70:**
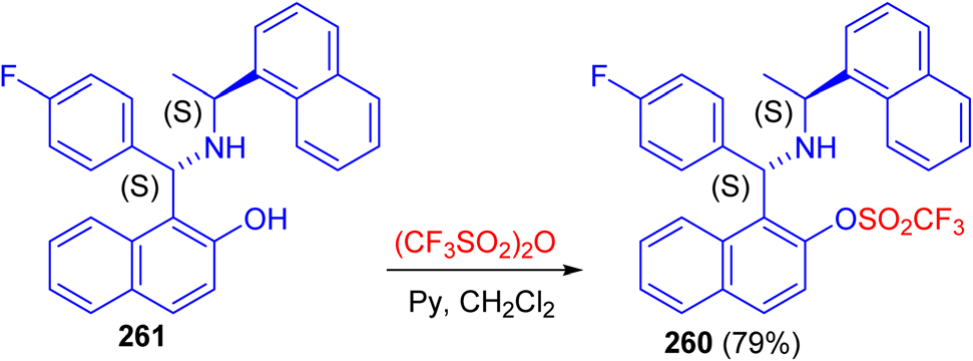
Triflation of the Betti base 261.

Recently, synthesis of 1-[(1*S*)-(4-fluorophenyl)-((1′*S*)-1′-naphthalen-1-yl-ethylamino)-methyl]-naphthalen-2-ol 260 in 79% yield reported by the triflation reaction of (*S*,*S*)-aminobenzylnaphthol 261 with trifluoromethanesulfonic anhydride in the presence of pyridine in dichloromethane at room temperature for 16 h as shown in Scheme 70. Compound 260 can be used as valuable intermediate in the future synthesis of aminophosphine, to be used in asymmetric catalysis.^95^

## 9 Conclusions

The Betti reaction is a multicomponent reaction that allows for the synthesis of various compounds containing C–C and C–N bonds in a single step. The Betti bases exhibit significant versatility as essential structural motifs in synthetic organic compounds, especially in the synthesis of heterocyclic molecules. This is attributed to their capacity for incorporating diverse functional groups, including –OH and –NH. This review explores transformations of the Betti base derivatives to the heterocyclic compounds such as naphthoxazines, bis-naphthoxazines, naphthopyrrolooxazines, naphthopyridooxazines, naphthooxazinoazepines, naphthoxazinobenzoxazines, naphthoxazinoisoquinolines, naphthoxazinoquinolines and the other fused-heterocycles. Furthermore, the review investigates the applications of these transformed compounds in relation to their pharmacological properties. We hope this review will promote the continued interest in the conversion of the Betti bases to the organic compounds and will provide a comprehensive and valuable insight to fill the gap in the reactions of Betti bases and their transformations into target compounds in the future.

## Conflicts of interest

There are no conflicts to declare.

## Supplementary Material
